# Association between soil organic carbon and calcium in acidic grassland soils from Point Reyes National Seashore, CA

**DOI:** 10.1007/s10533-023-01059-2

**Published:** 2023-07-07

**Authors:** Mike C. Rowley, Peter S. Nico, Sharon E. Bone, Matthew A. Marcus, Elaine F. Pegoraro, Cristina Castanha, Kyounglim Kang, Amrita Bhattacharyya, Margaret S. Torn, Jasquelin Peña

**Affiliations:** 1 Department of Geography, University of Zurich, Zurich, Switzerland; 2 Lawrence Berkeley National Laboratory, 1 Cyclotron Road, Berkeley, CA 94720 USA; 3 University California Davis, Davis, USA; 4 Stanford Synchrotron Radiation Lightsource, Menlo Park, USA; 5 Advanced Light Source, Berkeley, USA

**Keywords:** Organo-metal interactions, Soil organic carbon, STXM C NEXAFS, Micro-XANES, Acidic grassland soils, Complexation

## Abstract

Organo-mineral and organo-metal associations play an important role in the retention and accumulation of soil organic carbon (SOC). Recent studies have demonstrated a positive correlation between calcium (Ca) and SOC content in a range of soil types. However, most of these studies have focused on soils that contain calcium carbonate (pH > 6). To assess the importance of Ca-SOC associations in lower pH soils, we investigated their physical and chemical interaction in the grassland soils of Point Reyes National Seashore (CA, USA) at a range of spatial scales. Multivariate analyses of our bulk soil characterisation dataset showed a strong correlation between exchangeable Ca (Ca_Exch_; 5–8.3 c.mol_c_ kg^−1^) and SOC (0.6–4%) content. Additionally, linear combination fitting (LCF) of bulk Ca K-edge X-ray absorption near-edge structure (XANES) spectra revealed that Ca was predominantly associated with organic carbon across all samples. Scanning transmission X-ray microscopy near-edge X-ray absorption fine structure spectroscopy (STXM C/Ca NEXAFS) showed that Ca had a strong spatial correlation with C at the microscale. The STXM C NEXAFS K-edge spectra indicated that SOC had a higher abundance of aromatic/olefinic and phenolic C functional groups when associated with Ca, relative to C associated with Fe. In regions of high Ca-C association, the STXM C NEXAFS spectra were similar to the spectrum from lignin, with moderate changes in peak intensities and positions that are consistent with oxidative C transformation. Through this association, Ca thus seems to be preferentially associated with plant-like organic matter that has undergone some oxidative transformation, at depth in acidic grassland soils of California. Our study highlights the importance of Ca-SOC complexation in acidic grassland soils and provides a conceptual model of its contribution to SOC preservation, a research area that has previously been unexplored.

**Supplementary Information:**

The online version contains supplementary material available at 10.1007/s10533-023-01059-2.

## Introduction

The biogeochemical processes governing soil organic carbon (SOC) accumulation and its persistence are complex and multifaceted (Jones et al. 2020; Kleber et al. 2021; Schmidt et al. 2011). Metals such as iron (Fe), aluminium (Al), and calcium (Ca) are often linked to the persistence of SOC within a soil profile (Hall and Thompson 2022; Oades 1988). Yet, calcium (Ca) can play competing roles in SOC dynamics. It is key in processes relevant to organic carbon (C) decomposition through its function in microbial signalling, osmoregulation (Dominguez 2018; Nava et al. 2020) and as an essential cofactor for different enzymes (Dai et al. 2017; Ye et al. 2016). However, Ca can also play a role in the preservation of SOC through physical separation and sorption processes (Muneer and Oades 1989a; Oades 1988; Rasmussen et al. 2018). Specifically, Ca may contribute to the protection of SOC by mediating its physical isolation through aggregation processes and by mediating organo-mineral or organo-metal associations through cation bridging processes (as reviewed in Rowley et al. 2018). A suite of recent studies has investigated the relationship between Ca and SOC in more detail (e.g., Martí-Roura et al. 2019; Minick et al. 2017; Sowers et al. 2018b; Yang et al. 2020). For example, Sowers et al. (2018b) highlighted that Ca can play a significant role in enhancing organo-mineral interactions between ferrihydrite and dissolved organic carbon. More recently, Rowley et al. (2021) demonstrated that higher Ca content was associated with a doubling of mineral-associated SOC in soils that had developed under similar conditions. However, we still do not fully understand the biogeochemical nature of the association between Ca and SOC, and the mechanisms that drive this increase in mineral-associated SOC content.

In soils, Ca is positively correlated with the presence of calcium carbonate and soil pH (pH > 6) through carbonate equilibria (Lindsay 1979). Therefore, the association of Ca with SOC has predominantly been studied in soils containing carbonates ([Ca_1-x_Mg_x_]CO_3_; Oades 1988; Rowley et al. 2020; Virto et al. 2018), which are estimated to cover 30% of the Earth’s terrestrial surface (Chen and Barak 1982). The association between Ca and SOC has been under investigated in acidic soils as Ca is assumed to have a lower importance in acidic soil ecosystems (Rowley et al. 2018), where the correlation between effective cation exchange capacity (CEC) and SOC decreases at a soil pH < 5.5 (Rasmussen et al. 2018; Solly et al. 2020); in part, because of the lower relative concentration of Ca (relative to Fe or Al), and its competition with other positively charged minerals or cations for negatively charged functional groups (Solly et al. 2020; von Lützow et al. 2006). Several studies have investigated the role of CaCO_3_ additions (liming) on SOC content of acidic soils (Briedis et al. 2012; Carmeis Filho et al. 2017; Inagaki et al. 2017; Sridhar et al. 2022a), but these studies investigate the effect of carbonates on the soil C cycle (see Paradelo et al. 2015 for detailed review), which concomitantly increase pH. The Hubbard Brook calcium addition experiments utilised wollastonite application, which has a lesser effect on pH (CaSi_3_O_8_; 850–4250 kg Ca ha^−1^), and demonstrated that Ca addition reduced SOC mineralisation (soil pH 4–5; Groffman et al. 2006; Likens et al. 1998; Minick et al. 2017) and caused significant shifts in the microbial community (Sridevi et al. 2012) in acidic forest soils. Ambient levels of Ca in acidic grassland soils could also influence SOC decomposition and thus may warrant further investigation. If Ca has the capacity to play an important role in SOC preservation in all soils (from acidic to basic pH or at lower Ca concentrations), rather than simply being confined to calcareous soils, it would be of global significance.

Synchrotron-based analyses have been used to investigate the association between Ca and SOC in a small number of studies. Studies using scanning transmission X-ray microscopy coupled with C near-edge X-ray absorption fine structure (STXM C/Ca NEXAFS) spectroscopy have already identified a strong spatial correlation between C and Ca in different soils (Chen and Sparks 2015; Solomon et al. 2012; Sowers et al. 2018a; Wan et al. 2007). Solomon et al. (2012) combined STXM NEXAFS C K-edge and Ca L-edge analysis of an ultra-thin sectioned microaggregate to investigate the interfaces of organo-mineral and microbial structures, demonstrating numerous clustered zones of Ca and C association. More recently, Seyfferth et al. (2020) demonstrated that Ca had a strong spatial correlation with aromatic and phenolic C at > 40 cm depth in an acidic wetland sediment. However, this preferential association between Ca and C of a specific composition has not been verified in well drained upland soils. Moreover, the speciation of Ca in soils has been scarcely investigated (Li et al. 2020; Prietzel et al. 2021). Prietzel et al. (2021) recently curated a reference library that documents a range of different Ca bonding environments in soils using Ca K-edge X-ray absorption near edge (XANES) spectroscopy. These techniques have also been applied to synthesised Fe-humic acid-Ca aggregates to demonstrate the Ca can form bonds with carboxylic functional groups (Beauvois et al. 2020). However, to date, no study has combined these different synchrotron-based methods to investigate both the bonding environment and speciation of C (K-edge) and Ca (K-edge) in acidic soils. Thus, there is little knowledge about the chemical nature of this association or whether certain sources and types of SOC are preferentially associated with Ca beyond carboxylic functional groups.

In this study, we combined both bulk soil characterisation techniques with synchrotron-based spectro-microscopic methods to investigate Ca-C associations in three acidic soil cores (< 1 m deep). The soil cores that spanned a pH gradient (soil pH = 4.0–5.3) were taken from coastal grasslands at the Point Reyes National Seashore (hereafter Pt. Reyes), California. Bulk soil samples were characterised to determine their physico-chemical properties, including soil texture, pH, mineralogy, SOC content, exchangeable and trace / major elements. Subsequently, bulk Ca K-edge spectra were obtained for the core samples at three depths. We also used μ-X-ray fluorescence (μ-XRF) coupled with μ-X-ray absorption spectroscopy (μ-XANES; Ca K-edge) and STXM (Ca L-edge and C K-edge) NEXAFS to investigate the physical and chemical association of Ca and C (and other elements) at the same three depths. Due to the wide range of spectroscopic analyses employed, we restricted the sample size, such that this study represents an early investigation of the association between Ca and SOC in acidic grassland soils at various analytical scales. We hypothesised that Ca would contribute to the accumulation of SOC in these acidic soil environments, where it would be preferentially associated with more plant-like or less decomposed C (Rowley et al. 2021; Seyfferth et al. 2020); particularly with increasing pH, due to the reduced competition from H^+^ for organic functional groups or mineral exchange sites upon deprotonation.

## Methods

### Site setting

Samples were taken from Point Reyes National Seashore (Pt. Reyes), California (37°59′47″N, 123°0′54″W, 188 m elevation). The climate is sub-humid mesothermic, while the vegetation is dominated by annual and perennial grasses such as *Agrostis capillaris, Festuca arundinacea*, *Holcus lanatus,* and *Poa Pratensis* (Amme 2008). Californian grasslands are typically dominated by non-native species (Eviner 2016). Areas that were sampled were used previously for cattle ranching prior to the incorporation of the National Park (1962; Livingston 1995), and this could have promoted the installation of these non-native species throughout the sites. Point Reyes is geologically distinct from the surrounding area due to local tectonic activity, and its soils have formed in a diverse array of parent materials (see Galloway 1977 for more details).

**Fig. 1:**
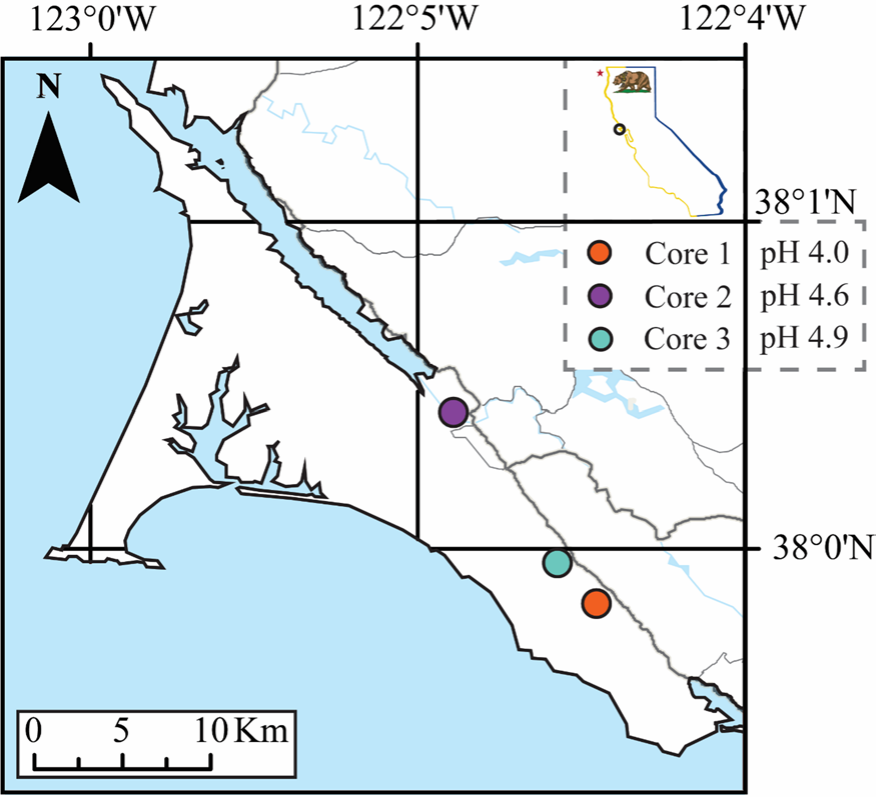
Core sample locations in Point Reyes National Seashore, California, USA. The mean soil pH is reported for each soil core as measured in 1 M KCl solution

We used existing soil surveys from the region (SoilWeb 2021) to select three distinct soil series which had all developed on sandstone, spanning a soil pH gradient (Fig. 1; soil pH [1 M KCl] 4.0–5.3). The soil cores were taken in slightly sloped (< 8°) terrain at the summit (Core 1), mid-slope (Core 2), or nearer the foot of a mild slope (Core 3), but sampling locations were specifically chosen in relatively flat regions to reduce the potential influence of slope. These soils were characterised as a Ferric Lixisol (Core 1; Tomales series, mesic Ultic Paleustalf), a Leptic Lixisol (Core 2; Blucher-Cole series, Fluvaquentic Haploxeroll) or an Abruptic Luvisol (Core 3; Yorkville series, thermic Typic Argixerolls) following the International Union of Soil Sciences Working Group World Reference Base (2015). This nomenclature translates to soils with a horizon containing illuviated clay with a high base cation saturation (Ca^2+^, Mg^2+^, Na^+^, K^+^), but low CEC that has either Fe or Mn nodules (Core 1) or that is shallow (Core 2); and finally, has a high base status illuviated soil with a high CEC and an abrupt textural change (Core 3). Core 1 through 3 are labelled with increasing soil pH (Fig. 1).

### Soil characterisation

Certain methods (internal standard details, X-ray diffraction and major/trace element compositions, linear mixed models, μ-XANES, STXM NEXAFS) are covered in more detail in the supplementary information (SI; Methods S1:5).

#### Preparation

Soil cores were sampled at *ca.* 10 cm intervals in November 2020 with a hand auger and transported to Berkeley Lab under ambient conditions for further analysis. Soil samples were dried at 40 °C for 24 h and sieved to 2 mm. Samples were corrected for hygroscopic moisture content according to van Reeuwijk (2002). A subsample from each soil sample was ground for 30 s in a Retsch MM400 ball mill at a frequency of 1800 rpm (30 Hz) for use in X-ray diffraction, X-ray fluorescence, and bulk XANES measurements as described below.

Three depth intervals were studied in detail, which corresponded to approximately 0–10, 30–40, and 60–70 cm (see SI for exact depth intervals for Cores 1–3). These depths were chosen as they represented distinct shifts in the soil profile (in soil texture, horizon, and organic carbon content) and helped us constrain changes in biogeochemical processes with depth in our soil cores. Hereafter, when we mention that a particular analysis was carried out at 3 depth intervals, we are referring to these depth intervals (*ca.* 0–10, 30–40, and 60–70 cm).

#### Soil characterisation

Soil pH was measured potentiometrically in a soil solution of 1 M KCl (1:2.5 soil to solution ratio) using a glass-body combination electrode (VWR 89231-574; Pansu and Gautheyrou 2006). Soil CHN analysis was completed on ground samples at Hilo Analytical Lab, University of Hawaii using a Costech 4010 Elemental Analyzer. Particle size distributions for each core sample were measured by laser diffraction using a Malvern Mastersizer 3000 with a Hydro LV module, samples were prepared using methods detailed in Pansu and Gautheyrou (2006). Soil exchangeable cations were extracted with a cobalt hexamine solution (16.6 mM; Aran et al. 2008). Extractants were diluted in 2% HNO_3_, combined with an internal standard (see SI), and measured using a Perkin-Elmer Sciex Elan DRC II inductively-coupled plasma mass spectrometer. The cation exchange capacity (c.mol_c_ kg^−1^) represents the sum of charge contributed by cations (Al^3+^, Ca^2+^, Mg^2+^, Na^+^, and K^+^), excluding H^+^. Quality control procedures included the analysis of an internal standard (see SI) when appropriate, as well as the inclusion of blanks and quality checks for all analyses.

Soil mineral composition, major- and trace-element composition of samples were determined at 3 depths as described in the SI (Methods S1). Soil mineral composition was characterised using synchrotron-based X-ray diffraction (XRD) at beam line (BL) 11–3, Stanford Synchrotron Radiation Lightsource (SSRL). Briefly, major and trace element compositions were determined from acid digestates using either an inductively-coupled plasma-atomic emission spectrometer or an inductively-coupled plasma mass spectrometer, respectively.

#### Soil statistical analyses

The effects of horizon and profile location on soil properties were tested using linear mixed models in SAS 9.4® as described in the SI (Methods S2). To account for autocorrelation, soil depth class was included as a repeated measures effect blocked by soil core, with a first-order autoregressive covariance structure (Simpson et al. 2010). Model structures were selected using the Bayesian Information Criteria. The significance of fixed effects was evaluated using type III F-tests. The means of significant fixed effects were compared using t-tests without multiple inference adjustment. The denominators’ degrees of freedom were computed using the Satterthwaite adjustment (Satterthwaite 1946). The alpha level of all reported means was set to *α* = 0.05. The reported means are all significant conditional least-square means ± the standard error of the mean. To characterise the relationships between variables and explore the covariability between variables, principal component analysis (PCA) was performed on the correlation matrix of our bulk characterisation data also using SAS 9.4®.

### Spectroscopy

#### Bulk X-ray absorption near-edge structure (XANES) spectroscopy

X-ray absorption near-edge structure (XANES) spectroscopy at the Ca K-edge can provide information on the average bonding environment of Ca in soil samples and was measured at 3 depth intervals. Spectra were also collected of reference materials with a known mineralogy and composition (Table S1). Samples were spread finely on Mylar® tape to prepare them for analysis. Samples that were too concentrated were diluted in boron nitride at a 1:10 ratio. The samples were measured at beam line (BL) 4–3 at the Stanford Synchrotron Radiation Lightsource (SSRL) using a Si (111) double-crystal monochromator and a beam size of 1 mm (vertical) × 2 mm (horizontal). Spectra were collected at room temperature in a He atmosphere in fluorescence mode using a seven-element Canberra Si-drift detector. Calibration was performed by setting the E max of the first derivative of a gypsum (CaSO_4_) standard spectrum to 4043.89 eV; calibration was checked every 8–24 h. At least three replicate scans were measured per sample and averaged prior to further analysis.

Averaged X-ray absorption spectra of the three replicate scans were background subtracted and normalised to an edge step of 1.0 in Athena (Ravel and Newville 2005) by fitting the pre-edge region with a linear function from 4013.39–4033.39 eV and the post-edge region with a second-order polynomial from 4063.39–4243.39 eV), and setting E0 to 4043.39 eV.

#### Micro-X-ray fluorescence (μ-XRF) imaging coupled micro-X-ray absorption near-edge spectroscopy (μ-XANES)

Micro-X-ray fluorescence (μ-XRF) imaging coupled micro-X-ray absorption near-edge spectroscopy (μ-XANES) provides information on the chemical arrangement of samples and the bonding environment of Ca in specific locations. The elemental distributions of thin sections taken from the cores at 3 depth intervals were mapped using μ-X-ray Fluorescence (μ-XRF) at BL 14-3b, SSRL. The bonding environment of Ca associated with specific elements was then analysed using Ca K-edge μ-XANES spectroscopy. Samples were embedded with Epotek 301 epoxy on quartz slides and thin sectioned at Grindstone Laboratory, Oregon. Thin sections were measured at room temperature in a He atmosphere. The beamline is equipped with a Si (111) monochromator. Sample fluorescence was detected using a 7-element Vortex detector. Energy calibration was completed by setting the E max of the first derivative of CaSO_4_ to 4043.89 eV.

Multi-energy maps (Table S2) were created at 2–3 different regions of interest using the methods outlined in the SI (Methods S3), and *ca.* 10 spectra were taken for each sample from the 3 depth intervals. The μ-XRF maps were analysed in SMAK and μ-XANES spectra were analysed in SIXPACK (Webb 2005). Spectra were then merged and normalised in Athena as described for bulk XANES analysis.

#### Scanning transmission X-ray microscopy C (K-edge) and Ca (L-edge) near-edge X-ray absorption fine structure spectroscopy (STXM C NEXAFS)

Scanning transmission X-ray microscopy coupled with C (K-edge) and Ca (L-edge) near-edge X-ray absorption fine structure spectroscopy (STXM C/Ca NEXAFS) provides information about the physical and chemical association of C, Ca, and Fe at the micrometre scale. Soil samples and a litter sample from the field site (see Table S1 for more details on litter sample) were measured STXM C/Ca NEXAFS at BL 5.3.2.2 of the Advanced Light Source. Samples from the 3 depth intervals of each soil core were spotted onto Si_3_N_4_ windows using methods adapted from Chen et al. (2014). Briefly, 25 mg of sieved soil was placed in an Eppendorf tube and vortexed with 1 mL of Milli-Q H_2_O (18.2 MΩ) for 10 s. After agitation, 1 μL of the sample suspension was pipetted onto windows, attached to a sample holder with a mild adhesive. Prior to data acquisition, energy calibration was performed using CO_2_ gas and setting the $1s\to 3s{\sigma }_{g}$ peak in the C K-edge to 292.74 eV (Prince et al. 1999).

All STXM C NEXAFS imaging and image analysis was completed in the STXM control program and STXM Image Reader (Marcus 2022) using the methods outlined in the SI (Methods S4). Briefly, STXM C NEXAFS stacks (a series of images obtained as a function of increasing photon energy over a space of x and y dimensions on the STXM window) were background subtracted for I0, positionally aligned, and mapped for C (295–280 eV), Ca (394.4–342 eV), and Fe (710–698 eV). These maps were created to identify regions of interest (ROI). From each ROI, two stack spectra were taken at the C K-edge and Ca L-edge for the 3 depth intervals of each core. Stacks were checked for saturation/thickness effects prior to further analysis. Calcium L-edge spectra are particularly susceptible to saturation effects due to variations in sample thickness (Hanhan et al. 2009) so regions in the stacks were masked to remove pixels with optical density values greater than 1, following Cosmidis et al. (2015). Linear correlations between the optical density pixel values of mapped elements were used to evaluate the micro scale spatial association between C, Ca, and Fe. Using a novel method, C and Ca stacks were subset using a Boolean function in STXM Image Reader to isolate the Ca and C XANES spectra corresponding to the overall C, Ca-C (no Fe), Fe–C (no Ca), or Fe-Ca-C signal (more details on image analysis procedure are found in SI Methods S4). All exported spectra were normalised in Athena. Briefly, a pre-edge line was subtracted through 279.8–283.3 eV. The C K-edge data were normalised by fitting a second-order polynomial to the post-edge spectral region (291.8–302.0 eV), setting the edge jump at 284.8 eV to an intensity of 1.0. Peaks were assigned to specific functional groups as summarised in Table S3.

#### Linear combination fitting of X-ray absorption spectra

Ca K-edge bulk XANES and μ-XANES spectra and C K-edge spectra were fitted using linear combination fitting (LCF) analysis. For Ca K-edge XANES, the set of reference spectra used in LCF was identified through PCA and target transform analysis. Target transform analysis (detailed in Methods S5) was used to test the likelihood that a given standard spectrum (Table S1; Fig. S1 & S2) could be reproduced from the principal components identified in the PCA of the soil samples (Malinowski 1978). The SPOIL values generated during target transform analysis measure how much the experimental sample set disagrees with the standard input and can be used to identify the best standard set for linear combination fitting analysis (see SI for more details). For STXM C NEXAFS spectra, LCF analysis was conducted on the total C signal using only the Fe–C or Ca–C subset spectra as standards to give us an approximation of the quantity of C associated with each of these metals.

## Results

### Bulk soil characterisation

We studied the physical and chemical association of Ca and SOC over various analytical scales in 3 soil cores, taken from acidic grassland soils. Unless specified otherwise, values reported in the results section below are mean values for a specific variable, which displayed significant differences (*α* = 0.05) between the cores, and are reported with the standard error of the mean.

**Fig. 2:**
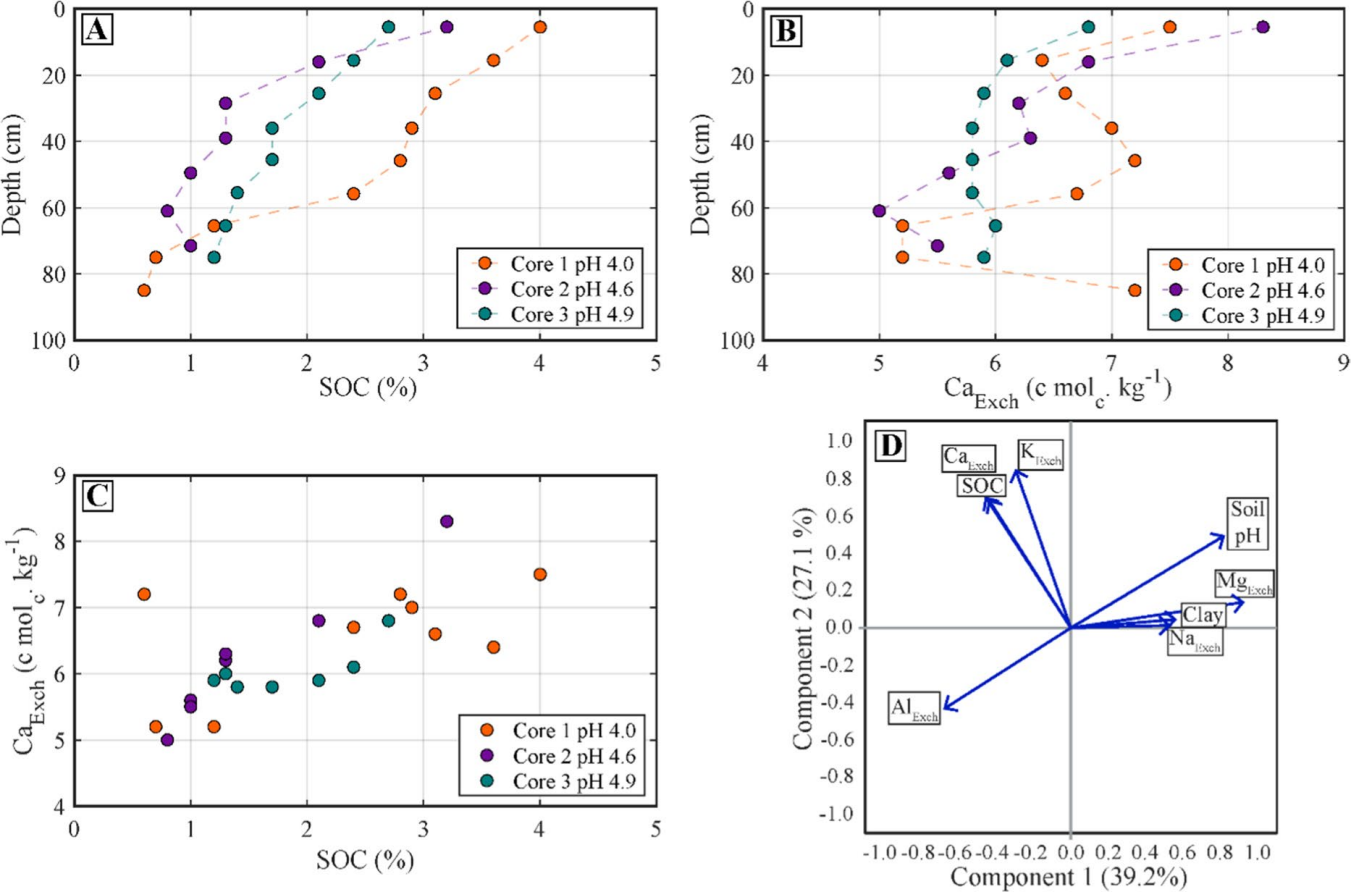
**A** Soil organic C content depth profile of the Point Reyes soil cores. **B** Exchangeable Ca content depth profile. **C** Positive correlation between SOC and Ca_Exch_ content **D** Principal component analysis results from the bulk characterisation analysis, ‘X’_Exch_ stands for the exchangeable element of X, quantified by cobalt hexamine extraction

Across all samples, soil texture became finer with depth, representing the development of an argic horizon (40–70 cm; Table S4). Soil texture also became finer across the cores moving from a combined clay and silt content of 57 ± 3% in Core 1 to 83.7 ± 3.2% in Core 3. Soil pH showed no significant change with depth (Fig. S3), but increased from Core 1 to Core 3, ranging from very acidic (soil pH = 3.8–4.8; Core 1–2) to moderately acidic (soil pH = 4.8–5.3 Core 3; Table S4). X-ray diffraction analyses suggested that soil mineral composition was dominated by phyllosilicates, quartz, and feldspars, with small concentrations of oxides, sulphates, and phosphates (Table S5). The main Ca-containing minerals measured in our soil cores were anorthite (< 20% w/w mineral matrix) and wollastonite (< 16%), with smaller, but ever-present contents of lazurite (< 9%; Table S8). Major and trace element contents for the 3 depth intervals of each soil core are presented in the SI (Table S6 and S7, respectively). Total Ca content ranged from 0.5–0.9% w/w, decreasing with depth. Total Fe increased (2.3–6.3% w/w) and total K decreased (1.4–0.8% w/w) from Cores 1 through 3. Soil organic C decreased from 4% to 0.6% with depth, and the average SOC content decreased from Core 1 (2.3 ± 0.1%) and Core 3 (1.7 ± 0.1%) to Core 2 (1.6 ± 0.1%; Fig. 2A). Base saturation (not including H^+^) was high in all samples (> 89%), increasing with pH from Core 1 to Core 3. The exchangeable pool of Ca displayed a positive correlation with SOC content (Fig. 2C; *R*^*2*^ = 0.69) both in the correlation matrix and PCA analysis (Fig. 2C, D Table S9 & S10). Overall, SOC was correlated with Ca_Exch_ even in these acidic grassland environments (Fig. 2C).

### XANES

#### Bulk Ca XANES

**Fig. 3:**
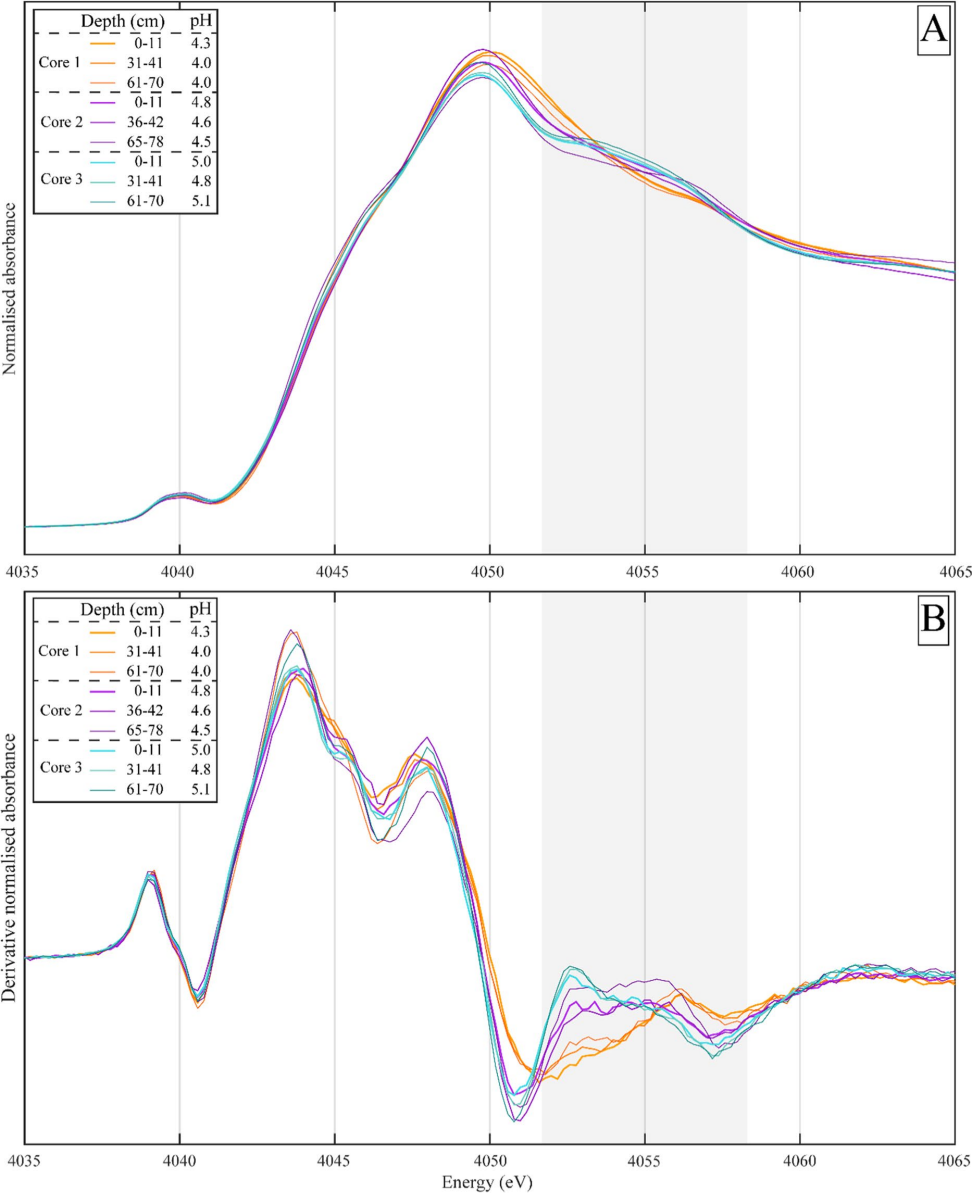
**A** Normalised bulk Ca k-edge X-ray absorption near-edge structure spectra of samples taken from three depths of each soil core at Point Reyes and **B** The 1st derivative of the normalised spectra. The shoulder feature at 4055 eV has been highlighted with a grey box

X-ray absorption near-edge structure spectroscopy at the Ca K-edge was used to investigate changes in the Ca bonding environment as a function of depth and pH across the sample set. Calcium K-edge XANES spectra were collected for more than 18 mineral and organic reference materials; their analysis is presented in the SI (Fig. S1 & S2). The Ca K-edge spectra from Ca-bearing minerals typically had a shoulder on the rising edge at 4045 eV; several mineral standards also had features centred at 4060 eV (Fig. S1 & S2). All our bulk sample spectra (Fig. 3A, B) shared a pre-edge feature centred on 4040 eV, which did not shift with pH or depth. The rising edge (4045 eV) of our bulk sample spectra, however, had a shoulder feature that became more pronounced with depth, shifting by at most − 0.2 eV, while the white line position (the point of highest absorbance around 4049 eV) displayed some pH dependence.

The white line position of the Ca K-edge spectra of bulk soil samples shifted towards lower energies (− 0.3 eV) with increasing pH (Fig. 3A, B). There were two convergence points at 4052 and 4056.9 eV (grey band Fig. 3A, B), implying a shifting contribution of two or more spectral end-members. Spectra from soils with a higher pH (> 4.4; Cores 2 & 3) also presented a broad ‘humped’ shoulder on the high energy side of the white line, centred around 4055 eV (hereafter the 4055 eV feature). The centre of this 4055 eV feature shifted to lower energies in the normalised 1st derivative of our spectra as pH increased from Core 1 (4056.3 eV) to Core 3 (4052.6 eV), highlighted in grey in Fig. 3B. This 4055 eV feature was centred at lower energies than other shoulder features in our mineral standard spectra set (*ca.* 4060 eV; Fig. S1). The 4055 eV feature could not be assigned to the presence (Core 2 & 3) or absence (Core 1) of Ca-bearing minerals identified in our XRD analysis (such as lazurite, wollastonite, or anorthite; Table S8) nor Ca exchanged montmorillonite.

#### Linear combination fitting analysis

**Fig. 4:**
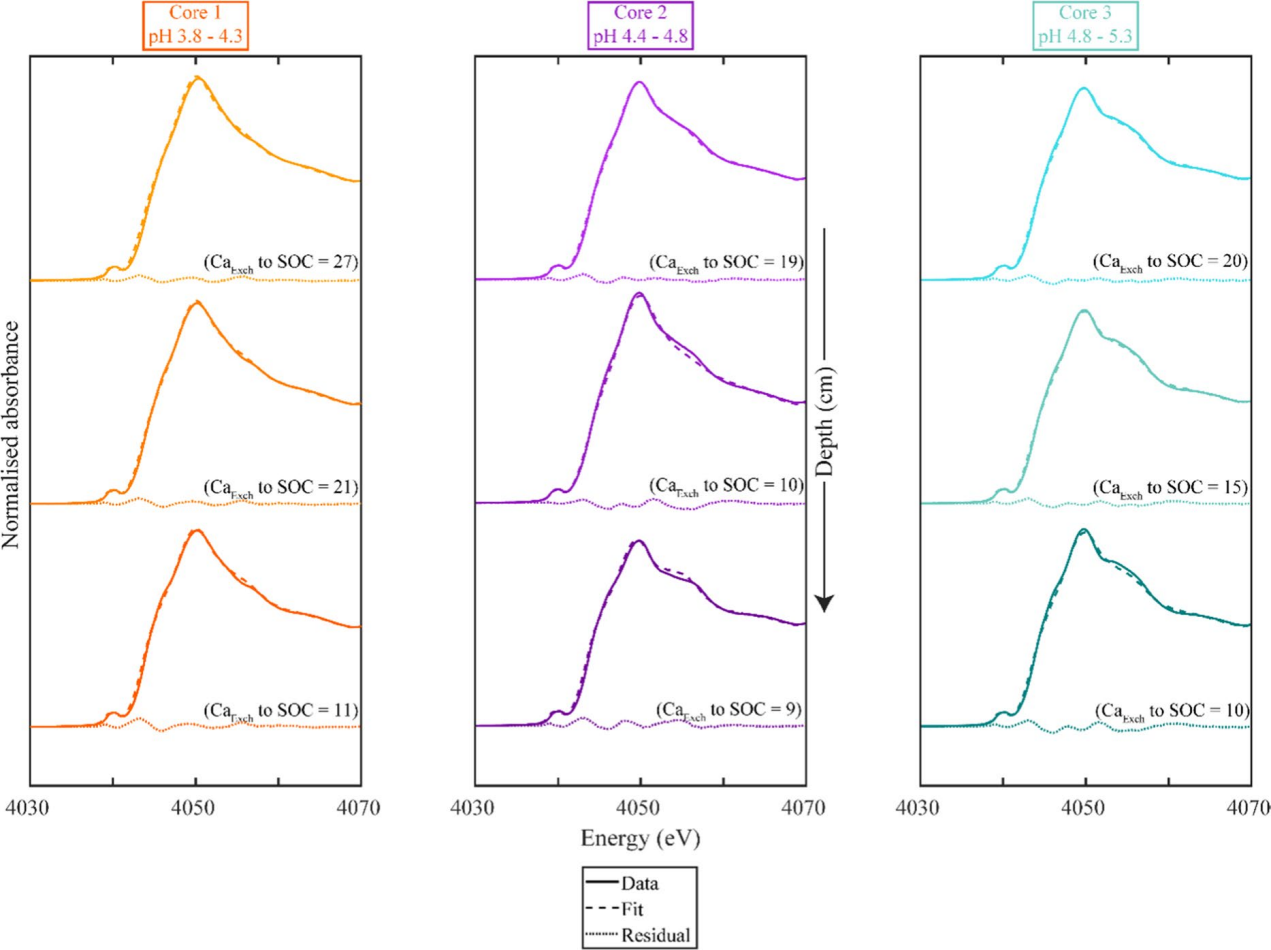
The linear combination fit (LCF) results for the Ca K-edge XANES spectra of our bulk soil samples, including a μ-XANES spectrum as a standard (Table S12). The spectra, in solid bold lines, are plotted together with the LCF results with a dashed line, while the residual is plotted beneath in a dotted line. The full results can be found in Table 1 below. The exchangeable Ca to SOC ratio of each sample is also included above the residual

A detailed discussion on the standards used in our LCF analysis are presented in the Suppl. Methods 5. All our standards failed to recreate the 4055 eV feature (Table S14). We thus added a μ-XANES spectra acquired from our samples that demonstrated this feature clearly (Table S12). The inclusion of this μ-XANES spectrum improved the LCF fit statistics and prevented the systematic increase in fit residual with soil pH. Our final LCF analysis standard set included Ca benzoate, Pt. Reyes litter, anorthite and a μ-XANES standard from Core 1.1 as standards (Fig. 4; Fig. S5; Table 1). These standards represent Ca bound to organic C, Ca in the existing litter, minerals, and a standard from the μ-XANES analysis with the 4055 eV feature, and were thus, good end members for the LCF analysis (Suppl. Methods 5).

**Table 1:** Linear combination fit results of the bulk Ca K-edge X-ray absorption near-edge structure spectra

Sample details		Fit results				Model statistics		
Core	Depth intervals	Ca benzoate	Pt. Reyes Litter	μ-XANES standard	Anorthite	R-factor		Reduced χ^2^
Core 1 pH 3.8–4.3	0–11	22.8 ± 0.01	44.4 ± 0.02	24.9 ± 0.01	8.0 ± 0.01	0.0004		0.0001
	31–41	21.3 ± 0.01	41.3 ± 0.03	28.0 ± 0.01	9.3 ± 0.01	0.0005		0.0002
	61–70	19.6 ± 0.02	26.0 ± 0.04	32.5 ± 0.01	21.9 ± 0.02	0.0009		0.0003
Core 2 pH 4.4–4.8	0–11	12.0 ± 0.01	47.2 ± 0.02	39.3 ± 0.01	1.6 ± 0.01	0.0005		0.0002
	36–42	6.1 ± 0.02	65.9 ± 0.03	25.4 ± 0.01	2.7 ± 0.01	0.0007		0.0003
	65–78	0.0 ± 0.02	35.3 ± 0.04	51.0 ± 0.01	13.7 ± 0.02	0.0013		0.0004
Core 3 pH 4.8–5.3	0–11	6.3 ± 0.01	43.4 ± 0.02	48.4 ± 0.01	1.9 ± 0.01	0.0004		0.0001
	31–41	11.3 ± 0.01	36.8 ± 0.03	49.6 ± 0.01	2.3 ± 0.01	0.0005		0.0002
	61–70	10.2 ± 0.02	37.6 ± 0.04	46.8 ± 0.01	5.5 ± 0.02	0.0012		0.0004

The μ-XANES standard refers to the Core 1 0–10 cm μ-XANES spectrum 2, which had the 4055 eV feature and the lowest SPOIL value

*The LCF weights were not forced to 100% during our analysis to check for potential fit error and ranged between 99 and 101%. The weights were then subsequently normalised to 100%

The fractional contribution of the anorthite spectrum to the sample spectra in our LCF analysis increased with depth (Table 1), which is consistent with the increase in the intensity of the shoulder on the rising edge with depth and overall increase in the proportion of mineral Ca as a function of depth. The residual of our LCF analysis did increase slightly with depth, which may be linked to an increasing proportion of mineral-associated Ca that was not completely accounted for by our reference set. The LCF analysis indicated that a large proportion (35–72%) of our bulk Ca K-edge spectra corresponded to Ca-organic complexes represented by the Pt. Reyes litter sample and Ca benzoate. The LCF weights for Ca benzoate decreased with depth in Core 1 and 2, but not Core 3. Similarly, the LCF weights for the Pt. Reyes litter sample trended towards lower values with depth. The μ-XANES standard accounted for between 25 and 51% of our spectra. There was also an overall trend towards increasing weights of this μ-XANES spectrum with increasing pH.

#### μ-XRF/μ-XANES

**Fig. 5:**
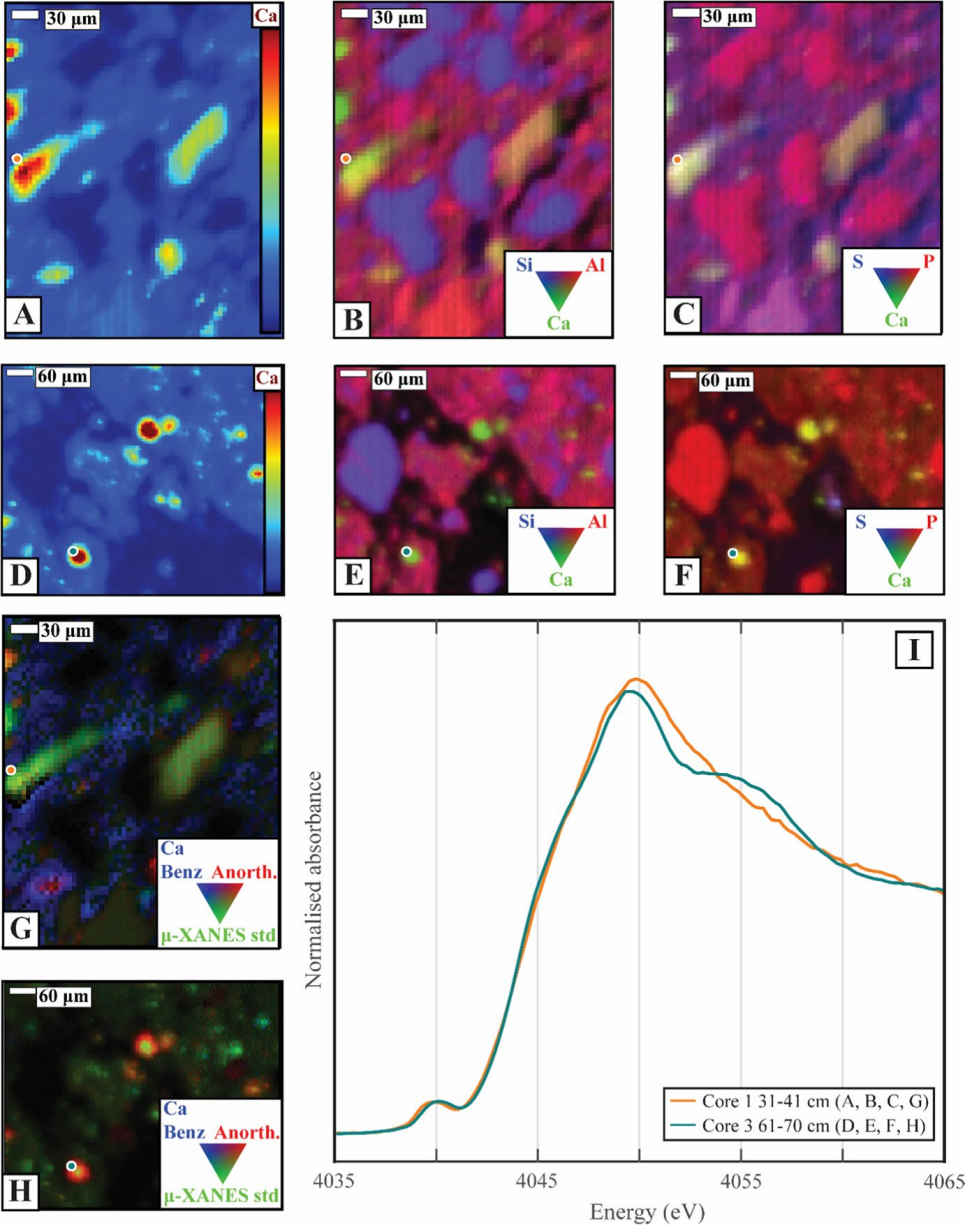
The presence of the shoulder in our μ-XANES spectra (4055 eV) could not be linked to a specific elemental association. **A** Ca content and, **B** tricolour of Si, Al, and Ca content and, **C** tricolour of S, P, and Ca content of Core 1 sampled at *ca.* 30–40 cm. **D** Ca content and, **E** tricolour of Si, Al, and Ca content and, **F** tricolour of S, P, and Ca content of Core 3 sampled at *ca.* 60–70 cm. **G**, **H** Least-squares fit of the multi-energy μ-XRF map using Ca benzoate, anorthite and µ-XANES standard from Core 1 sampled at *ca.* 30–40 cm **(G)** and core 3 sampled at *ca.* 60–70 cm (**H**). **I** Ca μ-XANES spectra taken from each sample at the coloured dot location in **A**–**H**

Micro-X-ray fluorescence and μ-XANES analyses were used to chemically map samples from 3 depth intervals and then investigate the bonding environment of Ca at specific locations in the sample. The chemical distribution of Ca did not display strong relationships (< 0.4) consistently with any of the measured elements (Al, Mg, P, S, and Si; Table S15). The strongest and most consistent relationship between Ca and a measured element in our μ-XRF maps was with Mg (mean adjusted *R*^2^ = 0.32), while other measured elements tended to have weak associations with Ca in different regions of interest (Table S15). A larger spectral variety was detected in our samples by μ-XANES analysis relative to the bulk spectra, presented in the SI (Fig. S6) as μ-XANES analysis focuses on a smaller region (2–5 μm spot size). Overall, 7 out of 58 μ-XANES spectra acquired contained the shoulder feature at 4055 eV (Table S12 & S14). Yet, the presence of this feature could not be correlated to a specific element (Al, Mg, P, S, and Si) in our μ-XRF maps (Fig. 5; Fig. S6:S12).

To explore further the 4055 eV feature and check whether certain elements correlated with Ca speciation across our multi-energy μ-XRF maps, we fit the XANES spectra to our μ-XRF maps using a least-squares fitting procedure. A few examples are presented in Fig. 5G, H, while all the results are presented in the SI (Fig. S7-12). In the Fig. 5 examples, the sample μ-XRF maps were fitted as either Ca benzoate (Fig. 5G) or a combination of anorthite and the 4055 featured μ-XANES spectra, which correspond with the spectra presented in Fig. 5I. The 4055 eV feature was fit on locations of high Ca content in our μ-XRF maps and was fit to a large area of the multi-energy μ-XRF maps, further justifying its inclusion as an endmember in our LCF analysis. The 4055 eV feature was also fitted over a larger proportion of the multi-energy maps with increasing pH, corresponding with our observations for the bulk XANES spectra (Fig. S7-12). However, we were unable to identify the origin of the 4055 eV feature in our samples.

#### STXM C NEXAFS

**Fig. 6:**
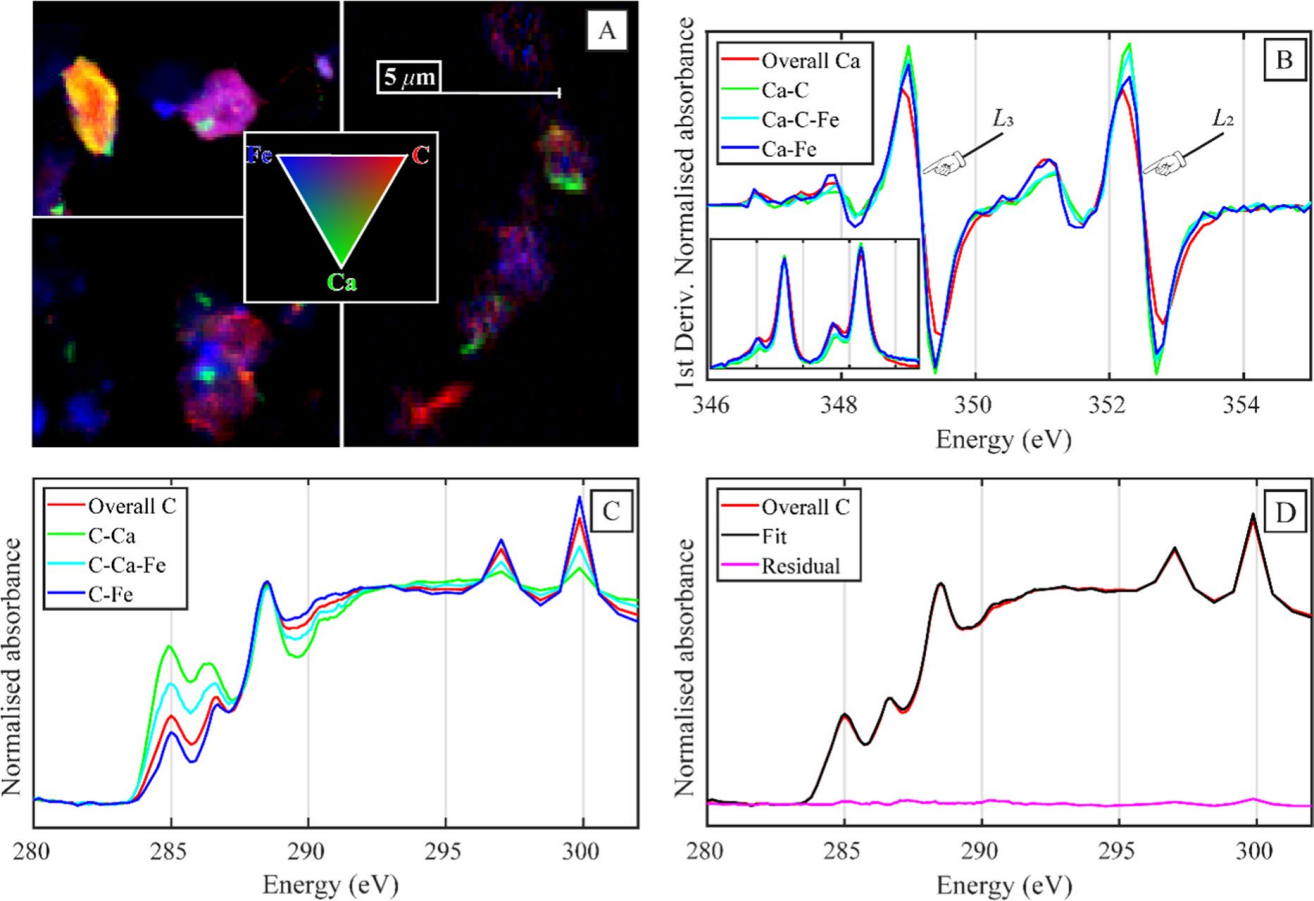
Averaged scanning-transmission X-ray microscopy (STXM C/Ca NEXAFS) results (*n* = 18 stacks). **A** Tricolour maps of three STXM C/Ca NEXAFS stacks with C in red, Ca in green, and Fe in blue. **B** Calcium L-edge normalised 1st derivative of merged subset spectra taken from the 3 soil cores, with the normalised Ca L-edge spectra inset in the bottom corner. Elemental associations were grouped through a Boolean classification method isolating Ca associated with C, Fe, or C and Fe. **C** Carbon K-edge STXM NEXAFS merged spectra averaged across all stacks (*n* = 18), grouped with the Boolean classification method. **D** Linear combination fit results of the overall C signal using the spectra from C associated with calcium and iron, which accounted for Ca = 23 ± 1% and Fe = 77 ± 1%, respectively

The physical and chemical association of C (K-edge) and Ca (L-edge) at the microscale was investigated using STXM C/Ca NEXAFS as well as a novel technique to isolate the signal specific to Ca-C association. While the samples were not fractionated by size a priori, STXM NEXAFS only examines clay and fine silt particles that are sufficiently thin to transmit the X-ray beam. As seen in Fig. S13 and Fig. S14, the positive linear correlation between the optical density values of Ca and C from all the pixels within our STXM C NEXAFS soil stacks was stronger (*R*^2^ = 0.45) than that between Fe and C (*R*^2^ = 0.05). The adjusted *R*^2^ value for the Ca-C association remained higher than the adjusted *R*^2^ value for the Fe–C relationship in 17 out of 18 stacks (Fig. S13). This relationship was also apparent in the tricolour maps, as illustrated in Fig. 6A.

We will now explore the spectral data from our STXM NEXAFS analysis, first focusing on the Ca L-edge data (346–355 eV) before moving to the C K-edge (280–295 eV). The Ca L-edge spectra of our samples were similar, regardless of the association of Ca with C, Fe, or C and Fe (Fig. 6B). Still, we observed small shifts in the *L*_2_ and *L*_3_ subpeaks that were dependent on elemental association. Specifically, the splitting ratio between the sub and principal peaks of Ca-C was smaller than that observed for Fe-Ca (Table S16).

The C K-edge spectra of our soil samples were clearly grouped by elemental association (Fig. 6B), more so than by core (Fig. S15) or depth (Fig. S16). The C associated with Ca displayed higher peak intensities in the aromatic C (285 eV), and phenolic group (286.4 eV) energy ranges, and decreased intensity over the O-alkyl C range (289.5 eV), relative to the total C, Fe-Ca-C, or Fe–C signal. The phenolic peak at 286.4 eV was also broader for Ca-C than Fe–C, Fe-Ca-C or the total C signal. The C associated with both Fe and Ca (Fe–C-Ca) had an intermediate spectral signal, displaying characteristics of both Fe–C and Ca-C. These patterns also emerged in the NNF analysis, which separated Ca-C and Fe–C as the principal forms of SOC in our STXM C NEXAFS stacks (analysis not shown). Yet, the Pt. Reyes litter samples showed no difference between the Ca-C and overall C signal (Fig. S17). Instead, when compared to the soil Ca-C signal, the litter sample displayed smaller peak intensities in the aromatic region and a broader peak in the carboxylic region (288 eV) of the STXM C NEXAFS spectra. Thereby, suggesting that the soil Ca-C association observed in the soil samples was not inherited from above-ground plant biomass that also contained less Ca (Fig. S18).

Overall, C associated with Ca had a less microbial and more plant-like spectral signature relative to Fe–C, even at up to 70 cm depth in these acidic grassland soils. Our LCF analysis of the STXM C NEXAFS total C spectrum in red (Fig. 6D), using the Fe–C (blue) and Ca-C (green) spectra as endmembers revealed that the total C signal could be reproduced by 77 ± 1% Fe–C and 23 ± 1% Ca-C. This suggests that a significant proportion (23%) of C in our soils displayed a spectral signature that was indicative of Ca-C association.

## Discussion

### The bonding environment of Ca

Calcium K-edge spectroscopy gave us novel insights into the bonding environment of Ca in acidic grassland soils. The proportion of mineral-associated Ca increased with depth in our soil cores, which is consistent with the reduced SOC content with depth and increasing proportions of Ca bound within minerals (Prietzel et al. 2021). However, the signal from the Ca K-edge spectra of samples were most like standards that were organic in nature, implying that Ca was closely associated with organic molecules in our acidic grassland soils. There was also an unidentified feature at 4055 eV, which increased with soil pH across our soil cores. Like soil pH, clay content and the total CEC (binding sites for Ca to bind to) increased from Core 1 through to Core 3. Yet, none of the Ca K-edge spectra from mineral standards with adsorbed Ca contained the same feature (Fig. S1). This suggests that this feature likely did not arise from the adsorption of Ca on mineral surfaces. It is still possible that an unidentified and unmeasured clay mineral with or without Ca adsorbed to its surface could be responsible for this feature. However, if this feature was crystalline, we would have expected this Ca containing mineral to appear consistently in the XRD analysis.

To our knowledge, the investigation of bulk Ca bonding environments in soils that have developed under different parent materials has only been attempted with Ca K-edge XANES spectroscopy by Prietzel et al. (2021). In this study, Prietzel et al. (2021) investigated two acidic soils (soil pH 1 M KCl = 3.4–5.0), which, from closer inspection, seemed to have a similar 4055 eV feature in their Ca K-edge XANES spectra. This spectral feature was attributed to augite or Ca oxalate, but augite was again not detected by our XRD analysis and Ca oxalate was ruled out because, although it had an excellent SPOIL value (1.2), it contributed negligibly to LCF. Calcium oxalate tends to be readily catabolised in soils (Cailleau et al. 2004, 2014) and is typically not measured at high concentrations in soils (Rowley et al. 2017). Thus, it seems as though augite and Ca oxalate are also not the direct source of the 4055 eV feature in our acidic soils setting.

We instead hypothesise that this 4055 eV feature could be organic in nature (Table S14), resulting from the complexation of SOC functional groups by Ca. This hypothesis is consistent with the fact that this feature was more accurately fit by our organic standards relative to our mineral standards (Table S14). Moreover, as this 4055 eV feature became more pronounced with increasing soil pH for our samples, we can speculate that Ca complexation was enhanced by deprotonation of the SOC functional groups. However, to resolve the nature of this 4055 eV feature in Ca K-edge XANES spectra more investigation would be required.

The STXM NEXAFS analysis only identified small differences in the Ca L-edge spectra, which were consistent with previously published spectra of organically bound Ca from soils (Solomon et al. 2012). Solomon et al. (2012) identified similar spectral features in their ultra-thin sectioned organo-mineral composite and its associated microbial structure with four principal peaks linked to the smaller crystal field and main spin–orbit peaks of the *L*_3_ (348.1 and 349.2 eV, respectively) and* L*_2_ edges (351.5 and 352.2 eV, respectively; Cosmidis et al. 2015; Solomon et al. 2012). Yet, similar spectra were also identified for Ca co-associated with Fe where an organic signal had been removed, this could be caused by trace amounts of C (under the optical density cut off) having a significant effect on the Ca L-edge. Larger shifts and a variety of spectral features have been reported in Ca L-edge spectra, when looking at a wider variety of minerals or standard compounds that span a broader array of Ca bonding environments such as within carbonates, phosphates, sulphates, and nitrates (Cosmidis et al. 2015; Fleet and Liu 2009; Naftel et al. 2001). There were small differences between the *L*_3_ and *L*_2_ splitting ratio, or distance between the principal and sub-peak of the Ca L-edge. Speculatively, as reported previously (Beniash et al. 2009) these differences could be evidence of reduced crystallinity of Ca bound to Fe, relative to Ca bound to C in our acidic system. Yet these results were not reproduced by Cosmidis et al. (2015) and this would require further investigation of different organics complexed with Ca. Overall, the bulk and μ-XANES Ca K-edge and STXM Ca L-edge data implied that Ca was closely associated with organic molecules in our acidic grassland soils.

### The association between bulk Ca and SOC

Soil organic carbon content was higher at our Pt. Reyes sites than a typical Californian grassland due to the presence of perennial grasses and coastal location, which drives elevated moisture levels relative to inland Californian soils (Corbin et al. 2005; Eviner 2016; Sanderman and Amundson 2009). The SOC content of our soils decreased with pH from Core 1 to Core 3, but SOC content was strongly correlated with Ca_Exch_ in our bulk soil core characterisation, supporting evidence from the spectral analyses. The content of SOC is commonly correlated with Ca_Exch_ content over large spatial scales, particularly in soils that have a pH > 6 (Rasmussen et al. 2018; Slessarev et al. 2016; Solly et al. 2020; Yuan et al. 1967). Yet, the correlation of SOC content with Ca_Exch_ is not causal evidence for the Ca-mediated stabilisation of organic matter.

The colocalisation of Ca_Exch_ with SOC could arise through several different processes, including: (i) the retention of exchangeable elements like Ca, on negatively charged functional groups of SOC or minerals (pH dependent; Edwards and Bremner 1967; Gaiffe and Schmitt 1980), (ii) the cycling of both elements by vegetation or microorganisms and the subsequent release of Ca during decomposition processes (Clarholm and Skyllberg 2013; Krieger et al. 2017; van der Heijden et al. 2015), and (iii) the finally the role that Ca can play in the preservation and accumulation of SOC, contributing to the occlusion of SOC or organo-mineral and organo-metal associations (Muneer and Oades 1989b; Rasmussen et al. 2018; Rowley et al. 2018). The correlation between these two elements on a bulk level in our acidic soil cores was likely caused by a combination of these mechanisms, a fact that emphasises the value of microscale investigations of their association.

### The association of Ca and C at the microscale

At the microscale, Ca was closely correlated spatially with C, consistent with previously reported microscale correlations (Chen et al. 2014; Keiluweit et al. 2015; Lutfalla et al. 2019; Solomon et al. 2012; Wan et al. 2007). The positive correlation between Ca and C has also been reported in the clay fraction of agricultural soils (Chen et al. 2014; Lutfalla et al. 2019) or a Phaeozem (Wan et al. 2007), where Chen et al. (2014) hypothesised that Ca plays an integral role in organo-mineral assemblage formation. The LCF analysis of the STXM spectra were also in agreement with the general understanding that Fe and Al dominate SOC association in acidic soil ecosystems, as the Fe C signal accounted for a larger proportion (77 ± 1%) of the total C signal (Hall and Thompson 2022; Rasmussen et al. 2018; von Lützow et al. 2006). Yet our results also highlight an unexpectedly large role of Ca in C association of acidic soil ecosystems and could challenge the hypothesis that soil pH can be used to delineate and model the dominant geochemical mechanisms driving SOC persistence in soils (Rowley et al. 2018).

### The association of Ca with C of a specific quality

The C K-edge spectrum associated with Ca in our samples, isolated through novel image analysis techniques, consistently had higher abundancies of phenolic and aromatic C and less O-alkyl C, relative to C associated with Fe. While this spectral signal could be inherited from plant cell walls in litter or root biomass that is preserved up to 70 cm depth, there was no real difference between the total C signal and Ca-C signal in our litter samples (Fig. S17), suggesting that this relationship is unlikely directly inherited from litter inputs and rather arises within the soil profile. The broader phenolic peak associated with Ca could either be driven by incomplete microbial oxidation of plant inputs or the influence of Ca complexation on peak positions (De Stasio et al. 2005), a topic that requires further investigation because of its potential to influence interpretations of C K-edge spectra. These results also corroborate recent STXM C NEXAFS measurements, where Ca was strongly correlated with C that had a more plant-like signal in an acidic wetland sediment (Seyfferth et al. 2020). Similar observations of Ca association with SOC with a plant-like signature have been reported in density fractions (Grünewald et al. 2006; Rowley et al. 2021).

The Ca-C spectra measured in our samples had some similarities with previously published spectra for lignin (Fig. S19; Karunakaran et al. 2015), differing in locations that are consistent with partial oxidative transformation. The degradation products of lignin do indeed have abundant quantities of phenolic and carboxylic groups, which can form stable (Schnitzer 1978), and preferential complexes with polyvalent cations like Ca^2+^ (Kaiser 1998; Römkens and Dolfing 1998). Qafoku et al. (2022) recently used different analytical methods and molecular dynamic modelling to investigate the association between calcite, Ca^2+^, and organic compounds, which included a lignin monomeric unit. The authors demonstrated a close association between Ca^2+^ and lignin, where ligands in the lignin monomer promoted the dissolution of calcite to form complexes with the released Ca^2+^. A close association with lignin and Ca has also been demonstrated in calcareous soils through density fractionation (Grünewald et al. 2006), where lignin-derived products had accumulated in the organo-mineral fraction (1.6–2.2 g cm^−3^). Grünewald et al. (2006) hypothesised that this accumulation was driven by the preferential adsorption of partly degraded lignin components in the organo-mineral fraction by Ca-containing layered double hydroxide minerals. Yet, lignin is rapidly decomposed by the extracellular enzymes of fungi in particular, but also bacteria in soils (Gleixner et al. 1999; Janusz et al. 2017) as indicated by the rapid loss of lignin biomarkers in many soils (Gleixner et al. 2002), which would thus inhibit its consistent association with Ca at depth, as measured in this study. We can instead hypothesise that SOC with a plant-like signature (Seyfferth et al. 2020), rich in different phenolic and carboxylic functional groups (Lehmann et al. 2020) is likely associated with Ca, relative to the C associated with Fe, which had a more microbially processed signature.

### Mechanisms for co-association of C and Ca

Although Ca-C complexes inherited from plant litter cannot be completely ruled out (Fig. S17), we can speculate that Ca could be contributing to the preservation of C with a specific biochemical composition due to their preferential association at up to 70 cm depth. As evidenced in the literature, in carbonate free soil, Ca can mediate the protection of SOC through physico-chemical mechanisms, including: the precipitation of dissolved organic matter from soil solution (Römkens et al. 1996), the complexation of different functional groups mediating organo-mineral (Rowley et al. 2021) and aggregation processes (Oades 1984), reducing its susceptibility to extracellular enzymes. Yet, Ca also plays an important role in decomposition pathways (Dominguez 2018; Nava et al. 2020) and the adherence of microorganisms to surfaces (Hemkemeyer et al. 2021). Recent work has demonstrated that Ca addition selects for surface-colonising or -adhering microorganism communities in limed soils (Sridhar et al. 2022a, b) or soils that were applied with wollastonite (Sridevi et al. 2012), suggesting these changes are not related to pH change alone. In a pre-print Shabtai et al. (2023) used Ca addition incubation experiments to demonstrate similar shifts in microorganism communities, which influenced carbon use efficiency, and the incorporation of litter into the mineral-associated fraction. In our soils, the O-alkyl C intensity of the Ca-C specific spectra was lower and thus presented a reduced polysaccharide content, it also had a less microbially processed signal relative to the Fe–C K-edge spectra. However, these mechanisms are not mutually exclusive and both could be operating in our soils, with further investigation now needed to understand when the biological or physico-chemical mechanisms driving Ca-C association might be dominant.

We can hypothesise that Ca may be contributing to the persistence of plant-like SOC in our acidic grassland soils and propose a conceptual model for this interaction. Conceptually, this could be initiated when plant litter is oxidatively transformed by microorganisms, decreasing its polysaccharide content, and increasing its relative proportions of functional groups (Lehmann et al. 2020; Lehmann and Kleber 2015). This interaction and the availability of Ca could indeed be selecting for specific microorganism communities with different carbon use strategies and efficiencies (Shabtai et al. 2023; Sridhar et al. 2022a, b). Once this plant-like SOC has undergone some oxidative transformation, retaining an aromatic, phenolic, and carboxylic spectral signature (Fig. 6C), it can then be bound by Ca to mineral surfaces increasing its incorporation into the mineral-associated SOC pool (Rowley et al. 2021) and its aggregation within physical structures or by itself (Muneer and Oades 1989b), changing its conformation. These processes could then reduce the accessibility of SOC to microbial extracellular enzymes, increasing its persistence, and thereby explaining the preferential association of Ca with SOC that has a specific spectral signature at the microscale.

## Conclusions

The current literature posits that Ca plays little role in SOC accumulation of acidic soil environments. In this study, we investigated the physical and chemical association between Ca and SOC in the acidic grassland soils (soil pH 4.0–5.3) of Pt. Reyes, over various analytical scales. Bulk characterisation, Ca K-edge XANES, and STXM C/Ca NEXAFS analysis clearly provided correlative evidence that Ca is closely associated with C in three separately sampled acidic soils. The XANES and μ-XRF/μ-XANES data further confirm that Ca was predominantly associated with organic carbon across all samples, with a bonding environment that was most similar to our organic standards. STXM C NEXAFS analysis revealed that Ca was preferentially associated with C that had higher relative abundances of aromatic/olefinic and phenolic C and a lower relative abundance of O-alkyl C compounds, even at depths of up to *ca.* 60–70 cm. Overall, our data suggests that Ca could be instrumental in the preservation and accumulation of partially transformed plant-like C in the acidic grassland soils of Northern CA.

## Supplementary Information

Below is the link to the electronic supplementary material.

Supplementary file1 (DOCX 18614 KB)

## Data Availability

The datasets generated and analysed in this study will be made available at ESS-DIVE (https://ess-dive.lbl.gov/) within 1 month of final publication.
