# Supplementary material for: Association between soil organic carbon and calcium in acidic grassland soils from Point Reyes National Seashore, CA
Source: Biogeochemistry. 2023 Jul 7;165(1):91–111. doi: 10.1007/s10533-023-01059-2 (PMC10457245; doi:10.1007/s10533-023-01059-2)
Supplement: Supplementary file 1 — Supplementary file1 (DOCX 18614 KB) [file 10533_2023_1059_MOESM1_ESM.docx]

**Supplementary methods: Internal standard details.**

A multi-element internal standard was created in ultra-pure nitric acid and used to correct ICP-MS measurements.

**Supplementary methods 1: X-ray diffraction and major / trace element compositions**

Major and trace element compositions: Major elemental compositions were measured at three depth intervals (*ca.* 0-10, 30-40, and 60-70 cm; see Table S4 for exact depth intervals) following lithium tetraborate fusion and subsequent dissolution of the fused discs at ALS Global (ME-ICP06). Solutions from the dissolved discs were measured using both inductively coupled plasma-atomic emission spectroscopy for whole rock elements (major / total) and -mass spectrometry for trace elements. Results were corrected for loss-on-ignition at 1050°C.

X-ray diffraction: X-ray diffraction was used to quantify the mineralogy of soil samples at three depth intervals (*ca.* 0-10, 30-40, and 60-70 cm; see Table S4 for exact depth intervals) and of ground mineral standards. Ground samples were loaded onto X-ray transparent Kapton^®^ tape and measured at λ = 0.98 Å over a Q-range of 0 to 5.75 at BL 11-3 in SSRL. Diffractograms were calibrated using a LaB_6_ (lanthanum hexaboride) standard, background subtracted for the beam stop and Kapton^®^ tape, and then converted from 2-D to 1-D using xdart software (Thampy, 2021). 1-D diffractograms were converted from Q to 2θ Cu Kα radiation equivalent (λ 1.54 Å) using Bragg’s Law. Quantification of the mineral composition was attempted following two methods. Rietveld refinement in GSAS2 returned unsatisfactory results due in part to the heterogeneous nature of our samples. Instead, mineral phases were identified using Xpert Highscore software and the PDF4-mineral database. Samples were semi-quantified using the rapid Reference Intensity Ratio method. Mineral standards, taken from the collection at University of California, Berkeley were also measured to validate the analysis and confirm the composition of standards used in the XANES analysis. Details of all measured standards are provided in Table S1 below. Trace quantities of dolomite ([Mg_1-x_Ca_x_]CO_3_) and ankerite ([(Fe, Mg, Mn)_1-x_Ca_x_]CO_3_) were detected (<1 %) at *ca.* 30-40 cm depth in Core 3. Yet, XRD analysis is inefficacious at detecting trace levels of carbonate (Loeppert and Suarez, 1996) and we can safely assume that our acidic soils (Soil pH < 5.3) were devoid of carbonates.

**Supplementary methods 2: Linear Mixed Models**

Model structures were selected using the Bayesian Information Criteria. Model residuals were plotted against predicted values to evaluate goodness of fit and any deviations from homoscedasticity. Residuals were checked for normality using QQ-plots (Galecki and Burzykowski, 2015). The significance of fixed effects was evaluated using type III F-tests. The means of significant fixed effects were compared using t-tests without multiple inference adjustment. The denominators’ degrees of freedom were computed using the Satterthwaite adjustment (Satterthwaite, 1946). Due to the low sample size, the stability of all significant effects was checked using different linear models (R, 2022) and SAS.

**Table S1:** X-ray absorption near-edge structure standard spectra details for the subset of 18 XANES spectra published below. All standards were prepared in ultra-pure MilliQ water (18.2 MΩ) in acid washed glass or clean plastic. All molar concentrations, unless otherwise specified, are L^-1^. Standards prepared by Sharon Bone can be found in (Niemann et al., 2023)

| **Standard** | **Chemical formula** | **Vendor** | **Synthesis** | **Purity** |
| --- | --- | --- | --- | --- |
| CaCl_2_ 0.01 M  Solvated Ca | Dissociated Ca^2+^ | ACROS organics  (10035-04-8) | This standard was created by Sharon Bone (18.2 MΩ). A solution of CaCl2 0.01 M was prepared in MilliQ water and measured in solution. | >99 %  CaCl_2_.2H_2_O |
| Amorphous calcium carbonate (ACC) | CaCO_3_ | Calcium chloride dihydrate (ACROS organics - 10035-04-8),  dimethyl carbonate (Sigma-Aldrich -  616-38-6),  sodium hydroxide (Sigma-Aldrich  1310-73-2) | This standard was created by Sharon Bone. 147 mg of CaCl_2_.2H_2_O (ACROS organics, 99+%) was combined with 450 mg of dimethyl carbonate (Sigma-Aldrich, 99+%) and 80 mL of MilliQ water (18.2 MΩ) at room temperature. Following this, 20 mL of 0.5 M sodium hydroxide (Sigma-Aldrich, >97%) was combined with MilliQ water and then added to the reaction solution while stirring. After 1 min, the reaction was stopped, and the precipitated solid was collected with centrifugation. The supernatant was discarded, and acetone was added to wash the precipitate. After washing, the precipitate was placed in a -80 ºC freezer to prevent conversion to calcite. This method was adapted from (Faatz et al., 2004) | >99 %  CaCl_2_.2H_2_O  >99 %  (CH_3_O_2_)CO  >97 %  NaOH |
| Gypsum | CaSO_4_ | Was purchased by Sharon Bone from Sigma-Aldrich | | 99 % CaSO_4_ |
| Apatite | [Ca_10_(PO_4_)_6_(OH)_2_] | Was purchased by Sharon Bone from Alfa Aesar | |  |
| Brushite | CaHPO_4_.2H_2_O | Was purchased by Sharon Bone from Santa Cruz Biotechnology Inc | |  |
| Wollastonite | CaSiO_3_ | Obtained from the University of California geological library courtesy of John Grimsich | | >95 % checked with XRD. |
| Anorthite | CaAl_2_Si_2_O_8_ |  |  |  |
| Montmorillonite (Californian) | SiO2: 52.8 Al2O3: 15.7, TiO2: 0.181, Fe2O3:1.06, FeO:  <0.10, MnO: 0.03, MgO: 7.98, CaO: 0.95, Na2O: 0.92, K2O: 0.03, P2O5: 0.02, LOI: 21.2> | Clay mineral society (SCa-3) | NA | https://www.clays.org/ sourceclays_data/ |
| Montmorillonite Ca exchanged  SWy-2 + CaCl_2_ | CaCl_2_ + SiO2: 62.9, Al2O3: 19.6, TiO2: 0.090, Fe2O3:3.35, FeO: 0.32, MnO: 0.006, MgO: 3.05, CaO: 1.68, Na2O: 1.53, K2O: 0.53,F: 0.111, P2O5: 0.049, S: 0.05, Loss on heating: -550°C: 1.59; 550-1000°C:4.47, CO2: 1.33 | Calcium chloride dihydrate (VWR 10035-04-8) + Clay Mineral Society (SWy-2) | Prepared by Mike Whittaker of LBNL. An aqueous diluent phase, (herein called clay-saturated aqueous phase, CSAP) saturated with respect to SWy-2, was made by dialysing SWy-2 (10 mg, <http://www.clays.org/sourceclays_data.html>) against MilliQ water (1 L, resistivity > 18.2 MΩ) for 1 week and filtering through a 0.02 μm filter (Whatman). To obtain homoionic MMT, SWy-2 (2-10 g) was dispersed in CaCl_2_ (1 M) in a polypropylene bottle (50 or 1000 mL) and mixed on a rotating mixer (5 rpm) for seven days. Sedimentation of coarse particles was achieved by centrifugation (1000 rcf for 10 minutes). Fine particles were separated from the coarse sediment and transferred to a cellulose dialysis membrane (Spectra/Por 3, 3.5 kDa molecular weight cut-off). Excess salt in the supernatant was removed by dialysis against CSAP, which was replaced every day for seven days. The resulting clay gel was dried in a convection oven (60°C for 14 hours, then 110°C for 6 hours). Suspensions were prepared by redispersing homoionic powder in either NaCl (1 M) or KCl (1 M) to a final concentration of 20 mg/mL and sonicating in an ultrasonic bath at 45°C for 24 hours. |  |
| Ferrihydrite Ca exchanged | CaCl_2_ + Fe(OH)_3_ (Fe(NO_3_)_3_ ·9H_2_O + NaOH) | Calcium chloride dihydrate (VWR 10035-04-8) + Iron (III) nitrate nonahydrate (Millipore Sigma 7782-61-8)+ Sodium hydroxide (Supelco; 1310-73-2). | 2-line ferrihydrite (Fe(OH)_3_, 385 m^2^ g^-1^) was synthesised according to the standard synthesis methods described in Schwertmann et al. (2004) and Schwertmann and Cornell (2008). Briefly, 40 g of was dissolved in 500 mM distilled water. 330 mM of 1 M NaOH was added to the suspension to increase the pH 7.5. The last 20 ml should be added dropwise with constant checking of the pH. The suspension was stirred vigorously for 30 min and centrifugated until the conductivity less than 15 ɥs cm^-1^ | 99-105 % CaCl_2_.2H_2_O |
| Fe-Mn Nodule (Core 1; Pt. Reyes) | NA | Pt. Reyes Experimental Site | Located at the Pt. Reyes Experimental field site in a pit dug adjacent to Core 1. | NA |
| Ca benzoate | CaCl_2_+ C_7_H_5_NaO_2_ | Calcium chloride dihydrate (VWR 10035-04-8) / Na benzoate (VWR) | A solution of 20 mM Na benzoate and 10 mM CaCl_2_ was shaken end-over-end for 16 h in the dark and measured in solution. | 99-105 % CaCl_2_.2H_2_O 99-101 % C_7_H_5_NaO_2_ |
| Ca citrate | CaCl_2_+ C_6_H_8_O_7_ | Calcium chloride dihydrate (VWR 10035-04-8) / Citric acd (Millipore Sigma  77-92-9; 201-069-1) | A solution of 20 mM Citric acid and 10 mM CaCl_2_ was shaken end-over-end for 16 h in the dark and measured in solution. | 99-105 % CaCl_2_.2H_2_O  > 99.5 % Citric acid |
| Ca phytic acid | CaCl_2_+ C_6_H_18_O_24_P_6_ · xNa+ · yH2O | Calcium chloride dihydrate (VWR 10035-04-8) / Phytic acid sodium salt hydrate from rice (Millipore Sigma 14306-25-3) | A solution of 20 mM Citric acid and 10 mM CaCl_2_ was shaken end-over-end for 16 h in the dark and measured in solution. | 99-105 % CaCl_2_.2H_2_O >99.9 % Inositol hexakisphosphate |
| Ca + Suwanee River Humic Acid Standard (SRHA) | CaCl2 + see <https://humic-substances.org/elemental-compositions-and-stable-isotopic-ratios-of-ihss-samples/> for more details on SRHA. | Calcium chloride dihydrate (VWR 10035-04-8) + Suwanee River Humic Acid – International Humic Substances Society (1S101H). | SRHA represents primarily wetland-derived aquatic humic substances. 196 mg SRHA was extracted in MilliQ H_2_O, increasing the pH to > 10.0 with NaOH, prior to reducing the pH to < 5.3 with HCl. Samples of a known volume were then combined with 10 mM CaCl_2_ and shaken end-over-end for 16 h in the dark. Samples were measured in solution. | 99-105 % CaCl_2_.2H_2_O  100 % SRHA  <https://humic-substances.org/ftir-13c-nmr-and-fluorescence-spectra/> |
| Ca oxalate | CaC_2_O_4_.H2O | Ca oxalate trace metal standard (NOVA Molecular Technologies, Inc.) | Ca oxalate ultra-pure standard was dried and ground in a Retsch MM200 ball mill, before being combined with boron nitride and measured at SSRL BL 4-3. | >99.999 % CaC_2_O_4_.H_2_O |
| Pt Reyes Litter | NA | Litter was collected at the Point Reyes Experimental Field site | Grass organic matter from different species collected through biomass cultivations at the Pt. Reyes core locations were air dried in the laboratory and then ground to a fine powder in a Retsch MM200 ball mill. Once ground samples were stored in glass vials and combined with boron nitride for dilution, prior to measurement at SSRL BL 4-3. | NA  (100 % litter) |

**Table S2.** Table listing the 7 spectral regions used to create multi-energy maps for the μ-XAS analysis.

| **Spectral region** | **Energy** |
| --- | --- |
|  | **eV** |
| 1 | 4044.18 |
| 2 | 4045.52 |
| 3 | 4047.67 |
| 4 | 4048.62 |
| 5 | 4050.47 |
| 6 | 4051.91 |
| 7 | 4055.15 |

**Supplementary methods 3: μ-XANES analysis**

Samples were first mapped at a coarse resolution (5 x 5 mm map, 10 μm resolution) to identify variations in Ca counts and the other detectable elements (Al, Mg, Na, P, and Si). Up to 10 Ca K-edge μ-XANES spectra were collected on a total of 10 spots (60 total) for each sample in our first run (Core 1.1, 1.4, 1.7, 2.1, 2.7, and 3.1). Locations for the initial 60 μ-XANES spectra were selected by masking pixel values in scatter graphs of Ca.Ka ~ other detectable elements (Al, Mg, Na, P, and Si). Principal component analysis (PCA) was then completed on these initial normalised spectra in Athena (*n* = *ca.* 60 spectra), and seven energies were selected based on peak maxima and minima of the first 4 significant components (Table S2). Following this step, we selected two to three regions of interest in each sample and remapped them at a higher resolution (5 μm) and at each of the seven Ca k-edge energies identified by the spectral PCA to create a multi-energy map in three different locations. We then applied simplex volume maximisation (SiVM) analysis to the multi-energy maps to identify different Ca spectral endmembers and take more μ-XANES spectra in each of these endmembers (*ca.* 10 spectra per thin section). Standards from our linear combination fitting analysis (described below) were fit to our multi-energy map using a Linear Least Squares fit (LS-Fit) in SMAK (V2.0). The error of the fit was checked by comparing the fit error against fitted values in correlation plots and by manually verifying intensities, normalised by overall intensities, against linear combination fit values. We also used SMAK V2.0 to compute regression statistics for the relationships between Ca and other elements or LS-fit standards from our μ-XRF maps.

**Supplementary methods 4: STXM C/Ca NEXAFS**

The I0 (blank background) was subtracted from each image and an image was taken above and below the energy of C (295-280 eV), Ca (394.4-342 eV), and Fe (710-698 eV), to create an elemental map. These maps were created to identify regions of interest for each stack. After identifying 2 mapped regions of interest on each sample, 2 stack spectra were taken at the C K-edge and Ca L-edge. Stacks were aligned with the maps to a cumulative average after filtering with a Sobel-Feldman operator in STXM Image Reader (Marcus, 2022). Stacks were checked for saturation / thickness effects and masked to remove affected regions, prior to further analysis. Calcium L-edge spectra are known to be particularly susceptible to saturation effects due to variations in sample thickness (Hanhan et al., 2009). To avoid these distortions in our Ca L-edge spectra we set a maximum optical density value of 1 (Cosmidis et al., 2015). We did not measure the Ca L-edge of the biomass sample as there was less Ca identified in the Pt. Reyes biomass, relative to the soil sample stacks (Ca OD mean biomass stacks = 0.05 ± 0.02 relative to the soil stacks = 0.13 ± 0.0; Suppl. Fig. S18)

Three different forms of analysis were completed on the aligned and saturation masked stacks. Firstly, the linear correlations between the optical density pixel values of mapped elements were used to evaluate the micro-scale spatial correlation between C, Ca, and Fe. Secondly, C and Ca stacks were subset using the Boolean function in STXM Image Reader. The Boolean function works by classifying and then masking (or performing another function) stack spectra by specific OD values, for instance, masking out all stack pixels that have an OD > 0.05 to remove background spectral noise or separating all pixels that have a Ca OD < 0.2. This function was used to isolate the C XANES spectra corresponding to the overall C signal as well as the C XANES spectra associated to pixels containing Ca-C (no Fe), Fe-C (no Ca), or Fe-Ca-C. Thirdly, stacks were analysed using principal component analysis (PCA) and non-negative matrix factorisation analysis (NNF). The endmembers identified by NNF were then fitted to the entire (unmasked) data set with a Linear Least Squares fit. The fitted NNF endmembers were then compared with elemental correlations using tricolour maps, linking spectral references to different elements using tricolour maps, created in STXM Image Reader. All exported spectra were normalised in Athena and then exported to Matlab^®^ for plotting.

Once our spectra were exported and normalised, we inferred which carbon functional groups were present in our samples using the values in Table S3 with values taken from the literature (Bone et al., 2017; Lehmann et al., 2009; Sedlmair, 2011).

**Table S3.** Carbon functional groups were assigned using values published within the literature (Bone et al., 2017; Lehmann et al., 2009; Sedlmair, 2011).

| **Peak energy (eV)** | **Organic C forms (bond)** | **Assigned transition** | **Examples** |
| --- | --- | --- | --- |
| 284.9 – 285.5 | Aromatic / Olefinic C (C=C) | 1*s* → π* | Protonated and Alkylated aromatic C  Carbonyl substituted aryl C  Alkene C |
| 286.0 – 287.4 | Aromatic C with side chain and N-substituted aromatic C  (C-OH, C=O, R-(C=O)-R, C=N, C-N) | 1*s* → π* | Carbonyl C in aromatic ring  Aromatic C attached to amide group  Phenolic C  Carbonyl C  Pyrimidine C |
| 287.0-288.5 | Aliphatic C (C-H) | 1*s*-3*p/σ** | Aliphatic C of CH_3_, CH_2_, and CH nature |
| 288.0-288.7 | Carboxylic C  (R-COOH, COO, C=O, NH_2_-C-O) | 1*s* → π* | Carboxylic C  Carboxyamide C  Carbonyl C |

**Supplementary methods 5: Linear combination fitting analysis**

Our STXM and Ca K-edge (XANES / μ-XANES) spectra were fit using linear combination fitting (LCF) analysis. The LCF analysis of STXM data was conducted on the total C signal only, using the Fe-C or Ca-C subset spectra as the end members to give us an approximation of the quantity of C associated with these metals across our STXM stacks.

We also completed LCF analysis on our bulk Ca K-edge spectra. Target transformation analysis was used to test the likelihood of a given standard spectrum explaining the variance in our sample spectra, and thus, its inclusion in our LCF analysis. Calcium K-edge spectra were first imported into SIXPACK. The older version of SIXPACK algorithm produced the more accurate SPOIL values due to a recent change in the algorithm, so V. 0.68 was used for target transform analysis. A PCA was then completed on our normalised sample spectra, which was used to complete target transform analysis on all 40 of our standard spectra. Fits were first checked visually, using a database of 40 standards, prior to removing any standards that were obviously not represented by our bulk XANES or μ-XANES datasets. This gave us a subset of 18 mineral and organic standards which are covered in Table S1. We then used the target transform analysis in the older version of SIXPACK to generate R factor (sum of squares of the differences between the data and the fit at each data point, divided by the sum of the squares of the data at each corresponding point), Chi-squared (χ2), and SPOIL values (Malinowski, 1978) for the standards in Table S1 and Fig. S1 & S2 over a range of energy intervals, with differing quantities of principal components. The results of these target transform analyses are presented in Table S11:13. According to Malinowski (1978) an excellent SPOIL value ranges between 0-1.5, good values 1.5-3, which are all acceptable, a fair value ranges between 3-4.5, poor values 4.5-6, which are moderately acceptable, and anything with a value > 6 is not acceptable.

These analyses revealed four facts:

1. The SPOIL values of our μ-XANES spectra were far higher than our bulk XANES spectra (Table S11).
2. Organic standards best fit our data, with high SPOIL values for our mineral standards (Table S11 & Table S5).
3. Our data were best fit over an energy interval of 4030-4070 eV.
4. A specific shoulder feature, centred at 4055 eV and described in the results in more detail, was not adequately fit by our bulk XANES standards, but was present in numerous μ-XANES spectra. We completed a separate target transform analysis on all of these 4055 eV featured μ-XANES spectra (Table S12), identifying a multi-energy spectrum from Core 1 sample 0-10 cm as the best fit.

We thus focused our LCF analysis on our bulk XANES data. After trying all the combinations of fits using our organic standards with and without this μ-XANES spectra (1.1.2), we found that an inclusion of 3 standards gave the best fit (presented in the results section in Table 1). With these insights we completed LCF analysis of our normalised bulk XANES spectra using the Ca benzoate, Pt. Reyes litter standard and 1.1.2 μ-XANES spectra in Athena over an energy interval of 4030-4070 eV. Results were exported for plotting in Matlab.

Target transform analysis using 4 principal components identified from the Ca K-edge XANES spectra of our soil samples (n = 9) indicated that our sample spectra were more similar to several organic standards than most of the mineral standards (Table S11-S14). While gypsum had a SPOIL value of 3.01 (see SI for more details), we did not consider it as a suitable reference because it was not identified by XRD (Table S8). Similarly, montmorillonite exchanged with Ca had a good SPOIL value (SPOIL value = 2.25); but was excluded as it was rarely identified by our XRD analysis, and its target transform residual was larger than other similar or higher SPOIL scoring standards (Fig. S4). We could not be sure of the exact composition or Ca bonding environment of the Fe Mn nodule from Pt. Reyes (SPOIL value = 1.73), so it was likewise excluded. Calcium oxalate had an excellent SPOIL value in the target transform analysis (SPOIL value = 1.2), but it was rarely identified by our LCF analysis. anorthite (SPOIL value = 1.86) and wollastonite (SPOIL value = 1.69), which were identified by XRD analysis as being the predominant Ca containing minerals in our samples, also had good SPOIL values in the target transform analysis. Both minerals were included as standards in the fits but, as with Ca oxalate, wollastonite contributed negligibly to the LCF analysis of the bulk spectra and was thus removed from the final standard set (Fig. S5).

All our standards failed to recreate the 4055 eV feature (Table S14). We thus added a μ-XANES spectra acquired from our samples that demonstrated this feature clearly (Table S12). Again, using the principal components from the Ca K-edge of bulk soil samples, we completed target transform analysis on the μ-XANES spectra (Table S12) that featured the shoulder at 4055 eV and selected as a standard the μ-XANES spectrum 2 from Core 1 0-10 cm (hereafter μ-XANES standard). We also completed target transform analysis on these μ-XANES spectra using a mineral and organic standard subset (Table S14), demonstrating that organic standards more accurately recreated the humped feature, with lower SPOIL values in most samples.


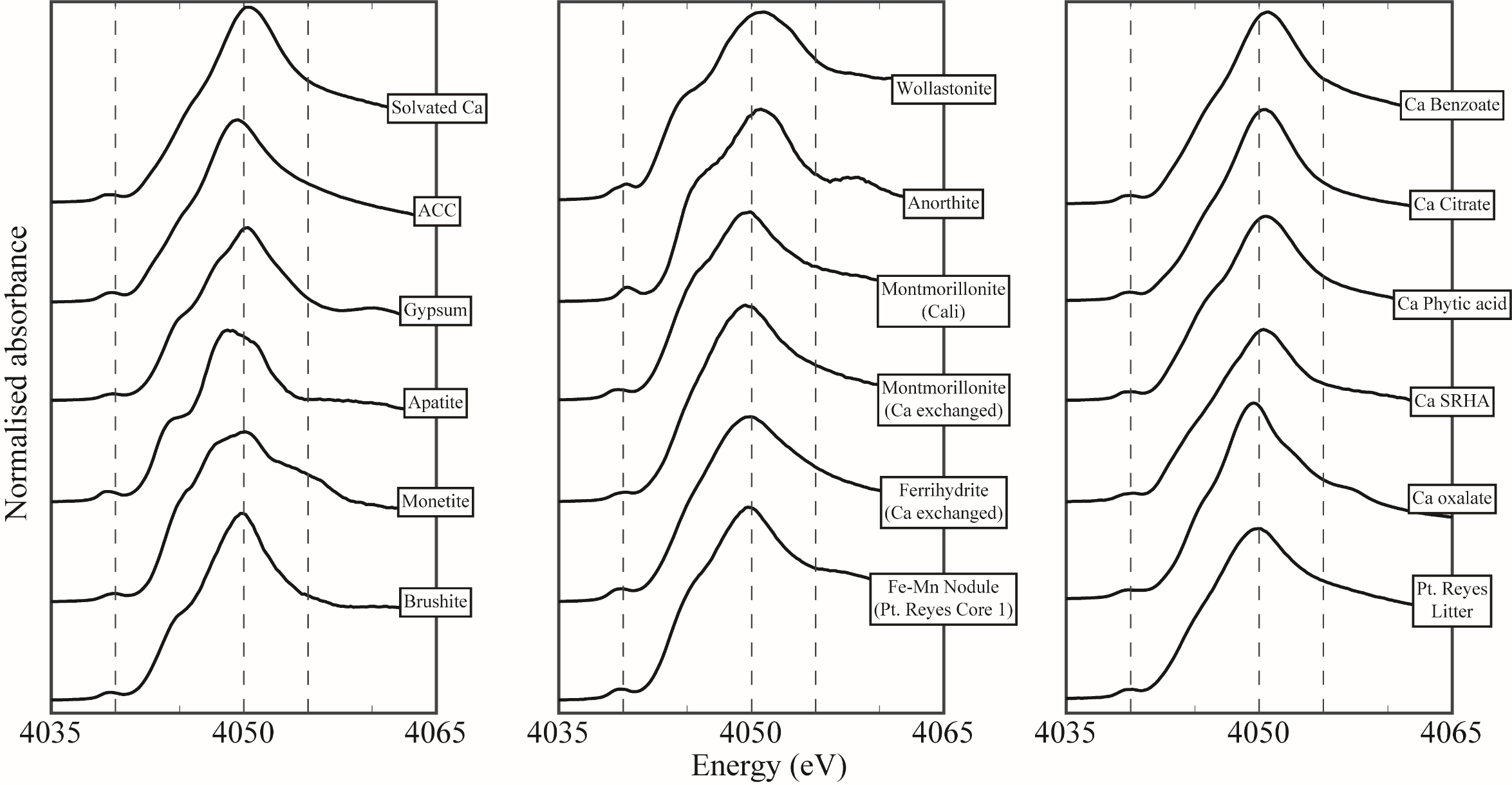


**Figure S1.** The Ca K-edge XANES spectra of the 18 reference standards (Table S1) used for target transform analysis. Spectra were all normalised in Athena.


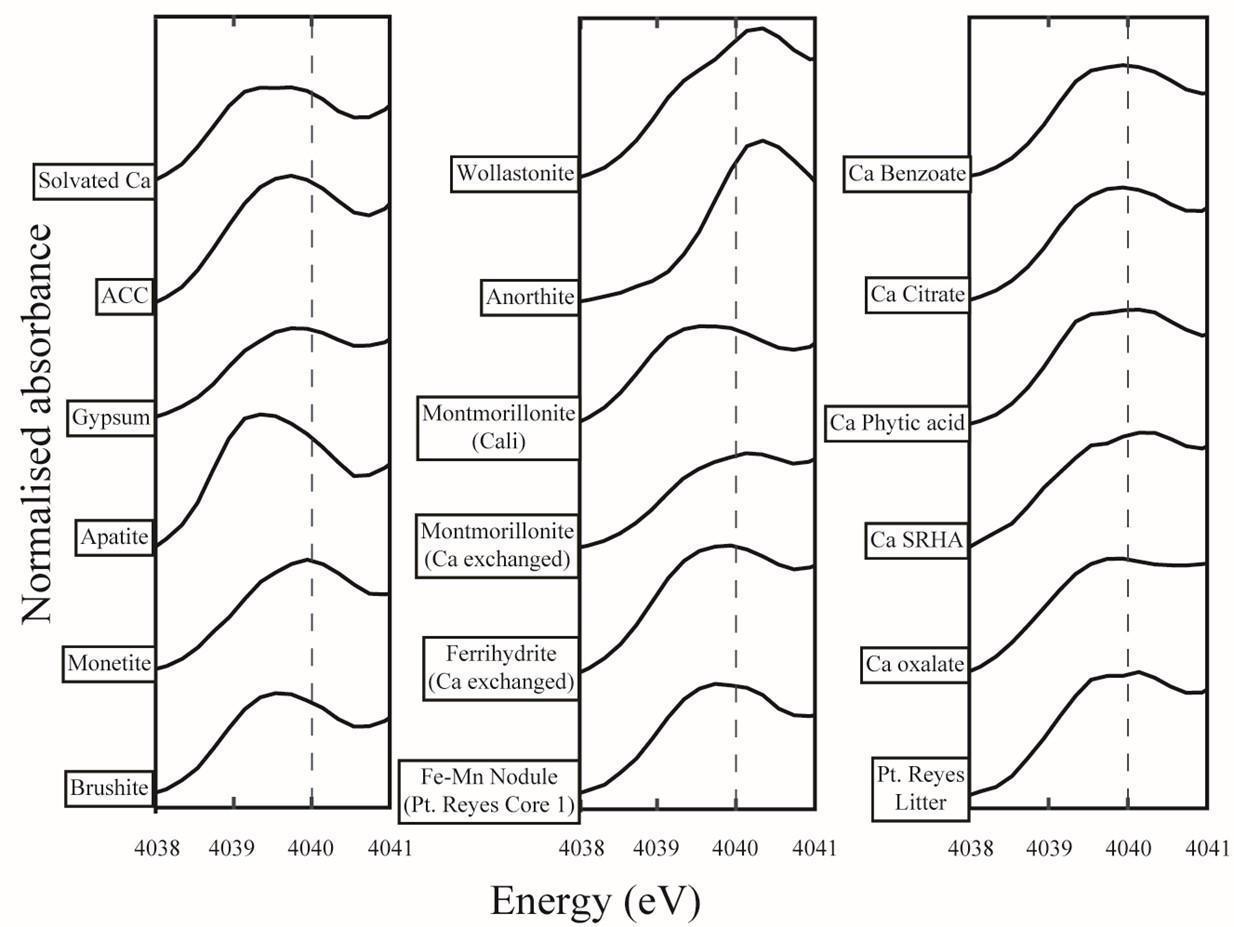


**Fig. S2.** The pre-edge feature of normalised Ca K-edge XANES spectra from the 18 standards (Table S1) used for the target transform analysis. All spectra were normalised in Athena.

**Table S4.** Bulk soil characterisation properties of the acidic grassland soil cores at Point Reyes.

| **Sample** | **Depth** | **Soil pH** | **Clay** | **Silt** | **Sand** | **SOC** | **Total N** | **C:N** | **Al_Exch_** | **Ca_Exch_** | **K_Exch_** | **Mg_Exch_** | **Na_Exch_** | **Sum** | **C:Ca ratio** |
| --- | --- | --- | --- | --- | --- | --- | --- | --- | --- | --- | --- | --- | --- | --- | --- |
|  | **cm** | **1 M KCl** | **%** | **%** | **%** | **%** | **%** | **Ratio** | **c mol_c_. kg^-1^** | | | | | |  |
| **Core 1.1** | 0-11 | 4.31 | 17.1 | 33.8 | 49.2 | 4.0 | 0.3 | 12.1 | 0.3 | 7.5 | 0.9 | 3.2 | 0.1 | 12.0 | 26.7 |
| **Core 1.2** | 11-20 | 4.01 | 17.3 | 33.6 | 49.1 | 3.6 | 0.3 | 11.7 | 0.9 | 6.4 | 0.5 | 2.3 | 0.0 | 10.0 | 27.9 |
| **Core 1.3** | 20-31 | 3.96 | 17.9 | 34.6 | 47.5 | 3.1 | 0.3 | 11.8 | 1.1 | 6.6 | 0.3 | 2.3 | 0.0 | 10.2 | 23.4 |
| **Core 1.4** | 31-41 | 4.03 | 18.1 | 32.9 | 49.0 | 2.9 | 0.2 | 12.0 | 0.9 | 7.0 | 0.2 | 2.5 | 0.0 | 10.7 | 20.5 |
| **Core 1.5** | 41-51 | 4.10 | 29.1 | 49.7 | 21.1 | 2.8 | 0.2 | 12.2 | 0.7 | 7.2 | 0.1 | 2.7 | 0.0 | 10.8 | 19.1 |
| **Core 1.6** | 51-61 | 4.08 | 17.8 | 34.0 | 48.2 | 2.4 | 0.2 | 12.0 | 0.9 | 6.7 | 0.2 | 3.0 | 0.4 | 11.1 | 17.6 |
| **Core 1.7** | 61-70 | 3.96 | 18.4 | 36.7 | 44.9 | 1.2 | 0.1 | 10.7 | 0.9 | 5.2 | 0.1 | 2.7 | 0.0 | 8.9 | 11.3 |
| **Core 1.8** | 70-80 | 3.82 | 19.2 | 40.3 | 40.5 | 0.7 | 0.1 | 8.4 | 0.8 | 5.2 | 0.1 | 4.0 | 0.0 | 10.1 | 6.3 |
| **Core 1.9** | 80-90 | 3.75 | 16.6 | 45.2 | 38.2 | 0.6 | 0.1 | 7.8 | 1.1 | 7.2 | 0.1 | 5.9 | 0.2 | 14.4 | 4.2 |
| **Core 2.1** | 0-11 | 4.84 | 11.9 | 41.1 | 47.0 | 3.2 | 0.3 | 12.1 | 0.0 | 8.3 | 0.7 | 6.0 | 0.1 | 15.1 | 19.2 |
| **Core 2.2** | 11-21 | 4.69 | 11.4 | 42.9 | 45.7 | 2.1 | 0.2 | 10.7 | 0.0 | 6.8 | 0.6 | 5.3 | 0.1 | 12.8 | 15.2 |
| **Core 2.3** | 21-36 | 4.57 | 10.4 | 44.1 | 45.5 | 1.3 | 0.1 | 10.1 | 0.0 | 6.2 | 0.5 | 5.1 | 0.0 | 11.9 | 10.8 |
| **Core 2.4** | 36-42 | 4.55 | 11.1 | 48.5 | 40.4 | 1.3 | 0.1 | 10.6 | 0.0 | 6.3 | 0.4 | 5.6 | 0.0 | 12.3 | 10.0 |
| **Core 2.5** | 42-57 | 4.47 | 9.3 | 40.6 | 50.2 | 1.0 | 0.1 | 10.1 | 0.0 | 5.6 | 0.3 | 5.3 | 0.0 | 11.3 | 9.2 |
| **Core 2.6** | 57-65 | 4.44 | 8.2 | 42.6 | 49.2 | 0.8 | 0.1 | 9.4 | 0.0 | 5.0 | 0.2 | 4.9 | 0.1 | 10.3 | 8.0 |
| **Core 2.7** | 65-78 | 4.51 | 13.4 | 54.5 | 32.2 | 1.0 | 0.1 | 10.3 | 0.0 | 5.5 | 0.2 | 5.3 | 0.0 | 11.0 | 9.0 |
| **Core 3.1** | 0-11 | 4.98 | 27.9 | 56.2 | 15.9 | 2.7 | 0.2 | 11.0 | 0.0 | 6.8 | 0.3 | 11.1 | 0.1 | 18.2 | 20.0 |
| **Core 3.2** | 11-20 | 4.89 | 30.5 | 55.8 | 13.7 | 2.4 | 0.2 | 11.8 | 0.0 | 6.1 | 0.2 | 10.7 | 0.1 | 17.1 | 19.8 |
| **Core 3.3** | 20-31 | 4.87 | 26.8 | 54.2 | 19.0 | 2.1 | 0.2 | 11.9 | 0.0 | 5.9 | 0.2 | 11.2 | 0.1 | 17.4 | 17.5 |
| **Core 3.4** | 31-41 | 4.84 | 21.0 | 42.6 | 36.4 | 1.7 | 0.1 | 11.8 | 0.0 | 5.8 | 0.2 | 13.1 | 0.1 | 19.3 | 14.9 |
| **Core 3.5** | 41-50 | 4.8 | 33.5 | 60.0 | 6.5 | 1.7 | 0.2 | 11.2 | 0.0 | 5.8 | 0.2 | 15.4 | 0.1 | 21.5 | 14.7 |
| **Core 3.6** | 50-61 | 4.85 | 31.8 | 62.1 | 6.1 | 1.4 | 0.1 | 11.3 | 0.0 | 5.8 | 0.2 | 18.2 | 0.2 | 24.5 | 11.9 |
| **Core 3.7** | 61-70 | 5.05 | 26.4 | 54.8 | 18.9 | 1.3 | 0.1 | 11.3 | 0.0 | 6.0 | 0.3 | 21.1 | 0.2 | 27.5 | 10.5 |
| **Core 3.8** | 70-80 | 5.25 | 28.4 | 57.6 | 14.0 | 1.2 | 0.1 | 9.0 | 0.0 | 5.9 | 0.2 | 21.9 | 0.3 | 28.3 | 9.9 |

Soil pH was measured in 1 M KCl extract.

Clay, silt, and sand represent the particle size distribution of the mineral fraction.

Exchangeable elements were extracted using a cobalt hexamine extraction. The CEC sum (sum) represents the sum of all extracted cations but does not include H^+^.


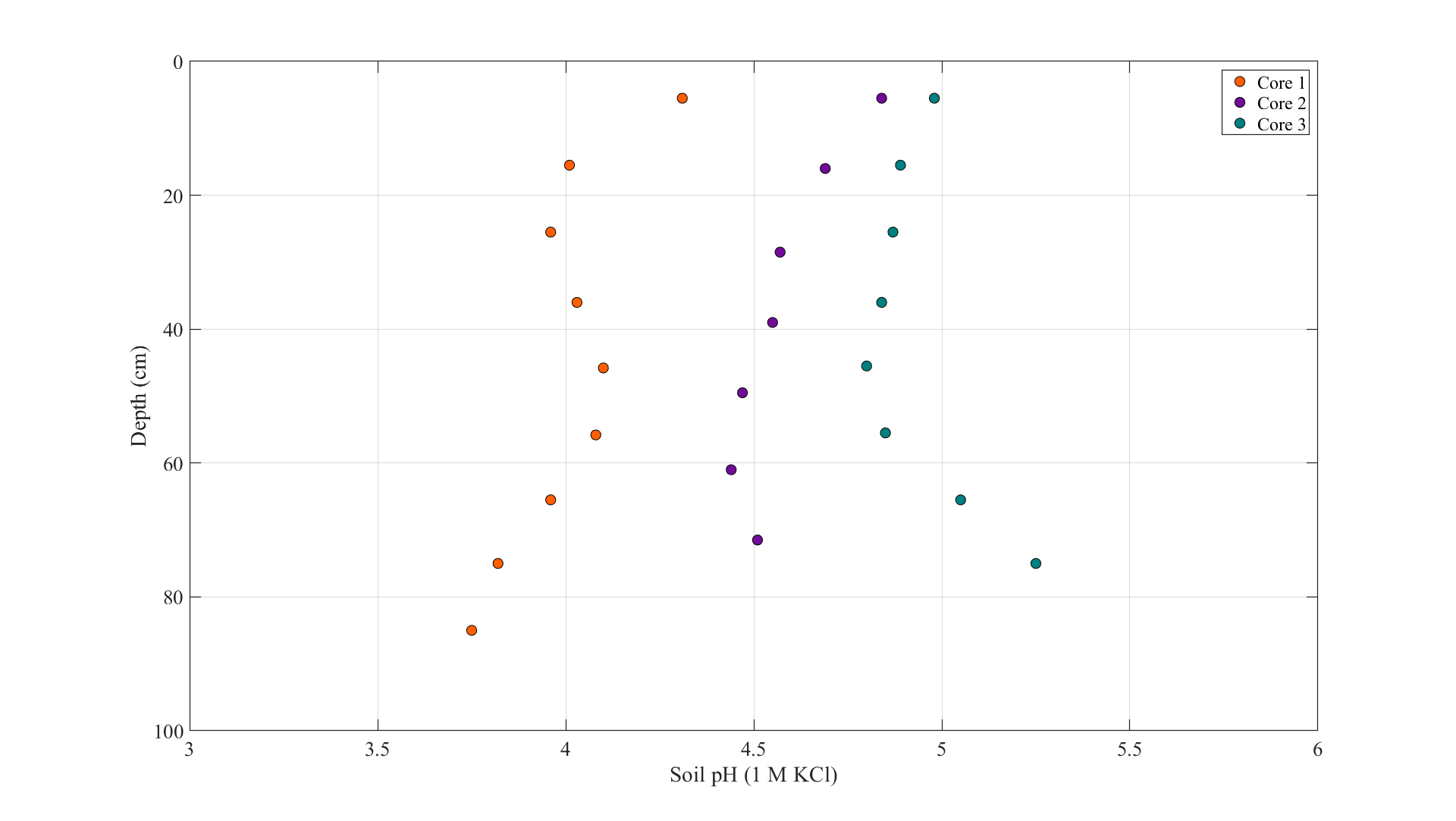


**Fig. S3.** Soil pH of the different soil core samples taken from Point Reyes, California.

**Table S5.** X-ray diffraction analysis attained mineral composition of the 3 depth intervals of each core. Compositions are semi-quantitative and are in relative %.

| **Sample** | **Phyllo**  **silicates** | **Quartz** | **Zeolite** | **Feldspars** | **Feldspathoids** | **Neosilicates** | **Sorosilicates** | **Inosilicate** | **Oxides** | **Sulphates and Sulphides** | **Phosphates** | **Carbonates** | **Arsenates** | **Total** |
| --- | --- | --- | --- | --- | --- | --- | --- | --- | --- | --- | --- | --- | --- | --- |
| **Core 1.1**  0-11 cm | 2 | 20 | 3 | 23 | 5 | 0 | 0 | 19 | 3 | 4 | 21 | 0 | 0 | 100 |
| **Core 1.4**  31-41 cm | 0 | 17 | 0 | 35 | 3 | 0 | 0 | 7 | 2 | 35 | 0 | 0 | 0 | 99 |
| **Core 1.7**  61-70 cm | 9 | 8 | 2 | 35 | 3 | 3 | 0 | 9 | 1 | 13 | 16 | 0 | 0 | 99 |
| **Core 2.1**  0-11 cm | 10 | 13 | 0 | 37 | 14 | 4 | 0 | 0 | 0 | 0 | 22 | 0 | 0 | 100 |
| **Core 2.4**  36-42 cm | 27 | 12 | 0 | 27 | 5 | 1 | 0 | 16 | 0 | 0 | 13 | 0 | 0 | 101 |
| **Core 2.7**  65-78 cm | 5 | 22 | 0 | 0 | 8 | 0 | 8 | 36 | 1 | 3 | 0 | 0 | 17 | 100 |
| **Core 3.1**  0-11 cm | 11 | 38 | 0 | 50 | 0 | 0 | 0 | 0 | 2 | 0 | 0 | 0 | 0 | 101 |
| **Core 3.4**  31-41 cm | 29 | 26 | 0 | 27 | 4 | 2 | 9 | 0 | 0 | 0 | 0 | 2 | 0 | 99 |
| **Core 3.7**  61-70 cm | 21 | 19 | 0 | 21 | 5 | 0 | 0 | 27 | 1 | 7 | 0 | 0 | 0 | 101 |

The compositions above are semi-quantitative and were calculated using a rapid Reference Intensity Ratio method in Highscore.

**Table S6.** Inductively coupled plasma atomic emission spectrometry analysis of major (total rock) elements from the three depth intervals of each soil core (% of oven dried soil).

| **Sample** | **Depth** | **Al** | **Ba** | **Ca** | **Cr** | **Fe** | **K** | **Mg** | **Mn** | **Na** | **P** | **Si** | **Sr** | **Ti** |
| --- | --- | --- | --- | --- | --- | --- | --- | --- | --- | --- | --- | --- | --- | --- |
|  | **cm** | **wt-%** | **wt-%** | **wt-%** | **wt-%** | **wt-%** | **wt-%** | **wt-%** | **wt-%** | **wt-%** | **wt-%** | **wt-%** | **wt-%** | **wt-%** |
| **Core 1.1**  0-11 cm | 0-11 | 6.11 | 0.06 | 0.59 | 0.04 | 2.35 | 1.39 | 0.60 | 0.04 | 1.54 | 0.07 | 37.10 | 0.02 | 0.30 |
| **Core 1.4**  31-41 cm | 31-41 | 6.29 | 0.07 | 0.57 | 0.04 | 2.46 | 1.35 | 0.60 | 0.04 | 1.52 | 0.08 | 37.21 | 0.02 | 0.31 |
| **Core 1.7**  61-70 cm | 61-70 | 6.07 | 0.06 | 0.53 | 0.04 | 2.28 | 1.33 | 0.63 | 0.02 | 1.59 | 0.03 | 37.18 | 0.01 | 0.30 |
| **Core 2.1**  0-11 cm | 0-11 | 7.15 | 0.05 | 0.92 | 0.03 | 3.47 | 1.28 | 1.58 | 0.07 | 2.22 | 0.07 | 33.86 | 0.02 | 0.39 |
| **Core 2.4**  36-42 cm | 36-42 | 7.22 | 0.05 | 0.81 | 0.03 | 3.57 | 1.21 | 1.70 | 0.07 | 2.23 | 0.05 | 33.79 | 0.02 | 0.41 |
| **Core 2.7**  65-78 cm | 65-78 | 6.95 | 0.05 | 0.76 | 0.03 | 3.24 | 1.11 | 1.51 | 0.06 | 2.33 | 0.04 | 34.34 | 0.02 | 0.37 |
| **Core 3.1**  0-11 cm | 0-11 | 5.81 | 0.04 | 0.66 | 0.06 | 5.16 | 0.87 | 1.36 | 0.12 | 1.43 | 0.06 | 34.86 | 0.01 | 0.58 |
| **Core 3.4**  31-41 cm | 31-41 | 6.05 | 0.04 | 0.64 | 0.07 | 5.32 | 0.83 | 1.56 | 0.12 | 1.39 | 0.04 | 34.82 | 0.01 | 0.57 |
| **Core 3.7**  61-70 cm | 61-70 | 6.80 | 0.05 | 0.63 | 0.07 | 6.38 | 0.90 | 2.23 | 0.15 | 1.31 | 0.03 | 32.34 | 0.01 | 0.59 |

**Table S7.** Inductively coupled plasma mass spectrometry analysis of trace elements from the three depth intervals of each soil core (parts per million of oven dried soil).

| **Sample** | **Ce** | **Cs** | **Dy** | **Er** | **Eu** | **Ga** | **Gd** | **Hf** | **Ho** | **La** | **Lu** | **Nb** | **Nd** | **Pr** |
| --- | --- | --- | --- | --- | --- | --- | --- | --- | --- | --- | --- | --- | --- | --- |
|  | **ppm** | **ppm** | **ppm** | **ppm** | **ppm** | **ppm** | **ppm** | **ppm** | **ppm** | **ppm** | **ppm** | **ppm** | **ppm** | **ppm** |
| **Core 1.1**  0-11 cm | 26.5 | 2.5 | 2.1 | 1.1 | 0.5 | 13.2 | 2.0 | 7.3 | 0.4 | 14.0 | 0.2 | 6.3 | 11.8 | 3.0 |
| **Core 1.4**  31-41 cm | 27.3 | 2.6 | 2.0 | 1.4 | 0.5 | 14.0 | 2.2 | 6.3 | 0.5 | 15.0 | 0.2 | 6.1 | 12.9 | 3.3 |
| **Core 1.7**  61-70 cm | 21.9 | 2.4 | 1.7 | 1.2 | 0.4 | 12.4 | 1.5 | 6.5 | 0.3 | 11.3 | 0.2 | 6.6 | 9.6 | 2.5 |
| **Core 2.1**  0-11 cm | 35.8 | 2.5 | 3.1 | 2.1 | 0.8 | 15.3 | 3.4 | 5.2 | 0.6 | 17.6 | 0.3 | 8.8 | 18.3 | 4.2 |
| **Core 2.4**  36-42 cm | 36.0 | 2.4 | 3.4 | 1.9 | 0.8 | 15.7 | 3.3 | 5.6 | 0.6 | 17.7 | 0.2 | 9.2 | 18.0 | 4.3 |
| **Core 2.7**  65-78 cm | 34.8 | 2.4 | 3.0 | 1.8 | 0.8 | 15.3 | 3.1 | 4.7 | 0.6 | 17.4 | 0.2 | 8.6 | 16.3 | 4.1 |
| **Core 3.1**  0-11 cm | 31.9 | 2.0 | 4.7 | 2.6 | 1.0 | 14.6 | 4.5 | 3.7 | 0.9 | 15.7 | 0.3 | 7.4 | 19.2 | 4.6 |
| **Core 3.4**  31-41 cm | 31.0 | 2.0 | 4.6 | 2.7 | 1.2 | 15.3 | 4.8 | 3.2 | 0.9 | 16.2 | 0.3 | 7.1 | 19.3 | 4.5 |
| **Core 3.7**  61-70 cm | 33.8 | 2.7 | 4.9 | 2.8 | 1.2 | 17.4 | 4.6 | 3.2 | 0.9 | 15.8 | 0.3 | 7.6 | 19.2 | 4.6 |
| **Sample** | **Rb** | **Sm** | **Sn** | **Sr** | **Ta** | **Tb** | **Th** | **Tm** | **U** | **V** | **W** | **Y** | **Yb** | **Zr** |
|  | **ppm** | **ppm** | **ppm** | **ppm** | **ppm** | **ppm** | **ppm** | **ppm** | **ppm** | **ppm** | **ppm** | **ppm** | **ppm** | **ppm** |
| **Core 1.1**  0-11 cm | 76.3 | 2.3 | 1.1 | 137.6 | 0.6 | 0.3 | 4.0 | 0.2 | 2.0 | 71.6 | 63.7 | 11.1 | 1.3 | 287.7 |
| **Core 1.4**  31-41 cm | 73.9 | 2.4 | 1.1 | 137.1 | 0.4 | 0.3 | 4.4 | 0.2 | 2.0 | 77.7 | 27.8 | 12.3 | 1.3 | 247.5 |
| **Core 1.7**  61-70 cm | 58.6 | 2.0 | 1.1 | 126.0 | 0.4 | 0.2 | 4.1 | 0.1 | 2.0 | 74.7 | 13.9 | 9.7 | 1.3 | 257.3 |
| **Core 2.1**  0-11 cm | 52.0 | 3.8 | 3.3 | 146.8 | 0.7 | 0.5 | 5.1 | 0.3 | 1.9 | 104.9 | 2.2 | 16.6 | 1.8 | 205.4 |
| **Core 2.4**  36-42 cm | 51.7 | 3.8 | 2.1 | 131.0 | 0.6 | 0.5 | 5.8 | 0.3 | 1.9 | 106.3 | 62.3 | 16.5 | 1.9 | 226.6 |
| **Core 2.7**  65-78 cm | 47.7 | 3.1 | 2.1 | 128.6 | 0.6 | 0.4 | 5.5 | 0.2 | 1.7 | 97.8 | 14.9 | 15.2 | 1.7 | 174.3 |
| **Core 3.1**  0-11 cm | 44.4 | 4.7 | 1.1 | 61.8 | 0.4 | 0.7 | 3.8 | 0.3 | 1.5 | 147.2 | 41.2 | 23.7 | 2.5 | 138.2 |
| **Core 3.4**  31-41 cm | 42.3 | 4.9 | 1.1 | 59.2 | 0.4 | 0.7 | 3.8 | 0.4 | 1.4 | 141.7 | 24.2 | 24.0 | 2.5 | 116.5 |
| **Core 3.7**  61-70 cm | 45.5 | 4.6 | 1.1 | 53.6 | 0.6 | 0.7 | 4.3 | 0.3 | 1.5 | 166.9 | 22.2 | 23.9 | 2.7 | 115.7 |

**Table S8.** Semi-quantification of the proportions of calcium containing minerals in our bulk soil samples, reported as % of the total mineral content of each sample.

| **Sample** | **Rondorfite** | **Rankinite** | **Epidote** | **Diopside** | **Kupletskite** | **Wollastonite** | **Montmorillonite** |
| --- | --- | --- | --- | --- | --- | --- | --- |
|  | **Ca64.00 Mg8.00 Si32.00 O128.00 Cl16.00** | **Ca12.00 Si8.00 O28.00** | **Ca4.00 Al4.00 Fe2.00 Si6.00 O26.00** | **Si8.00 Mg4.00 Ca4.00 O24.00** | **Mn24.68 Na1.90 Mg0.28 Ti7.05 Nb0.95 Si32.00 K4.00 Ca0.80 O120.00 F2.00** | **Ca5.76 Mn0.24 Si6.00 O18.00** | **Al4.00 Si8.00 O24.00 Ca1.00** |
| **Core 1.1**  0-11 cm |  |  |  |  | 1 |  |  |
| **Core 1.4**  31-41 cm |  |  |  |  |  |  |  |
| **Core 1.7**  61-70 cm |  |  |  |  |  |  |  |
| **Core 2.1**  0-11 cm |  |  |  |  |  |  | 1 |
| **Core 2.4**  36-42 cm |  |  |  | 2 |  | 5 |  |
| **Core 2.7**  65-78 cm |  |  | 8 |  |  | 16 | 3 |
| **Core 3.1**  0-11 cm |  |  |  |  |  |  |  |
| **Core 3.4**  31-41 cm | 2 | 9 |  |  |  |  |  |
| **Core 3.7**  61-70 cm |  |  |  | 4 |  |  |  |

| **Sample** | **Phillipsite-Ca** | **Anorthite** | **Cancrinite** | **Lazurite** | **Pyrochlore** | **Ankerite** | **Dolomite** |
| --- | --- | --- | --- | --- | --- | --- | --- |
|  | **K2.00 Ca1.64 Al5.34 Si10.66 O32.00 At12.00** | **Ca8.00 Si16.00 Al16.00 O64.00** | **Na6.02 Ca1.50 Al6.00 Si6.00 C1.44 O28.32** | **Na39.08 Ca8.00 K0.68 Si37.00 Al35.00 S11.52 O177.28 Cl0.72** | **Nb8.00 Ca8.00 O96.00 F16.00** | **Ca2.99 Mg0.82 Fe2.03 Mn0.16 C6.00 O18.00** | **Ca3.00 Mg3.00 C6.00 O18.00** |
| **Core 1.1**  0-11 cm |  |  |  | 5 |  |  |  |
| **Core 1.4**  31-41 cm |  | 16 |  | 3 |  |  |  |
| **Core 1.7**  61-70 cm | 2 | 13 |  | 3 |  |  |  |
| **Core 2.1**  0-11 cm |  | 12 | 3 | 9 |  |  |  |
| **Core 2.4**  36-42 cm |  | 13 |  | 4 |  |  |  |
| **Core 2.7**  65-78 cm |  |  |  | 8 | 1 |  |  |
| **Core 3.1**  0-11 cm |  | 20 |  |  |  |  |  |
| **Core 3.4**  31-41 cm |  | 14 |  | 4 |  | < 1 | < 1 |
| **Core 3.7**  61-70 cm |  |  |  | 5 |  |  |  |

The compositions above are semi-quantitative and were calculated using a rapid Reference Intensity Ratio method in Highscore.

**Table S9.** Principal component analysis on the bulk soil characterisation data statistics.

| **Simple Statistics** | | | | | | | | |
| --- | --- | --- | --- | --- | --- | --- | --- | --- |
|  | **Soil pH** | **SOC** | **Clay** | **Na_Exch_** | **Mg_Exch_** | **Al_Exch_** | **K_Exch_** | **Ca_Exch_** |
| **Mean** | 4.48 | 1.93 | 19.73 | 0.11 | 7.87 | 0.31 | 0.29 | 6.29 |
| **StD** | 0.43 | 0.97 | 7.77 | 0.09 | 6.06 | 0.45 | 0.20 | 0.80 |

| **Correlation Matrix** | | | | | | | | | |
| --- | --- | --- | --- | --- | --- | --- | --- | --- | --- |
|  | **Soil pH** | **SOC** | **Clay** | **Na_Exch_** | **Mg_Exch_** | **Al_Exch_** | **K_Exch_** | **Ca_Exch_** |  |
| **Soil pH** |  | -0.07 | 0.38 | 0.32 | 0.80 | -0.89 | 0.19 | -0.08 |  |
| **SOC** | -0.07 |  | 0.12 | -0.18 | -0.30 | 0.21 | 0.59 | 0.69 |  |
| **Clay** | 0.38 | 0.12 |  | 0.31 | 0.65 | -0.07 | -0.35 | -0.07 |  |
| **Na_Exch_** | 0.32 | -0.18 | 0.31 |  | 0.53 | -0.10 | -0.18 | 0.00 |  |
| **Mg_Exch_** | 0.80 | -0.30 | 0.65 | 0.53 |  | -0.57 | -0.17 | -0.25 |  |
| **Al_Exch_** | -0.89 | 0.21 | -0.07 | -0.10 | -0.57 |  | -0.29 | 0.18 |  |
| **K_Exch_** | 0.19 | 0.59 | -0.35 | -0.18 | -0.17 | -0.29 |  | 0.57 |  |
| **Ca_Exch_** | -0.08 | 0.69 | -0.07 | 0.00 | -0.25 | 0.18 | 0.57 |  |  |

**Table S10.** Eigenvalues and vectors from the principal component analysis of the bulk characterisation data presented in Fig. 2D.

| **Eigenvalues of the Correlation Matrix** | | | | |
| --- | --- | --- | --- | --- |
|  | **Eigenvalue** | **Difference** | **Proportion** | **Cumulative** |
| **1** | 3.13 | 0.97 | 0.39 | 0.39 |
| **2** | 2.16 | 0.79 | 0.27 | 0.66 |
| **3** | 1.38 | 0.60 | 0.17 | 0.83 |
| **4** | 0.78 | 0.50 | 0.10 | 0.93 |
| **5** | 0.28 | 0.10 | 0.03 | 0.97 |
| **6** | 0.17 | 0.10 | 0.02 | 0.99 |
| **7** | 0.08 | 0.06 | 0.01 | 1.00 |
| **8** | 0.01 |  | 0.00 | 1.00 |

| **Eigenvectors** | | | | | | | | |
| --- | --- | --- | --- | --- | --- | --- | --- | --- |
|  | **Prin1** | **Prin2** | **Prin3** | **Prin4** | **Prin5** | **Prin6** | **Prin7** | **Prin8** |
| **Soil pH** | 0.47 | 0.34 | -0.16 | -0.08 | -0.06 | -0.28 | 0.35 | 0.66 |
| **SOC** | -0.26 | 0.48 | 0.33 | -0.26 | 0.46 | -0.34 | 0.33 | -0.29 |
| **Clay** | 0.32 | 0.03 | 0.58 | -0.49 | 0.00 | 0.09 | -0.54 | 0.16 |
| **Na_Exch_** | 0.30 | 0.01 | 0.38 | 0.78 | 0.31 | -0.18 | -0.15 | -0.01 |
| **Mg_Exch_** | 0.53 | 0.10 | 0.14 | -0.02 | -0.10 | 0.54 | 0.46 | -0.42 |
| **Al_Exch_** | -0.39 | -0.30 | 0.47 | 0.06 | 0.08 | 0.36 | 0.41 | 0.49 |
| **K_Exch_** | -0.16 | 0.58 | -0.25 | 0.13 | 0.32 | 0.58 | -0.29 | 0.20 |
| **Ca_Exch_** | -0.25 | 0.47 | 0.30 | 0.23 | -0.75 | -0.05 | -0.03 | -0.04 |

**Table S11.** Target transform results for the standard spectra (Table S1) over the principal component analysis of our bulk XANES spectra (4 principal components) and μ-XANES spectra (5 principal components) over an energy interval of 4030 – 4070 eV. The following statistics are presented, SPOIL values, R Factor, residual sum of square and chi-squared statistics in SIXPACK (V0.68) and Athena (RSS computation). SPOIL Values were higher for our µ-XANES data. According to Malinowski (1978) an excellent SPOIL value ranges between 0-1.5, good values 1.5-3, which are all acceptable, a fair value ranges between 3-4.5, poor values 4.5-6, which are moderately acceptable, and anything with a value > 6 is not acceptable. .

| **Medium energy interval 40 eV (4030 - 4070 eV)** | | | | | | | | |
| --- | --- | --- | --- | --- | --- | --- | --- | --- |
| **Standard** | **Bulk XANES (principal components 1:4)** | | | | **µ-XANES (principal components 1:5)** | | | |
|  | **R** | **RSS** | ***χ*^2^** | **Spoil** | **R** | **RSS** | ***χ*^2^** | **SPOIL** |
| **Solvated Ca** | 0.00081 | 0.1878925 | 0.37889 | 2.13 | 0.00067 | 0.16211502 | 0.3135 | 2.80 |
| **ACC** | 0.00132 | 0.3024314 | 0.58371 | 2.46 | 0.00075 | 0.17528961 | 0.33058 | 3.59 |
| **Gypsum** | 0.00159 | 0.3363671 | 0.69064 | 3.01 | 0.00066 | 0.14841351 | 0.28868 | 2.85 |
| **Apatite** | 0.00273 | 0.4780033 | 0.93558 | 3.76 | 0.00259 | 0.48632744 | 0.89062 | 5.42 |
| **Monetite** | 0.00185 | 0.408607 | 0.85749 | 3.16 | 0.00135 | 0.30408913 | 0.62654 | 7.00 |
| **Brushite** | 0.00388 | 0.8048628 | 1.62959 | 2.83 | 0.00192 | 0.4098049 | 0.80674 | 4.88 |
| **Wollastonite** | 0.00162 | 0.3045071 | 0.61969 | 1.69 | 0.00142 | 0.27406685 | 0.54287 | 4.34 |
| **Anorthite** | 0.00247 | 0.5440521 | 1.04462 | 1.86 | 0.00169 | 0.36491196 | 0.71573 | 6.19 |
| **Californian montmorillonite** | 0.00131 | 0.2694114 | 0.56445 | 2.78 | 0.00117 | 0.21960418 | 0.50773 | 10.12 |
| **Ca exchanged Montmorillonite** | 0.0007 | 0.1585555 | 0.31132 | 2.25 | 0.00027 | 0.05097084 | 0.12292 | 3.37 |
| **Ca exchanged ferrihydrite** | 0.00031 | 0.0681673 | 0.1319 | 4.28 | 0.0001 | 0.0278331 | 0.0606 | 3.22 |
| **Fe Mn nodule Pt. Reyes** | 0.0006 | 0.1206455 | 0.25922 | 1.73 | 0.00062 | 0.10672179 | 0.27071 | 6.09 |
| **Ca benzoate** | 0.00088 | 0.210305 | 0.41467 | 1.66 | 0.00031 | 0.05859818 | 0.14923 | 2.04 |
| **Ca citrate** | 0.00124 | 0.2771812 | 0.55329 | 2.53 | 0.00076 | 0.14935743 | 0.33966 | 3.32 |
| **Ca phytic** | 0.00061 | 0.1412701 | 0.28362 | 2.04 | 0.00033 | 0.05842012 | 0.15421 | 2.67 |
| **Ca SRHA** | 0.00142 | 0.3009674 | 0.61154 | 4.22 | 0.0015 | 0.28404961 | 0.6464 | 7.30 |
| **Ca oxalate** | 0.00105 | 0.2139919 | 0.48511 | 1.22 | 0.00091 | 0.23729484 | 0.41854 | 2.81 |
| **Pt. Reyes litter** | 0.00031 | 0.070638 | 0.13524 | 2.40 | 0.0002 | 0.03385448 | 0.0865 | 3.09 |


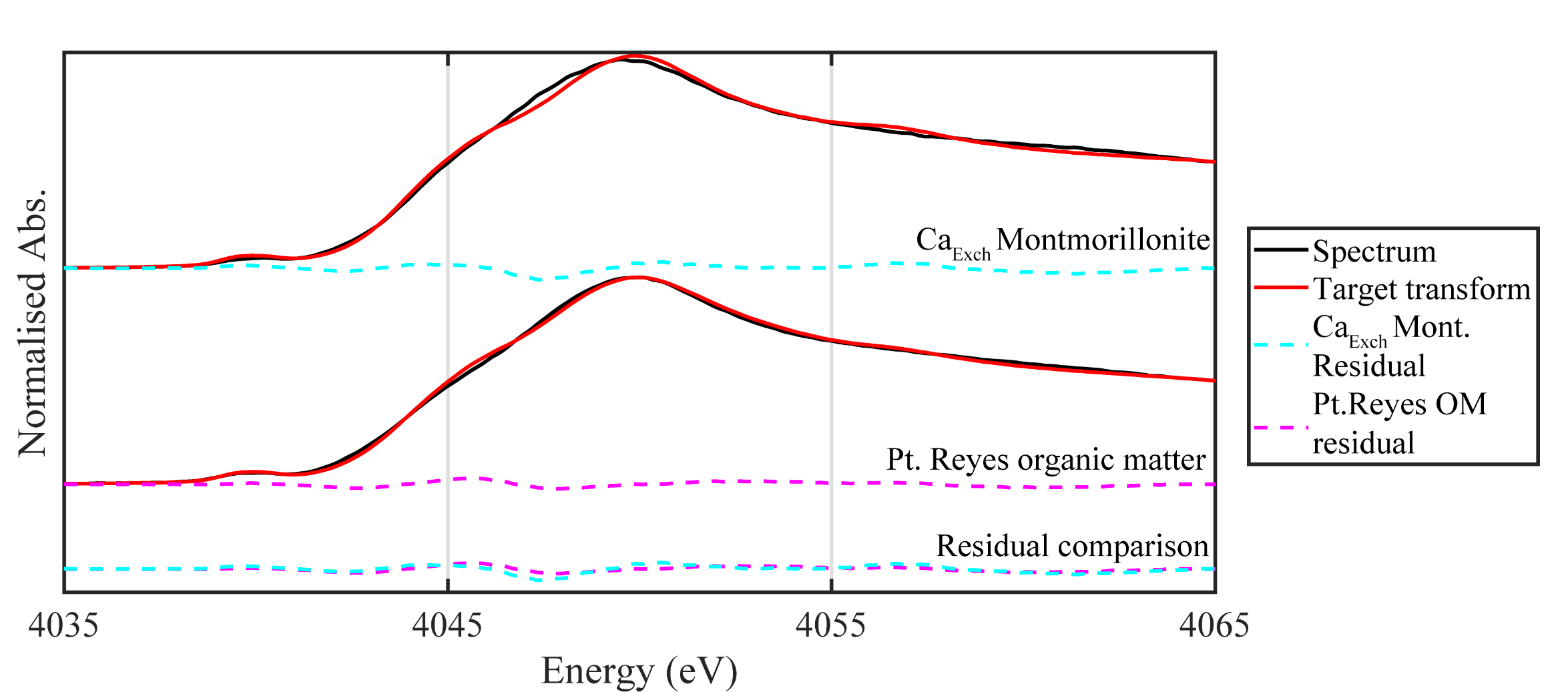


**Fig. S4.** Although calcium exchanged montmorillonite had a slightly lower SPOIL value, the target transform analysis also indicated that it had a higher residual than for the Pt. Reyes organic matter sample, which was identified in small quantities in only two samples on the XRD. We thus, excluded Ca exchanged montmorillonite from our final standard subset.

**Table S12.** Target transform analysis of our µ-XANES data that clearly display the 4055 eV feature using the first 4 principal components of our principal component analysis of the bulk Ca K-edge XANES sample spectra. The SPOIL values of this target transform analysis demonstrated that Core 1.1 µ-XANES spectra 2. gave the best reconstruction of the variation in our bulk data. We thus included Core 1.1 µ-XANES spectra 2 in our linear combination fitting analysis.

| **µ-XANES spectra that display a clear 4055 eV feature** | **SPOIL value**  **Standard recreation** |
| --- | --- |
| **1.1.2** | 0.4001 |
| **2.1.6** | 1.4651 |
| **2.4.2** | 5.1163 |
| **3.4.1** | 5.2341 |
| **3.7.1** | 4.54 |
| **3.7.6** | 5.505 |
| **3.7.7** | 5.9176 |

**Table S13.** Linear combination fitting model results from Athena for 6 different model sets, detailed in the list below the table. Included statistics are the R factor (sum of squares of the differences between the data and the fit at each data point, divided by the sum of the squares of the data at each corresponding point), the chi-squared and reduced chi-squared values from each linear combination fit of the 3 depth intervals. It was found that model set F containing the Pt.Reyes litter, Ca benzoate, Core 1.1 Core 1.1 µ-XANES spectra 2, and anorthite standards gave the best reconstruction of our bulk data.

| **Standard sets** | **Stat** | **Core 1** | | | **Core 2** | | | **Core 3** | | | |
| --- | --- | --- | --- | --- | --- | --- | --- | --- | --- | --- | --- |
|  |  | **Depth Intervals (cm)** | | | | | | | | | |
|  |  | **0-11** | **31-41** | **61-70** | **0-11** | **36-42** | **65-78** | **0-11** | **31-41** | **61-70** |  |
| **A** | **R factor** | 0.00281 | 0.00360 | 0.00598 | 0.00648 | 0.00356 | 0.01522 | 0.01003 | 0.01013 | 0.01039 |  |
| **B** |  | 0.00054 | 0.00064 | 0.00176 | 0.00047 | 0.00041 | 0.00185 | 0.00036 | 0.00037 | 0.00074 |  |
| **C** |  | 0.00053 | 0.00066 | 0.00176 | 0.00044 | 0.00040 | 0.00183 | 0.00035 | 0.00037 | 0.00072 |  |
| **D** |  | 0.00127 | 0.00158 | 0.00311 | 0.00066 | 0.00051 | 0.00176 | 0.00042 | 0.00059 | 0.00090 |  |
| **E** |  | 0.00051 | 0.00063 | 0.00175 | 0.00045 | 0.00082 | 0.00177 | 0.00041 | 0.00052 | 0.00121 |  |
| **F** |  | 0.00041 | 0.00049 | 0.00093 | 0.00045 | 0.00072 | 0.00126 | 0.00041 | 0.00051 | 0.00116 |  |
| **A** | ***χ*^2^** | 0.19861 | 0.25237 | 0.40751 | 0.44221 | 0.26092 | 0.94723 | 0.66178 | 0.67240 | 0.70617 |  |
| **B** |  | 0.03766 | 0.04473 | 0.11943 | 0.03162 | 0.03037 | 0.11435 | 0.02390 | 0.02433 | 0.05045 |  |
| **C** |  | 0.00019 | 0.00024 | 0.00062 | 0.00016 | 0.00016 | 0.00059 | 0.00012 | 0.00013 | 0.00026 |  |
| **D** |  | 0.08924 | 0.11013 | 0.21102 | 0.04498 | 0.03750 | 0.10896 | 0.02759 | 0.03920 | 0.06110 |  |
| **E** |  | 0.03585 | 0.04410 | 0.11857 | 0.03087 | 0.06030 | 0.10928 | 0.02703 | 0.03460 | 0.08208 |  |
| **F** |  | 0.02846 | 0.03398 | 0.06312 | 0.03058 | 0.04949 | 0.08132 | 0.0266 | 0.03398 | 0.07843 |  |
| **A** | **Reduced *χ*^2^** | 0.00103 | 0.00131 | 0.00212 | 0.00230 | 0.00136 | 0.00493 | 0.00345 | 0.00350 | 0.00368 |  |
| **B** |  | 0.00020 | 0.00023 | 0.00063 | 0.00017 | 0.00016 | 0.00060 | 0.00013 | 0.00013 | 0.00026 |  |
| **C** |  | 0.03740 | 0.04613 | 0.11890 | 0.03006 | 0.03009 | 0.11290 | 0.02311 | 0.02429 | 0.04911 |  |
| **D** |  | 0.00046 | 0.00057 | 0.00109 | 0.00023 | 0.00019 | 0.00056 | 0.00014 | 0.00020 | 0.00032 |  |
| **E** |  | 0.00019 | 0.00023 | 0.00061 | 0.00016 | 0.00031 | 0.00057 | 0.00014 | 0.00018 | 0.00043 |  |
| **F** |  | 0.00015 | 0.00018 | 0.00033 | 0.00016 | 0.00026 | 0.00042 | 0.00014 | 0.00018 | 0.00041 |  |

**Standard sets**

1. 4 standards without μXANES: Pt. Reyes litter, Ca benzoate, Ca oxalate, Solvated Ca.
2. 4 standards with μXANES: Pt. Reyes litter, Ca benzoate, Ca oxalate, Solvated Ca, Core 1.1 uXANES spectra 2.
3. 3 standards with μXANES: Pt. Reyes litter, Ca benzoate, Ca oxalate, Core 1.1 uXANES spectra 2.
4. 2 standards with μXANES: Pt. Reyes litter, Ca oxalate, Core 1.1 uXANES spectra 2.
5. 2 standards with μXANES: Pt. Reyes litter, Ca benzoate, Core 1.1 uXANES spectra 2.
6. **Applied standard set –** 3 standards with μXANES: Pt Reyes litter, Ca benzoate, Core 1.1 uXANES spectra 2, and anorthite.

**Table S14.** As a rough calculation, a separate principal component analysis was completed using the mineral and organic standards and then the first 4 principal components were used to run a target transform analysis individually on each µ-XANES spectrum that clearly displayed the 4055 eV feature. The SPOIL values were nearly always smaller for the organic standard subset.

| **µ-XANES spectra that display a clear 4055 eV feature** | **Mineral standards PCA SPOIL Values** | **Organic standards PCA SPOIL Values** |
| --- | --- | --- |
| **1.1.2** | 1.5337 | 1.6274 |
| **2.1.6** | 1.5047 | 2.8121 |
| **2.4.2** | 2.8989 | 1.6985 |
| **3.4.1** | 2.6128 | 1.6336 |
| **3.7.1** | 1.5173 | 1.2601 |
| **3.7.6** | 2.3469 | 1.4906 |
| **3.7.7** | 2.5946 | 1.5598 |


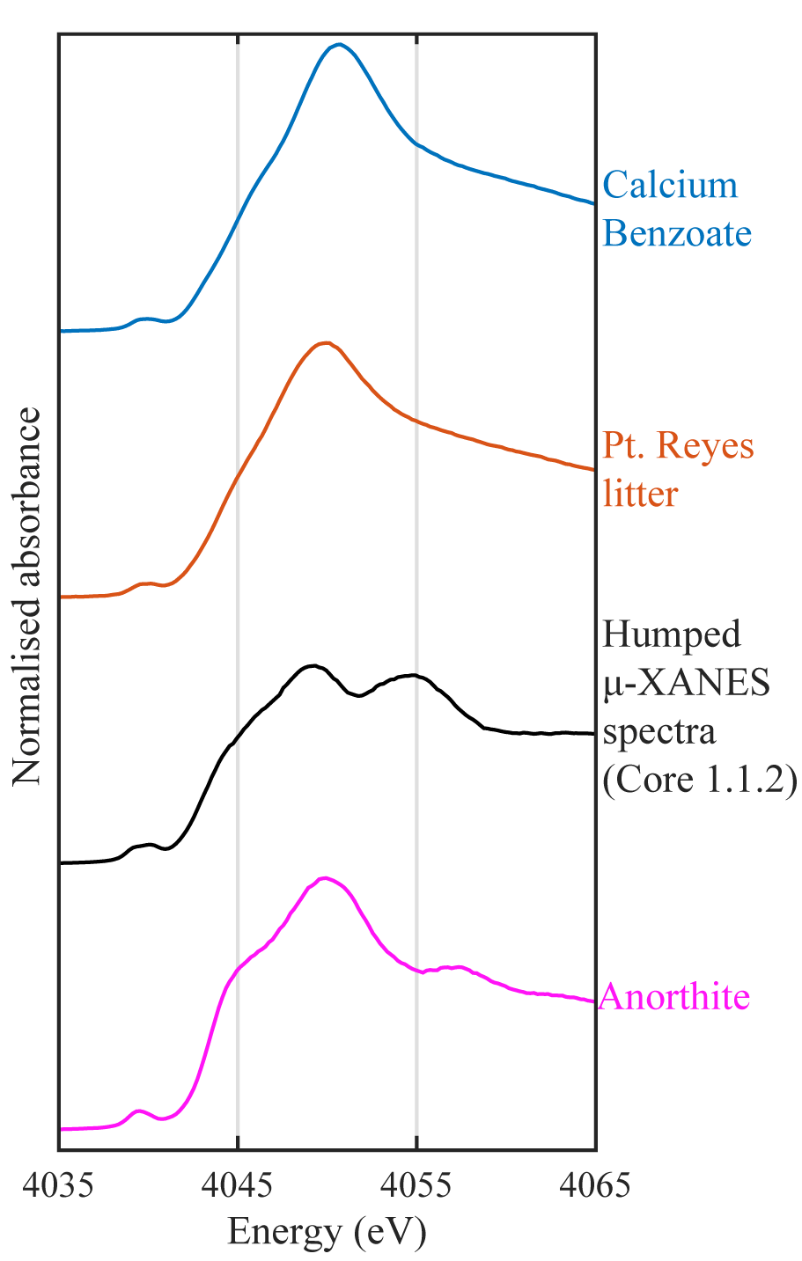


**Fig. S5.** Normalised Ca K-edge XANES spectra of the mineral, organic standards, and µ-XANES spectra used in the linear combination fitting analysis of the bulk XANES sample spectra.


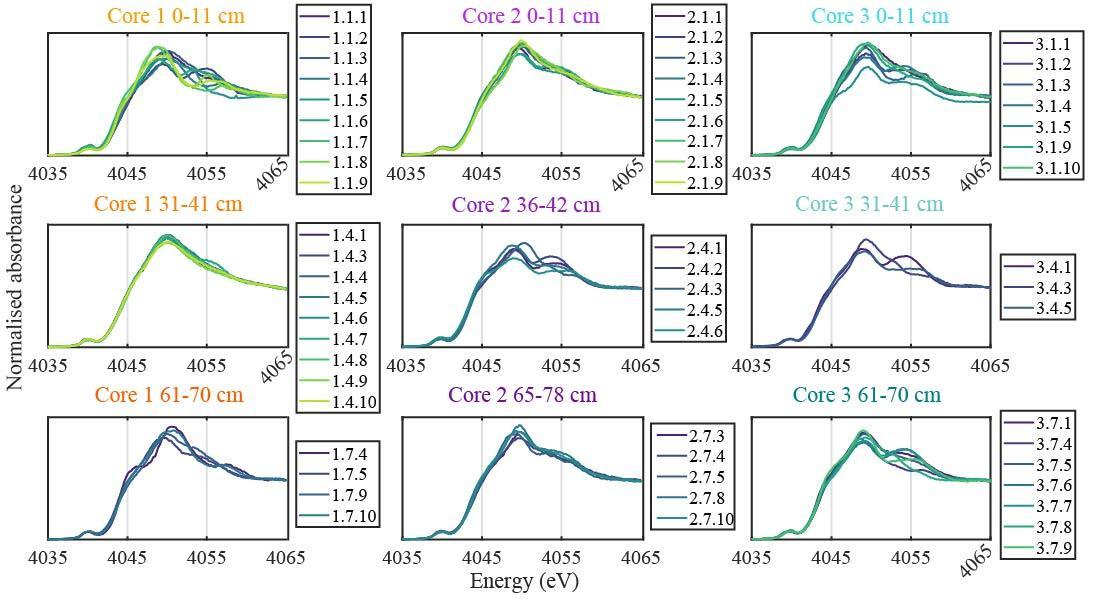


**Fig S6.** Normalised spectra from the μ-XANES analysis selected from the multi-energy maps using simplex volume maximisation analysis on the 3 depth intervals. Sample locations are presented below in Fig. S6-S11.


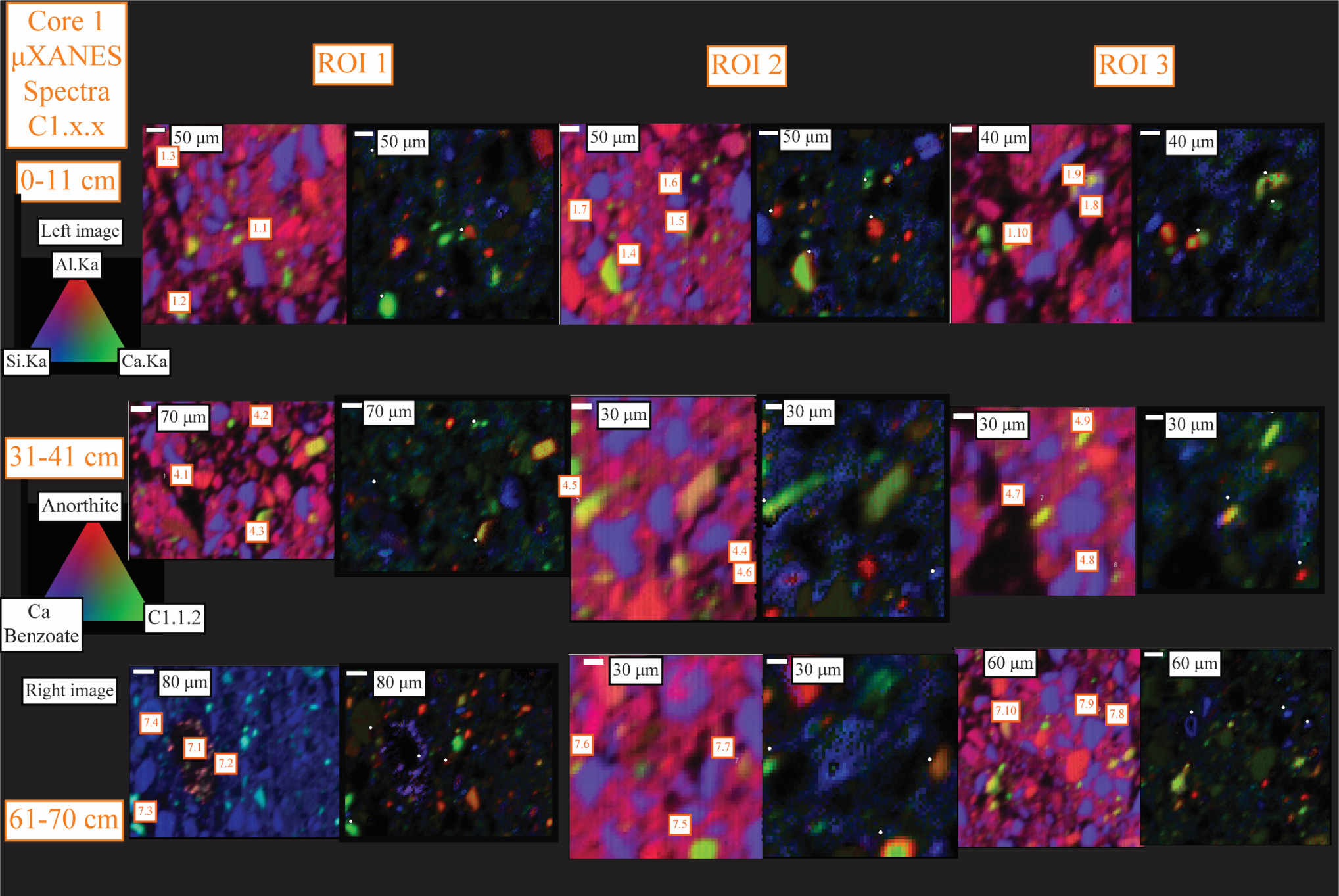


**Fig. S7.** Micro-X-ray fluorescence maps and micro-X-ray absorption near edge structure spectra locations in Core 1. Maps on the left-hand image are tricolour image of the Al (red), Si (blue), and Ca (green) concentration and maps on the right-hand side are the least squares fitted multi-energy maps with the Ca benzoate, anorthite, and µ-XANES standard.


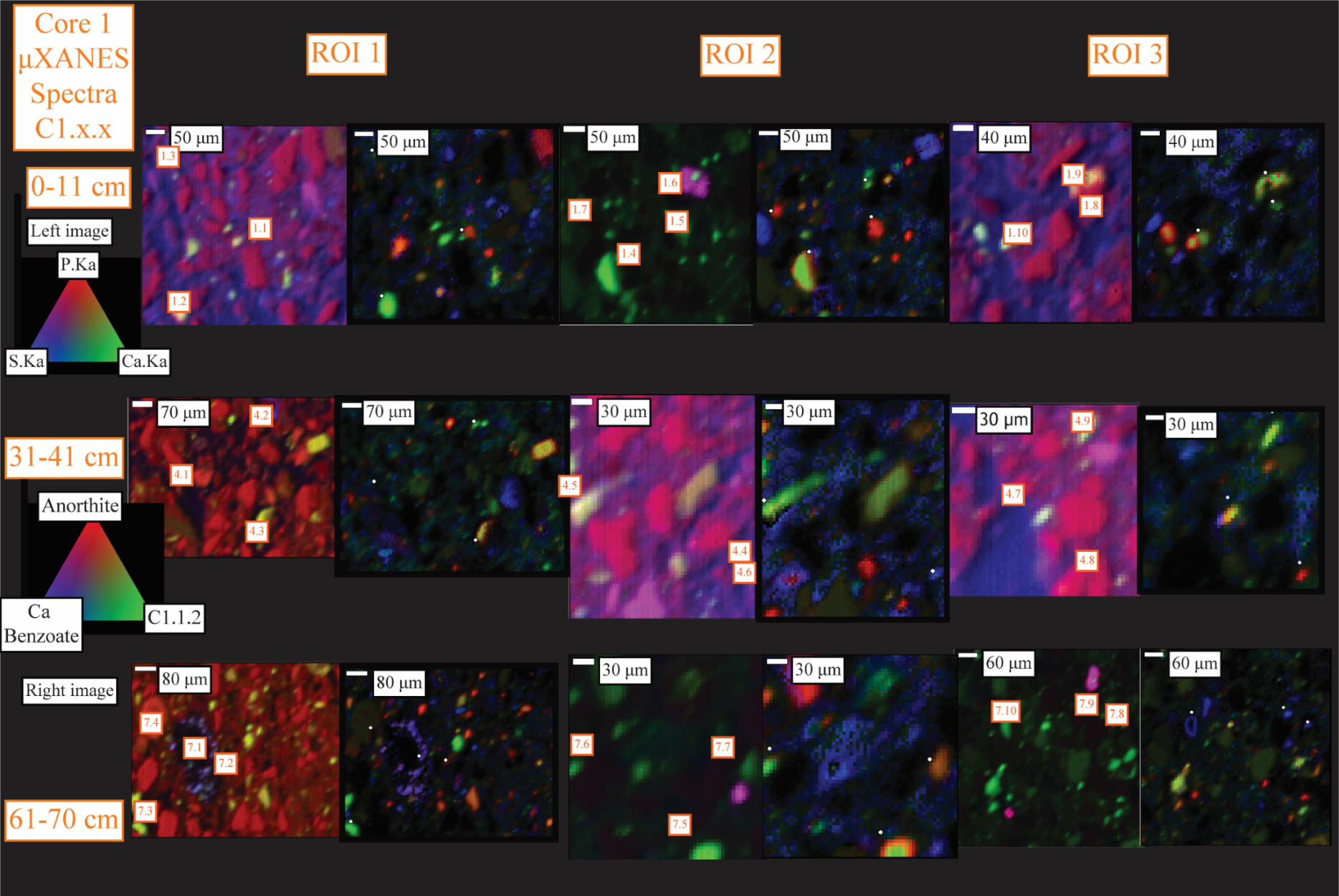


**Fig. S8.** Micro-X-ray fluorescence maps and micro-X-ray absorption near edge structure spectra locations in Core 1. Maps on the left-hand image are tricolour image of the P (red), S (blue), and Ca (green) concentration and maps on the right-hand side are the least squares fitted multi-energy maps with the Ca benzoate, anorthite, and µ-XANES standard.


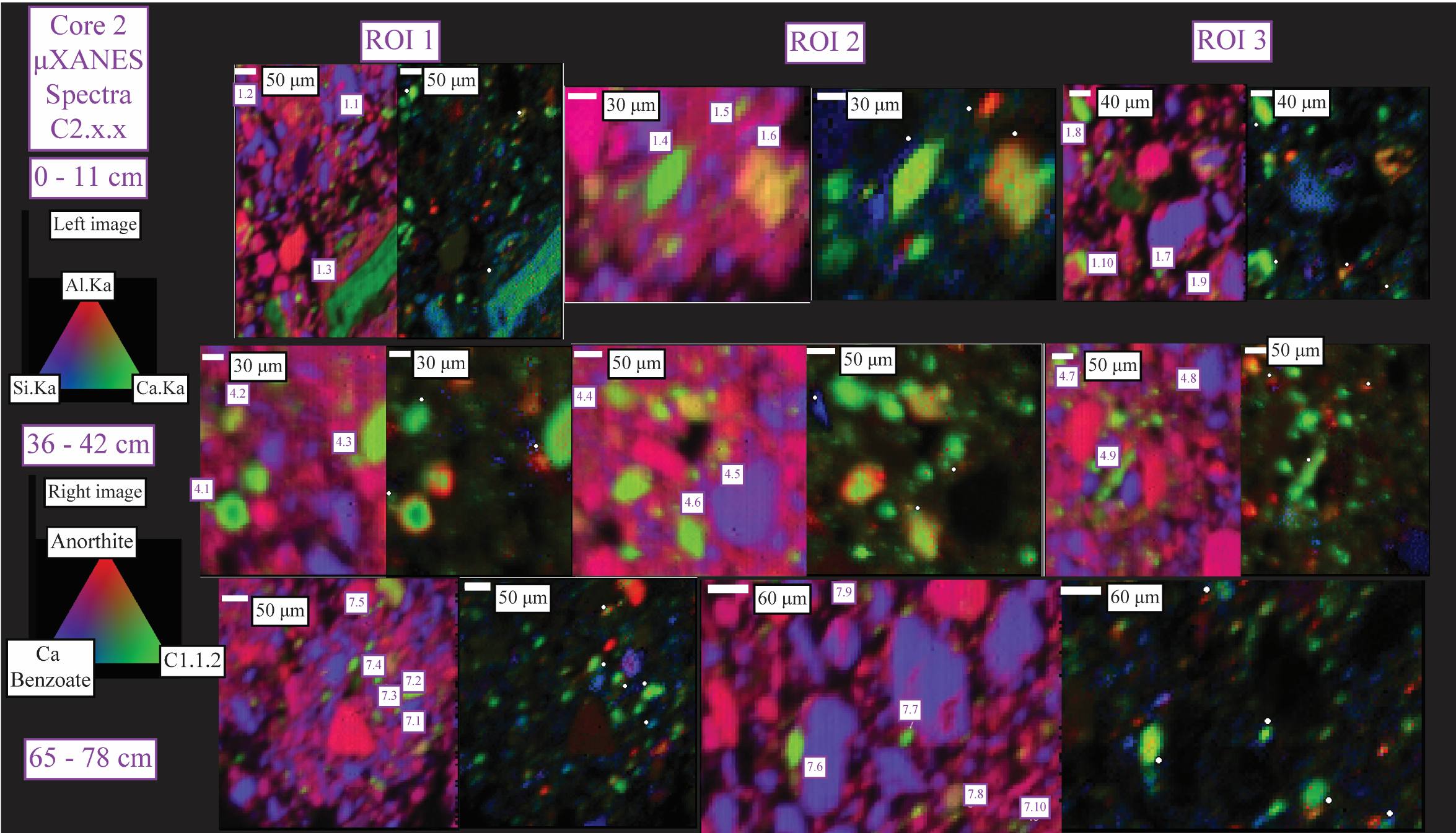
 **Fig. S9.** Micro-X-ray fluorescence maps and micro-X-ray absorption near edge structure spectra locations in Core 2. Maps on the left-hand image are tricolour image of the Al (red), Si (blue), and Ca (green) concentration and maps on the right-hand side are the least squares fitted multi-energy maps with the Ca benzoate, anorthite, and µ-XANES standard.
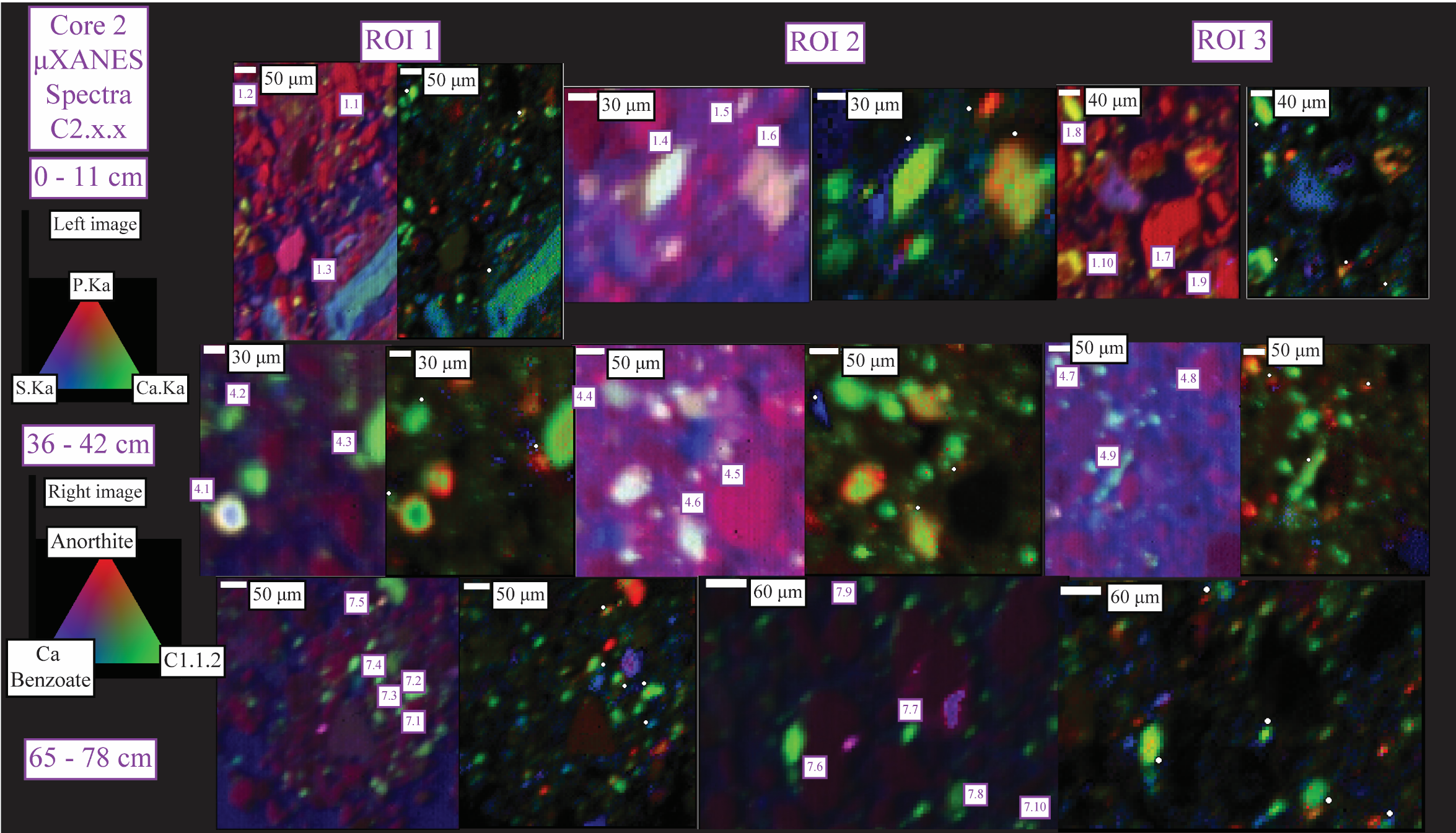
 **Fig. S10.** Micro-X-ray fluorescence maps and micro-X-ray absorption near edge structure spectra locations in Core 2. Maps on the left-hand image are tricolour image of the P (red), S (blue), and Ca (green) concentration and maps on the right-hand side are the least squares fitted multi-energy maps with the Ca benzoate, anorthite, and µ-XANES standard.


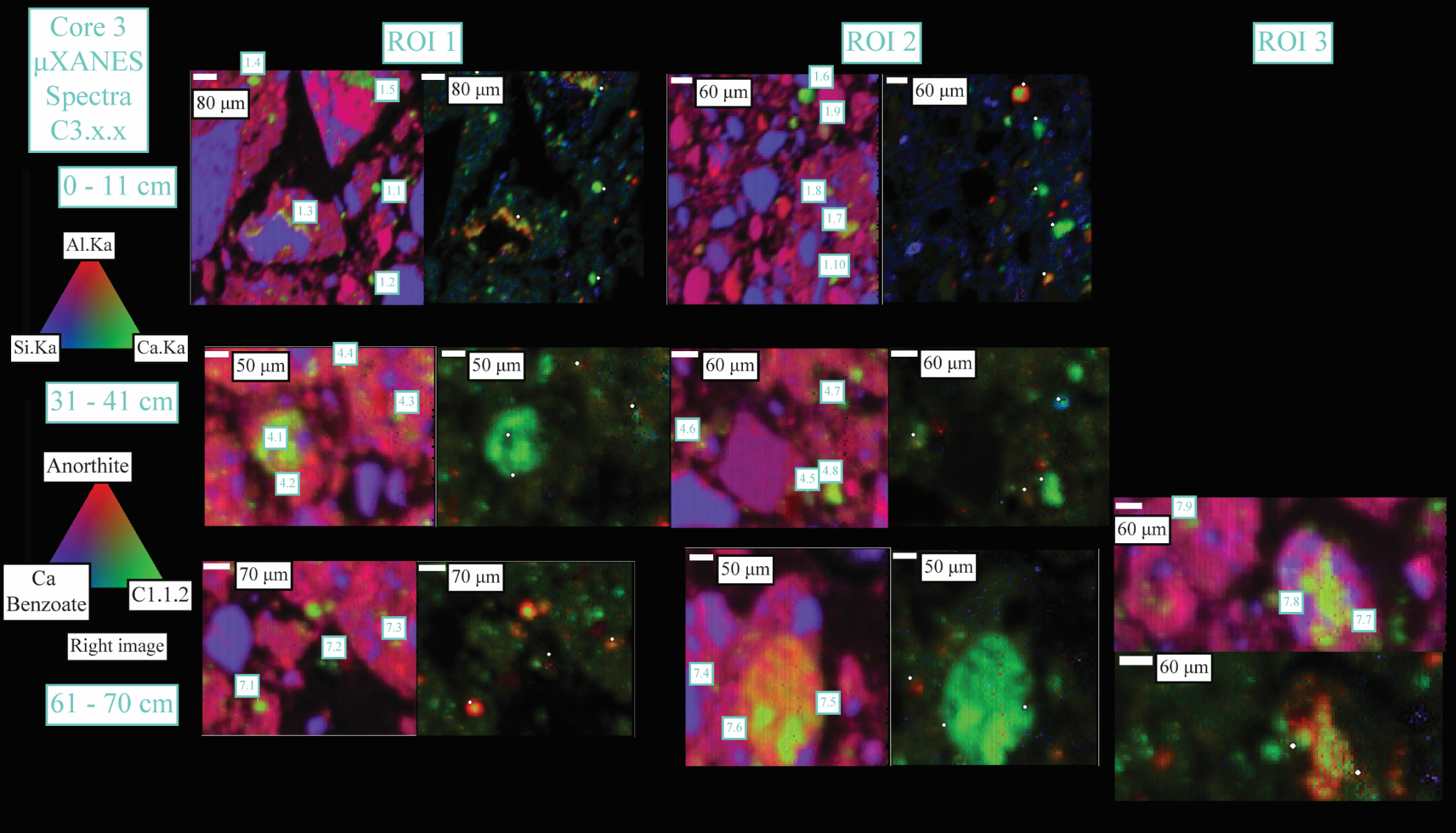


**Fig. S11.** Micro-X-ray fluorescence maps and micro-X-ray absorption near edge structure spectra locations in Core 3. Maps on the left-hand image are tricolour image of the Al (red), Si (blue), and Ca (green) concentration and maps on the right-hand side are the least squares fitted multi-energy maps with the Ca benzoate, anorthite, and µ-XANES standard.


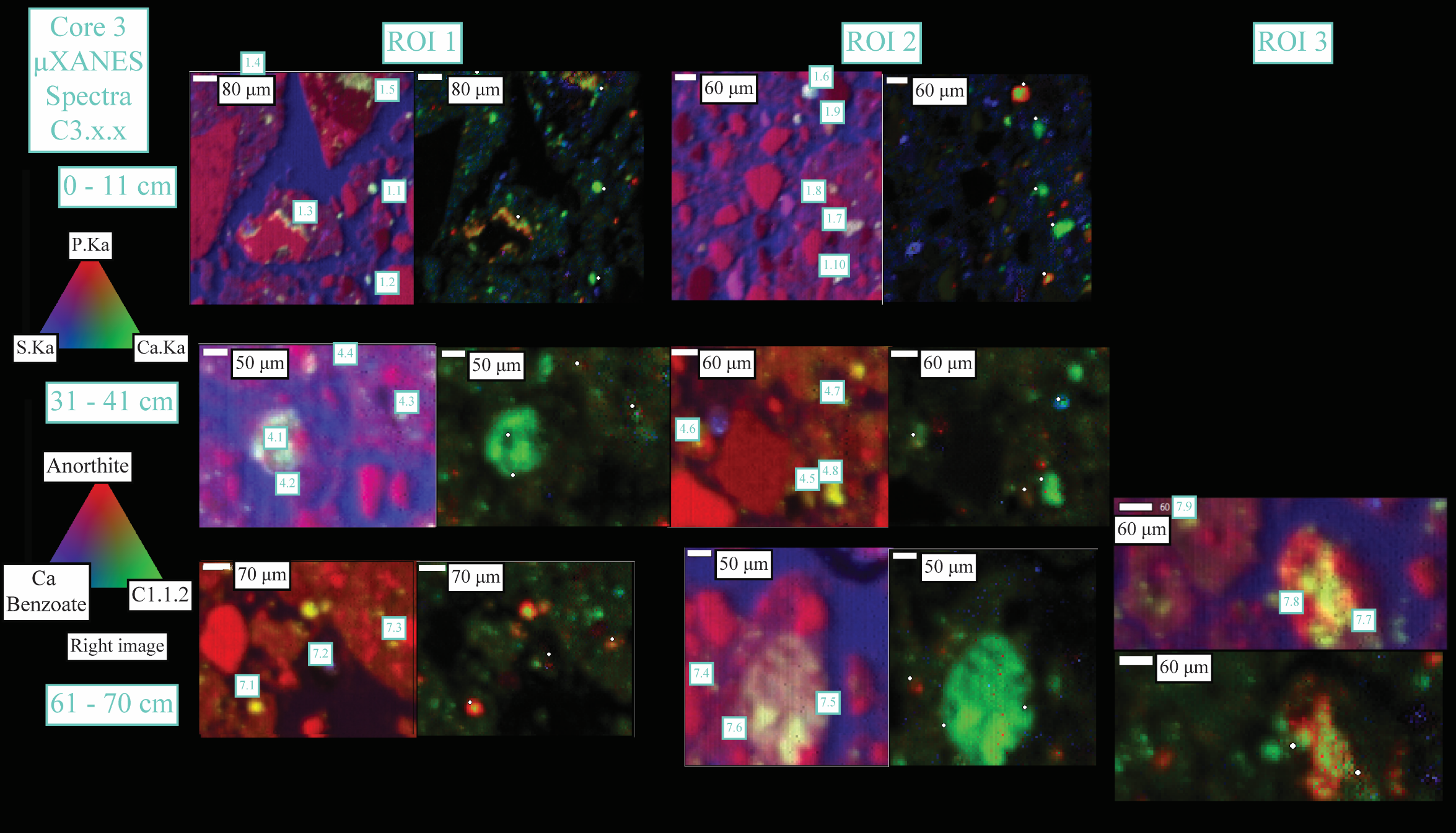


**Fig. S12.** Micro-X-ray fluorescence maps and micro-X-ray absorption near edge structure spectra locations in Core 3. Maps on the left-hand image are tricolour image of the P (red), S (blue), and Ca (green) concentration and maps on the right-hand side a

re the least squares fitted multi-energy maps with the Ca benzoate, anorthite, and µ-XANES standard.

**Table S15.** Elemental correlations between Ca and other elements or least squares fits in the µ-XRF maps. Left hand side of the table lists the slope value for each correlation with Ca and the right hand side lists the adjusted *R*^2^ values for each sample. The adjusted *R*^2^ values between Ca and other measured elements in the µ-XRF maps have been given a graded colour scheme from tan to green with increasing values.

| **Sample** | | **Slope values Ca (x)~** | | | | | | | | | | **R^2^ values Ca~** | | | | | | | | | |
| --- | --- | --- | --- | --- | --- | --- | --- | --- | --- | --- | --- | --- | --- | --- | --- | --- | --- | --- | --- | --- | --- |
|  |  | **µ-XRF elemental correlations** | | | | | | | **LS-fits** | | | **µ-XRF elemental correlations** | | | | | | | **LS-fits** | | |
|  |  | **Na** | **Mg** | **Al** | **Si** | **P** | **S** | **K** | **Pt. Reyes OM** | **Ca benzoate** | **µ-XANES standard** | **Na** | **Mg** | **Al** | **Si** | **P** | **S** | **K** | **Pt. Reyes OM** | **Ca benzoate** | **µ-XANES standard** |
| **Core 1.1** | **ROI 1** | **4.00E-04** | **2.00E-04** | **1.64E-02** | **-1.44E-01** | **1.34E-02** | **4.30E-03** | **2.16E-01** | **1.29E-01** | **7.05E-02** | **8.99E-01** | **0.1** | **0.29** | **0.17** | **0.04** | **0.08** | **0.12** | **0.03** | **0.09** | **0.05** | **0.87** |
|  | **ROI 2** | **6.00E-04** | **1.60E-03** | **9.70E-03** | **-1.86E-01** | **1.60E-03** | **3.00E-03** | **2.19E-01** | **1.23E-01** | **4.16E-02** | **8.19E-01** | **0.17** | **0.22** | **0.11** | **0.12** | **0** | **0** | **0.02** | **0.12** | **0.01** | **0.87** |
|  | **ROI 3** | **4.00E-04** | **1.90E-03** | **1.58E-02** | **4.40E-02** | **2.35E-02** | **4.00E-04** | **1.33E-01** | **1.94E-01** | **1.98E-02** | **8.32E-01** | **0.08** | **0.31** | **0.14** | **0** | **0.22** | **0.11** | **0.02** | **0.22** | **0.01** | **0.88** |
| **Core 1.4** | **ROI 1** | **5.00E-04** | **2.20E-03** | **2.17E-02** | **-7.16E-02** | **1.74E-02** | **2.80E-03** | **3.61E-01** | **7.31E-02** | **1.69E-01** | **8.13E-01** | **0.06** | **0.29** | **0.19** | **0.01** | **0.12** | **0.02** | **0.06** | **0.05** | **0.18** | **0.89** |
|  | **ROI 2** | **6.00E-04** | **1.70E-03** | **1.77E-02** | **-2.70E-01** | **5.30E-03** | **3.70E-03** | **2.51E-01** | **2.51E-02** | **1.27E-01** | **9.72E-01** | **0.15** | **0.25** | **0.27** | **0.15** | **0.02** | **0.16** | **0** | **0.01** | **0.11** | **0.9** |
|  | **ROI 3** | **3.00E-04** | **2.70E-03** | **3.39E-02** | **-1.18E-01** | **1.69E-02** | **2.70E-03** | **5.93E-01** | **3.03E-01** | **1.26E-01** | **7.78E-01** | **0.02** | **0.38** | **0.4** | **0.01** | **0.07** | **0.04** | **0.13** | **0.33** | **0.11** | **0.88** |
| **Core 1.7** | **ROI 1** | **5.00E-04** | **1.70E-03** | **1.43E-02** | **-1.42E-01** | **1.20E-02** | **1.13E-02** | **2.12E-01** | **8.25E-02** | **9.12E-02** | **8.72E-01** | **0.12** | **0.22** | **0.05** | **0.05** | **0.08** | **0.03** | **0** | **0.05** | **0.07** | **0.88** |
|  | **ROI 2** | **5.00E-04** | **1.30E-03** | **7.80E-03** | **-1.92E-01** | **5.30E-03** | **3.10E-03** | **2.39E-02** | **1.30E-01** | **3.72E-02** | **8.03E-01** | **0.29** | **0.41** | **0.1** | **0.12** | **0** | **0.02** | **0** | **0.12** | **0.01** | **0.91** |
|  | **ROI 3** | **9.00E-04** | **2.60E-03** | **2.52E-02** | **-2.53E-01** | **3.20E-03** | **2.00E-03** | **7.97E-01** | **1.79E-01** | **1.88E-01** | **7.45E-01** | **0.11** | **0.22** | **0.23** | **0.06** | **0** | **0** | **0.1** | **0.18** | **0.15** | **0.83** |
| **Core 2.1** | **ROI 1** | **1.00E-04** | **2.30E-03** | **-5.00E-03** | **-1.95E-01** | **3.50E-03** | **1.20E-03** | **1.37E-02** | **4.23E-02** | **2.52E-01** | **9.43E-01** | **0** | **0** | **0.02** | **0.14** | **0.01** | **0.52** | **0** | **0.03** | **0.48** | **0.95** |
|  | **ROI 2** | **4.00E-04** | **1.10E-03** | **2.60E-03** | **-5.57E-02** | **1.52E-02** | **3.80E-03** | **-7.00E-04** | **-1.00E-04** | **-1.96E-02** | **9.31E-01** | **0.43** | **0.42** | **0.02** | **0.06** | **0.53** | **0.38** | **0** | **0** | **0.01** | **0.94** |
|  | **ROI 3** | **0.00E+00** | **1.60E-03** | **1.03E-02** | **-6.66E-02** | **1.60E-02** | **6.40E-03** | **1.63E-01** | **5.95E-02** | **5.27E-02** | **9.36E-01** | **0.08** | **0.36** | **0.11** | **0.01** | **0.17** | **0.09** | **0.11** | **0.06** | **0.05** | **0.95** |
| **Core 2.4** | **ROI 1** | **4.00E-04** | **1.10E-03** | **-8.00E-04** | **-1.04E-01** | **2.17E-02** | **7.30E-03** | **-4.75E-02** | **-1.71E-02** | **2.90E-03** | **8.38E-01** | **0.4** | **0.39** | **0** | **0.15** | **0.49** | **0.53** | **0.01** | **0.02** | **0** | **0.96** |
|  | **ROI 2** | **5.00E-04** | **1.20E-03** | **2.00E-04** | **-1.56E-01** | **1.23E-02** | **5.00E-03** | **3.47E-02** | **-2.13E-02** | **3.90E-03** | **9.52E-01** | **0.38** | **0.41** | **0** | **0.19** | **0.28** | **0.53** | **0** | **0.02** | **0** | **0.96** |
|  | **ROI 3** | **5.00E-04** | **1.50E-03** | **1.60E-03** | **-1.92E-01** | **9.00E-03** | **4.30E-03** | **1.08E-01** | **3.28E-02** | **1.35E-02** | **9.64E-01** | **0.18** | **0.25** | **0** | **0.12** | **0.07** | **0.23** | **0.02** | **0.02** | **0.01** | **0.93** |
| **Core 2.7** | **ROI 1** | **4.00E-04** | **1.70E-03** | **1.22E-02** | **-2.60E-02** | **1.87E-02** | **2.80E-03** | **1.48E-01** | **1.13E-01** | **6.31E-02** | **8.75E-01** | **0.1** | **0.25** | **0.07** | **0** | **0.22** | **0.1** | **0.02** | **0.12** | **0.06** | **0.9** |
|  | **ROI 2** | **6.00E-04** | **1.40E-03** | **8.10E-03** | **-2.24E-01** | **5.00E-04** | **4.60E-03** | **1.85E-01** | **6.20E-02** | **3.71E-02** | **9.26E-01** | **0.17** | **0.15** | **0.05** | **0.06** | **0** | **0.07** | **0.05** | **0.06** | **0.04** | **0.95** |
| **Core 3.1** | **ROI 1** | **2.00E-04** | **2.10E-03** | **1.33E-02** | **-8.34E-02** | **1.48E-02** | **1.90E-03** | **2.08E-01** | **1.04E-01** | **9.44E-02** | **9.15E-01** | **0.02** | **0.45** | **0.11** | **0.01** | **0.08** | **0.03** | **0.13** | **0.12** | **0.14** | **0.94** |
|  | **ROI 2** | **6.00E-04** | **1.70E-03** | **9.90E-03** | **-1.67E-01** | **1.03E-02** | **5.20E-03** | **2.49E-01** | **7.73E-02** | **5.57E-02** | **8.82E-01** | **0.17** | **0.25** | **0.06** | **0.05** | **0.06** | **0.2** | **0.04** | **0.07** | **0.04** | **0.92** |
| **Core 3.4** | **ROI 1** | **1.00E-04** | **2.00E-03** | **1.57E-02** | **-2.24E-02** | **1.94E-02** | **5.00E-04** | **1.10E-01** | **1.61E-02** | **1.45E-02** | **1.07E+00** | **0.01** | **0.38** | **0.19** | **0** | **0.23** | **0** | **0.04** | **0** | **0.02** | **0.95** |
|  | **ROI 2** | **5.00E-04** | **2.00E-03** | **1.37E-02** | **-2.19E-01** | **7.20E-03** | **6.30E-03** | **2.96E-01** | **8.62E-02** | **1.04E-02** | **9.92E-01** | **0.09** | **0.29** | **0.11** | **0.06** | **0.02** | **0.06** | **0.21** | **0.07** | **0.01** | **0.93** |
| **Core 3.7** | **ROI 1** | **-3.00E-04** | **2.90E-03** | **2.94E-02** | **4.20E-02** | **2.15E-02** | **0.00E+00** | **3.77E-01** | **1.94E-01** | **1.26E-02** | **8.01E-01** | **0.03** | **0.37** | **0.28** | **0** | **0.16** | **0** | **0.2** | **0.28** | **0.02** | **0.91** |
|  | **ROI 2** | **4.00E-04** | **2.50E-03** | **1.83E-02** | **-2.22E-02** | **2.12E-02** | **2.20E-03** | **4.71E-01** | **-2.50E-02** | **-8.00E-04** | **9.92E-01** | **0.14** | **0.67** | **0.42** | **0** | **0.45** | **0.08** | **0.64** | **0.04** | **0** | **0.98** |
|  | **ROI 3** | **1.00E-04** | **2.00E-03** | **1.46E-02** | **9.35E-02** | **2.82E-02** | **3.00E-04** | **1.40E-01** | **6.56E-02** | **2.30E-03** | **8.77E-01** | **0.01** | **0.48** | **0.21** | **0.07** | **0.6** | **0** | **0.15** | **0.08** | **0** | **0.95** |
| **Average** | | **4.00E-04** | **1.80E-03** | **1.28E-02** | **-1.14E-01** | **1.33E-02** | **3.50E-03** | **2.19E-01** | **8.45E-02** | **6.10E-02** | **8.93E-01** | **0.14** | **0.14** | **0.32** | **0.14** | **0.06** | **0.17** | **0.14** | **0.08** | **0.09** | **0.07** |


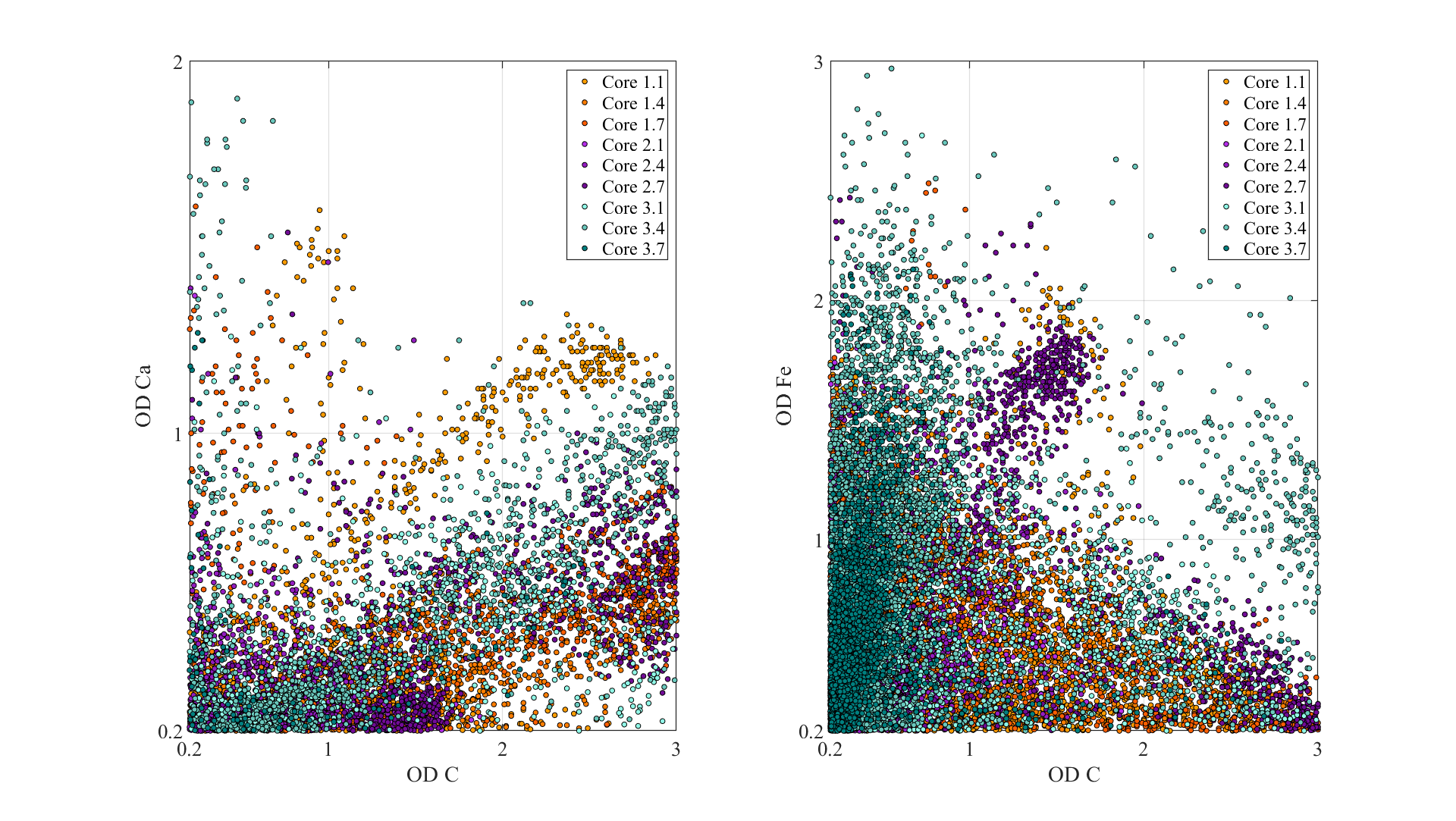


**Fig. S13.** The linear correlation between optical density pixel values for Ca and C in the left-hand image and Fe and C in the right-hand image.


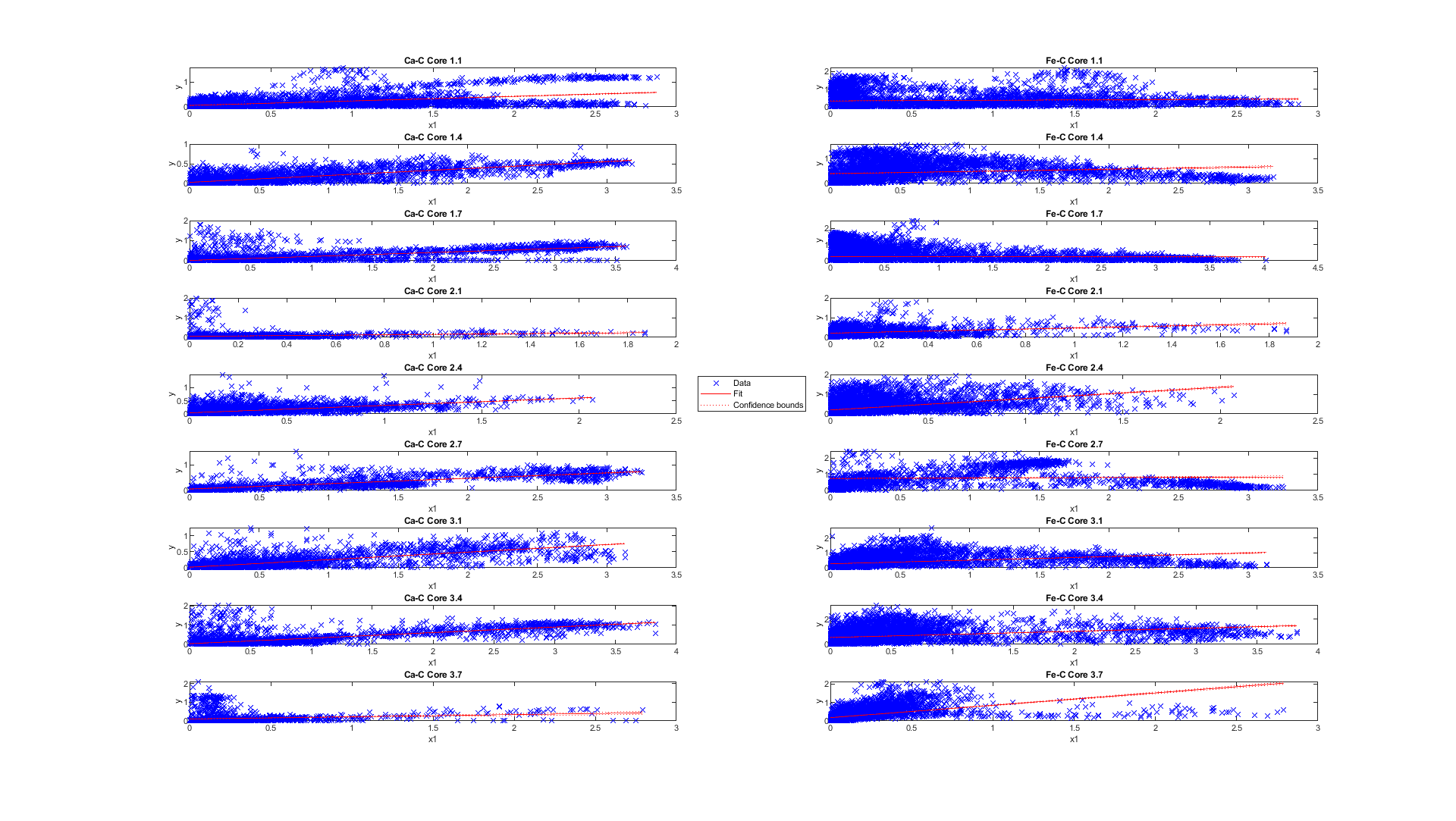


**Fig. S14.** The linear correlation between optical density pixel values of Ca and C (left) and Fe and C (right) for each sample individually.

**Table S16.** Calcium L-edge STXM spectra *L*_2_ and *L*_3_ peak locations and spitting ratio (difference between the two) from the spectra subset by their elemental association. All numbers are in eV.

| **Elemental association** | **L3** | | | **L2** | | |
| --- | --- | --- | --- | --- | --- | --- |
|  | **Sub** | **Principal** | **Splitting ratio** | **Sub** | **Principal** | **Splitting ratio** |
| **Fe-C-Ca** | 348.1 | 349.2 | 1.1 | 351.3 | 352.5 | 1.2 |
| **Ca-Fe** | 348 | 349.2 | 1.2 | 351.3 | 352.5 | 1.2 |
| **Ca-C** | 348.15 | 349.2 | 1.05 | 351.45 | 352.5 | 1.05 |
| **Overall Ca** | 348.1 | 349.2 | 1.1 | 351.4 | 352.5 | 1.1 |


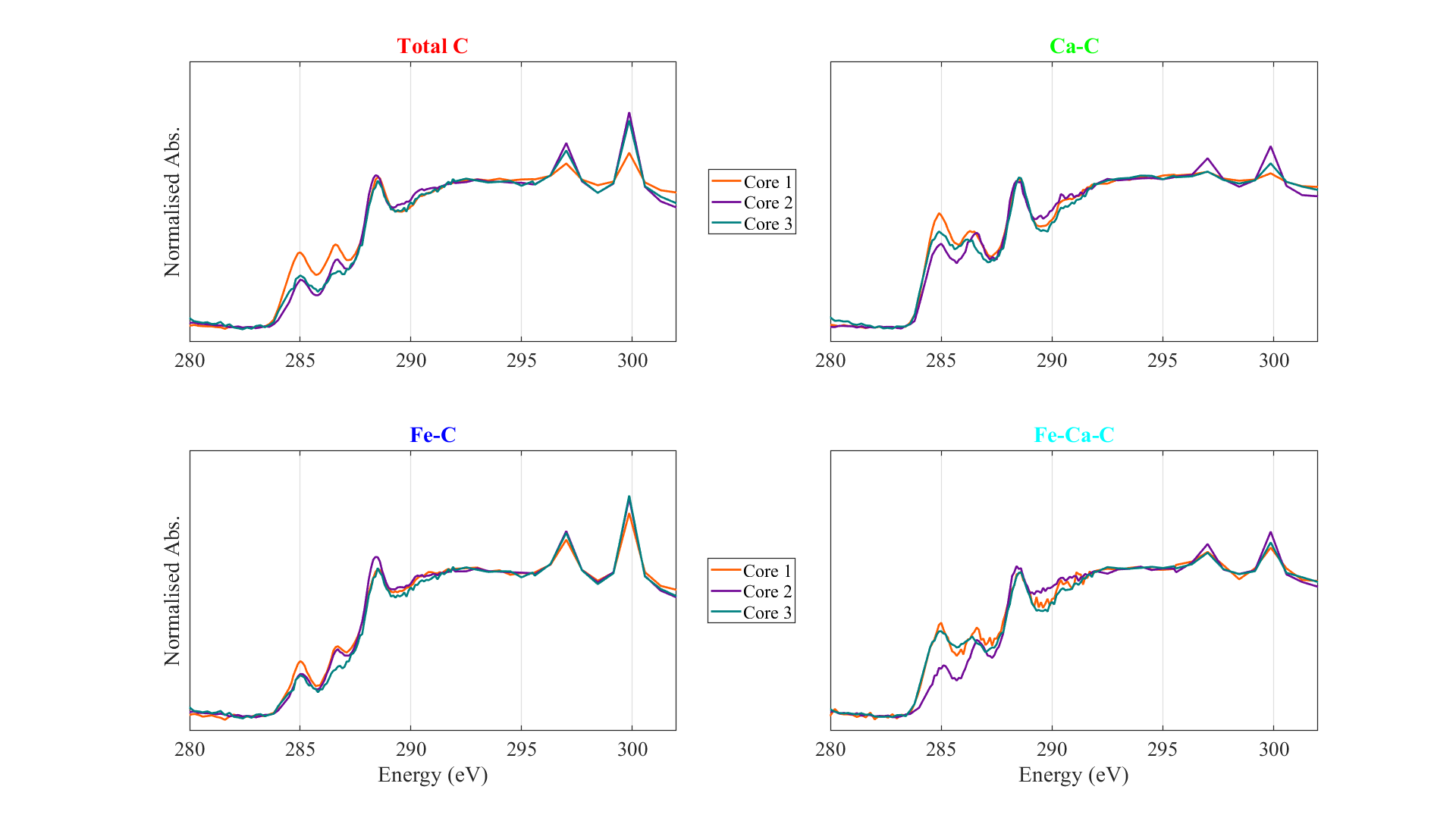


**Fig. S15** The C K-edge spectra of each elemental subset averaged over each core sample for the overall SOC signal (Total C), calcium-carbon (Ca-C), iron-carbon (Fe-C), and iron-calcium-carbon (Fe-Ca-C).


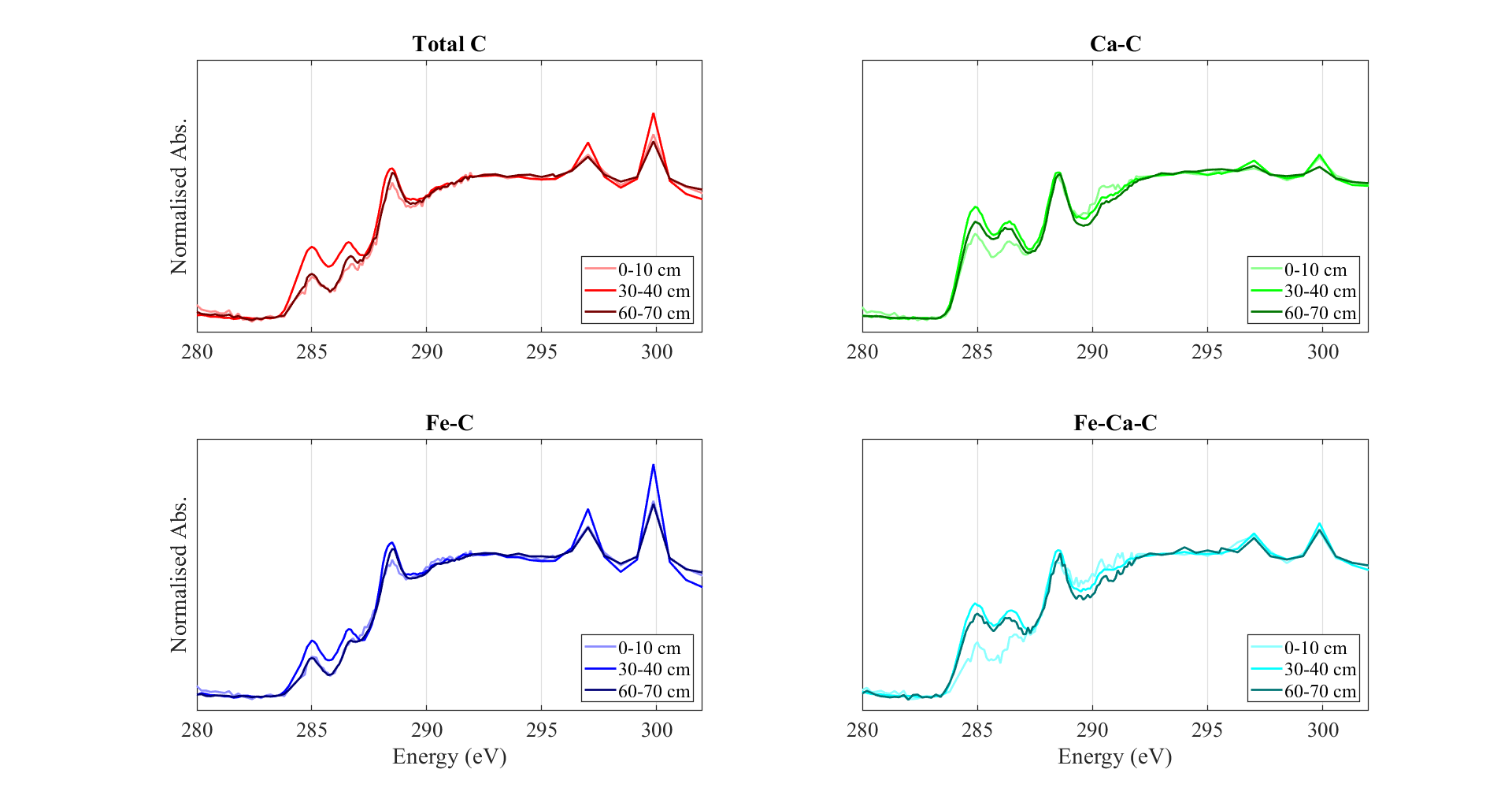


**Fig. S16.** The C K-edge spectra of each elemental subset averaged over each depth for the overall SOC signal (Total C), calcium-carbon (Ca-C), iron-carbon (Fe-C), and iron-calcium-carbon (Fe-Ca-C).


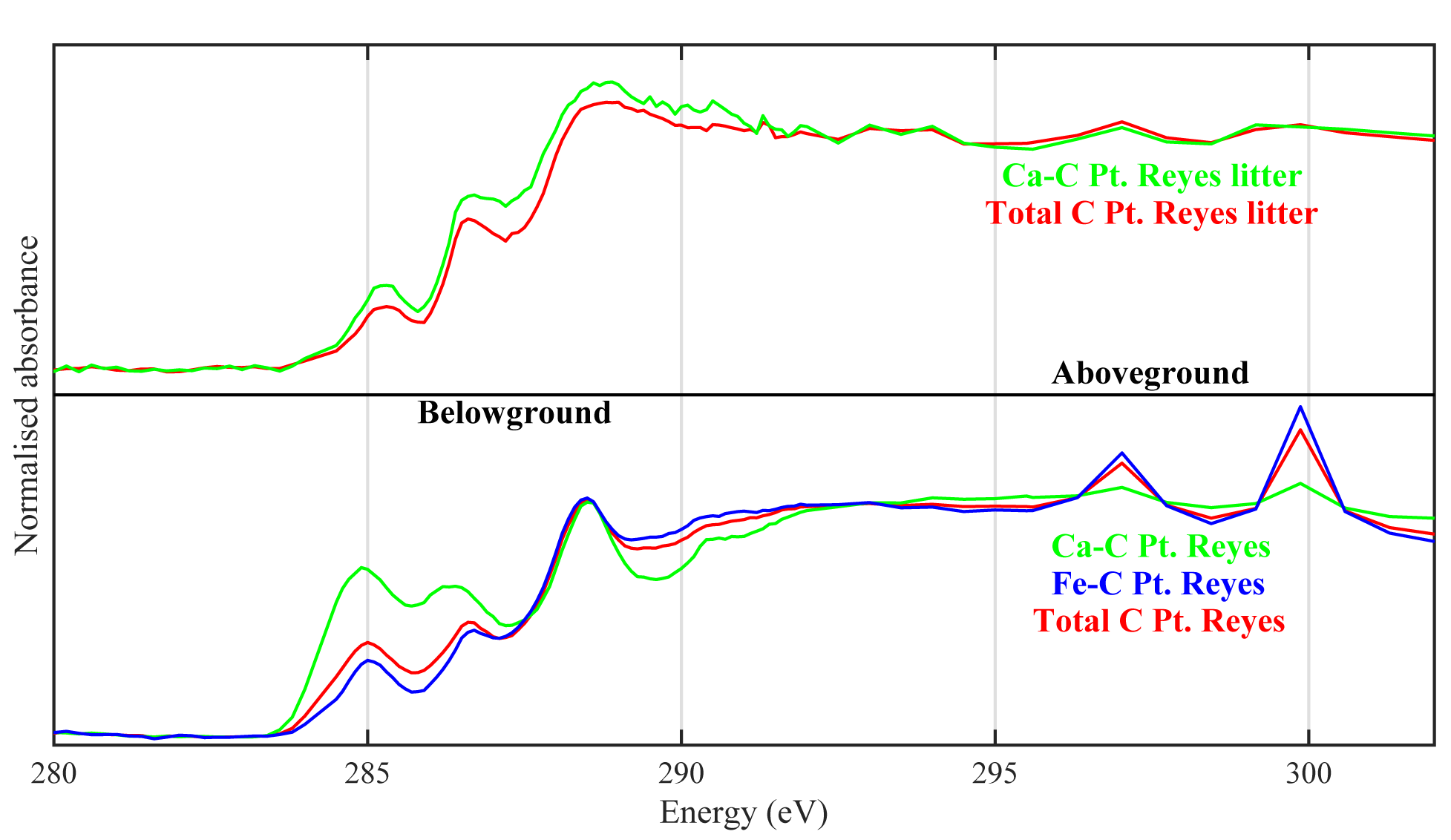


**Fig. S17.** A comparison of aboveground (top) and belowground (bottom) C K-edge spectra. Aboveground spectra are taken from stacks on Point Reyes litter standard measurements at ALS BL 5.3.2.2, while the belowground spectra are averaged elemental associations taken over all our soil core sample stacks (also at ALS BL 5.3.2.2.).

**
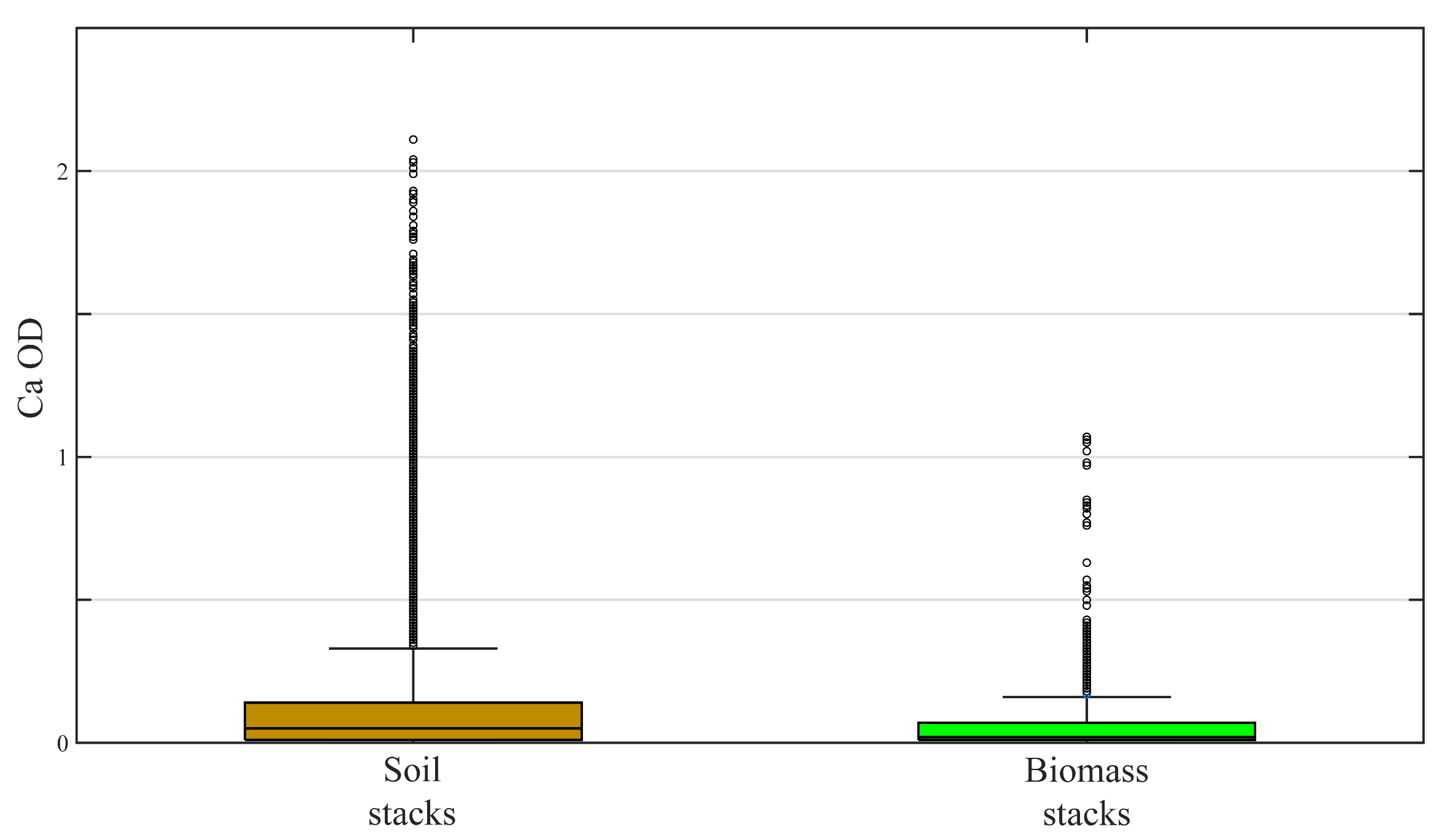
**

**Fig. S18.** Boxplot of the optical density (OD) values of Ca in the STXM NEXAFS soil sample stacks (*n* = 460004) and Pt. Reyes biomass sample stacks (*n* = 18527).


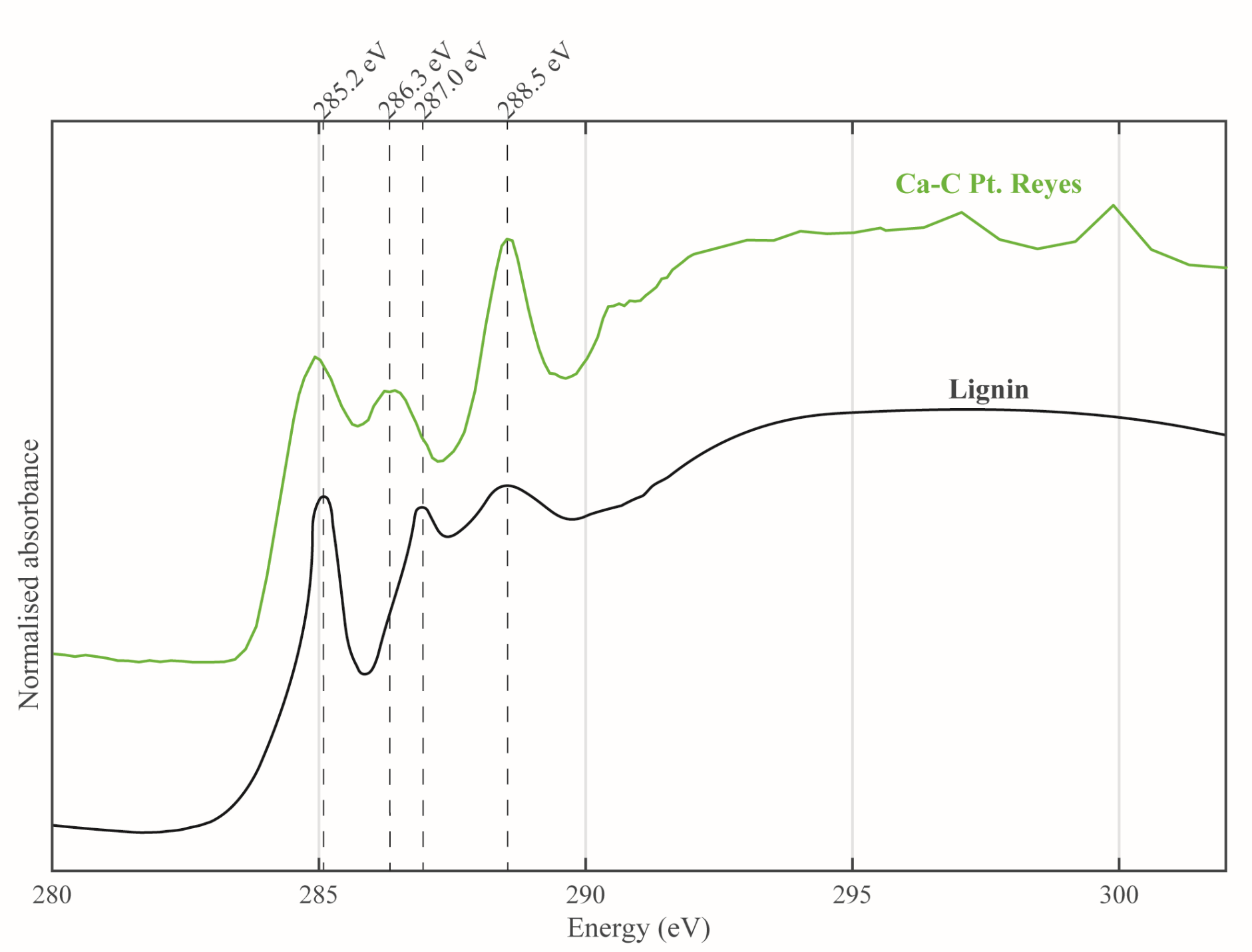


**Fig. S18.** A comparison of a lignin STXM C NEXAFS spectra and Ca-C spectra taken from stacks averaged over each of our soil cores measured at 3 depth intervals (both samples recorded at ALS BL 5.3.2.2.). The lignin spectra is digitalised from spectra presented in Karunakaran et al. (2015) with permission from Dr. Chithra Karunakaran.
